# Supplementary material for: Meta-analysis across six global biobanks identifies recessive coding associations with complex traits and diseases
Source: Am J Hum Genet. 2026 May 1;113(6):1330–46. doi: 10.1016/j.ajhg.2026.04.005 (PMC13277689; doi:10.1016/j.ajhg.2026.04.005)
Supplement: Document S1. Figures S1–S11 and Notes S1–S9 [file mmc1.pdf]

The American Journal of Human Genetics, Volume *113*

Supplemental information

Meta-analysis across six global biobanks identifies recessive coding associations with complex traits and diseases

Frederik H. Lassen, Georgios Kalantzis, Andrea Eoli, Barney Hill, Kyuto Sonehara, Shinichi Namba, Isaac Wade, Sam Hodgson, Wei Zhou, BioBank Japan Project, Genes & Health Research Team, BRaVa Consortium, Benjamin M. Neale, Konrad J. Karczewski, Yukinori Okada, David A. van Heel, Sarah Finer, Cecilia M. Lindgren, Henrike O. Heyne, Hilary C. Martin, and Duncan S. Palmer

Supplemental Material and Methods

Supplemental Notes

Note S1. Phenotyping and case counts across biobanks

As a data quality check, we compared the disease burden across biobanks (Fig. S1). Most UK-based cohorts showed high correlation in phenotype prevalence, e.g. r = 0.95 for UKB-100kGP, or r = 0.75 for 100kGP-G&H. Phenotype prevalence in AOU also showed high correlation with most other biobanks e.g. 0.83 with BioMe, or 0.81 with UKB. Prevalence in all three UK-based cohorts had low correlation with BBJ (maximum Spearman correlation r = 0.44), which is potentially a reflection of the fact that the UK studies incorporate both primary and secondary care data, while BBJ is a hospital-based cohort, enriched for sick individuals, as well as having a distinct ancestry composition[1]. Nonetheless, the general high concordance suggests a robust overall approach to case identification across the consortium.

We investigated specific instances where disease incidence differed markedly across biobanks, likely reflecting both established epidemiological trends and distinct characteristics of each biobank. A striking example is the case of Type 2 Diabetes (T2D) cases. The global adult T2D prevalence is projected to reach 7.7% by 2030[2], which is significantly lower than the rates we observed in our study e.g. 25.7% in G&H, or 8.6% in UKB. This disparity aligns with the documented higher prevalence of T2D in South Asian populations[3,4] and aging individuals[5], as well as in diverse urban settings[6,7]. Further examples underscore how biobank-specific factors can significantly influence observed patterns of disease occurrence. Asthma prevalence demonstrates this clearly: while the global prevalence was around 3.3% in 2021[8], we observed markedly different rates across biobanks. BBJ had only 0.8% (100 cases) with asthma codes, far below Japan’s population prevalence of 3.5%, likely because only very severe cases have these codes recorded in a hospital setting. In contrast, UKB and G&H, which had primary care as well as secondary care records, had much higher rates at 9.7% and 14.7%, respectively, whereas the UK average is 10.0%. Similarly, cancer prevalence patterns reflect biobank-specific recruitment strategies. In 100kGP, 7.3% (4,276 cases) were diagnosed with breast cancer, nearly double the 3.7% observed in UKB. This high rate aligns with 100kGP’s focus on cancer patients as part of its rare disease and

cancer sequencing initiative[9,10]. These examples highlight how the design and focus of each biobank can lead to significant variations in disease prevalence.

Note S2. Preprocessing for the 100,000 Genomes Project

The following workflow is adapted from Genomics-England's documentation available online. To avoid computationally cumbersome recalling of variant calls in WGS from 100kGP, and in order to harmonise the processing perfectly with other datasets, we leveraged the quality-controlled Aggregated Variant Call (AggV2) dataset, a multi-sample variant call format (VCF) comprising 78,195 germline genomes and 722 million SNPs and small indels (≤ 50bp) upon release. This dataset was constructed by merging single-sample gVCF files, with sample and variant-level quality control described below. Samples were sequenced on Illumina HiSeq X instruments using 150bp paired-end reads, one lane per sample. Data processing employed the Illumina North Star v4 Whole Genome Sequencing Workflow (NSV4, v2.6.53.23), using iSAAC Aligner (v03.16.02.19) and Starling Small Variant Caller (v2.4.7). Reads were aligned to the Homo sapiens NCBI GRCh38 assembly with decoys. Samples were individually quality controlled and required to meet the following criteria:

- Sample contamination (freemix) < 0.03
- Ratio of single nucleotide polymorphism (SNV) heterozygous to homozygous calls
- Total number of SNVs between 3.2M-4.7M
- Array concordance > 90%
- Median fragment size > 250bp
- Excess of chimeric reads < 5%
- Percentage of mapped reads > 60%
- Percentage of AT dropout < 10%

Additionally, samples were required to have at least 95% of the genome covered at 15x or above with well mapped reads (mapping quality > 10) after discarding duplicates. Variants were split from multi-allelic into bi-allelic and indels were left-aligned using vt v0.57721. Variants were filtered based on the following criteria:

- Missingness ≤ 5%
- Median depth ≥ 10
- Median genotype quality ≥ 15
- ≥ 25% of heterozygous calls showing no significant allele imbalance
- ≥ 50% of sites with complete genotype data

- HWE mid *p*-value ≥ 10^{-5} in unrelated samples of inferred European ancestry

Finally, sex-specific QC was performed for the X chromosome. In summary, 722,342,407 (74.8%) autosomal variants passed all QC filters.

Statistical Phasing

For the 100kGP we used phased genotypes generated by Shi et al.[11] using SHAPEIT4, who performed additional variant- and sample-level quality control prior to phasing. The following description is adapted from their work. This quality control process evaluated genotype quality, depth, missingness, allelic balance, Mendelian errors, HWE equilibrium, and gnomAD[12] allele frequency concordance. Shi et al. removed singletons in unrelated individuals, as these could not be phased statistically using SHAPEIT4. Overall, these stringent filters reduced the number of variants from 722M to 342M.

The phasing strategy, as described by Shi et al., involved a multi-step strategy that exploited familial relationships within 100kGP. First, they used duos and trios to obtain phase by direct transmission in offspring. Using those phased haplotypes as scaffold, they then phased the remaining unphased genotypes in related samples using SHAPEIT4 (v4.2.2). Then, using all phases as scaffold, they phased common variants (MAF>0.01) for unrelated samples using SHAPEIT4. Finally, the remaining rare variants in unrelated samples were phased using the phased common variants as a scaffold, and any phased related samples as a reference panel. Phasing of related individuals was conducted at the chromosome level, while the latter step was carried out in regions of approximately 300,000 sites, with 30,000 sites on each side as a buffer. The resulting phases for regional segments were merged and concatenated using the ligate tool from bcftools. Shi et al. assessed phasing accuracy by evaluating the performance of 100kGP as a reference panel through genotype imputation in 589 trios from the 1000 Genomes Project, using SHAPEIT4. Phasing accuracy was then evaluated by comparing imputed haplotypes to those inferred from Mendelian inheritance patterns in each trio.

Phenotyping and additional control exclusions in 100kGP

We excluded probands in 100kGP diagnosed with either global developmental delay (n = 12,191), intellectual disability (n = 9,149), or autism (n = 4,609), identified through manual curation of ICD and Human Phenotype Ontology (HPO) codes. We refer to these terms collectively as NDD. We deemed this exclusion necessary for two reasons: firstly, NDD patients in our cohort were predominantly very young (median age 15 years), making them more likely to be classified as controls for many common adult-onset diseases investigated

here. Secondly, NDD patients are likely to be enriched for rare bi-allelic variants[13]. The combination of these factors could potentially lead to an artificial enrichment of rare bi-allelic variants in our control group, risking spurious associations where the absence of these variants might appear to increase disease risk. To mitigate this potential bias, we excluded a total of 13,976 NDD probands, reducing our sample size to 69,513.

Note S3. Assessment of phasing quality across cohorts

We evaluated phasing accuracy across multiple biobanks and ancestries (EUR, SAS, and EAS) using complementary strategies tailored to the available data (Sup. Figure 2 and Sup. Table 11).

For UKB and G&H, we leveraged 99 and 100 parent–offspring trios, respectively, to estimate switch-error rates (SER) after statistical phasing. Across all variants, the average SER was low, with 0.16% in UKB and 0.35% in G&H, indicating overall high-quality statistical phasing. As expected, rare variants showed elevated error rates: singletons (MAC = 1) exhibited the highest SER, reaching 31.77% in UKB and 24.29% in G&H, with errors decreasing steadily as MAC increased. Importantly, restricting to high-confidence phased genotypes (posterior probability, PP > 90%), which is what we used in all downstream analyses, substantially reduced the SER, with 4.12% and 5.63% for MAC = 1 in UKB and G&H respectively, providing reassurance that phasing quality for rare variants is well controlled after posterior filtering.

In BBJ, where trio data were not available, we benchmarked statistical phasing against read-backed phasing from short-read sequencing calls using WhatsHap[14]across 1000 randomly sampled individuals of EAS ancestry. We then used custom R scripts to determine the agreement between read-backed phased and statistically inferred haplotypes. This process involved identifying pairs of variants in close proximity (150 - 250bp) on short-read sequences from .bam or .cram files using WhatsHap[14] with default parameters. Two variants on the same short-sequencing read must originate from the same haplotype (in *cis*), while variants on different reads are likely to originate from opposite haplotypes (in *trans*). Given this, phasing accuracy was estimated as the proportion of read-backed configurations of variant pairs that agreed with the configuration of statistically inferred variants. Similarly to UKB and G&H, error rates were highest for singletons, at 38.26%, but decreased rapidly for low-frequency variants (e.g. 4.36% for 1<MAC<5). Applying the same PP > 90% criterion yielded markedly improved error rates, with an average of 0.30% across all variants. This demonstrates that posterior filtering effectively enriches for correctly phased haplotypes in

BBJ as well, supporting the robustness of our phasing strategy across cohorts and ancestries.

Note S4. Gene knockouts and comparison with other studies

To date, several studies have systematically identified and reported rare predicted bi-allelic LoF individuals, collectively encompassing 7,149 unique autosomal genes with knockouts (KO hereafter)[12,15–18]. Sun et al.[19], the most recent study, observed 4,848 genes in a sample of 983,578 individuals, considering pLoF variants with MAF < 1%. Following these studies, we set out to survey pLoF (HC by LOFTEE; Methods) variants in 19,334 unique human protein-coding genes across our six biobanks, to identify bi-allelic genotypes, using MAF < 1% to match the literature; this threshold is different to the one for our recessive association study for which we used a more lenient threshold of 5% to ensure sufficient numbers of bi-allelic genotypes.

While working with SHAPEIT5 for statistical phasing, we noticed that missing genotypes (if any) are imputed during the integral early steps of the program. We quantified the rate of missing-then-imputed pLoF-homozygotes and found it to be between 0.1% and 2% across cohorts. Although these rates would imply a miniscule effect in our association study – and many of these genotypes might be correctly imputed anyway – we decided to exclude these from our KO analysis to avoid reporting genes with false knockouts. Thus, for this analysis, we used the post-QC unphased data to ascertain homozygotes and the phased data for compound-heterozygotes.

We found a total of 5,563 genes harbouring KOs (Sup. Table 4) across chromosomes 1-22. Of these, 2,293 (41.2%) distinct genes had a bi-allelic pLoF in a single individual, as expected since pLoFs tend to be rare. Of the 3,270 genes observed with two or more bi-allelic individuals, the majority (n = 1,265, 38.7%) were restricted in two biobanks, whereas only 195 (6.0%) were observed in five or six biobanks. G&H yielded the highest number of unique genes with bi-allelic pLoF genotypes (2,519 genes), surpassing the whole of UKB (2,077 genes), despite a roughly x10 difference in sample size (39k vs 395k), due to the higher autozygosity[20]. In contrast, when considering specifically CH variation, UKB:EUR contributed the highest number of unique genes (827 genes), followed by 100kGP:EUR (431 genes), and then G&H (257 genes). Notably, 62.9% (1111/1767) of the novel KO genes were detected in individuals of SAS ancestry. Overall, these observations show how sample demography and ancestry composition – particularly autozygosity in consanguineous populations – profoundly shape the landscape of bi-allelic loss-of-function variation.

Note S5. Testing summary statistics for inflation

We assessed the quality of summary statistics by calculating two genomic inflation metrics - λ_{GC} and λ_{95}, calculated at the 50th and 95th percentiles of the test statistic distribution, respectively - across 41 traits, six biobanks, four variant annotation categories, and any available ancestry. λ_{95} is considered more appropriate for rare variant studies, where test statistics are often deflated due to sparse data[21]. Indeed, considering all recessive tests, the distribution of λ_{95} values was closer to one compared to that of λ_{GC} values, across all annotations (Fig. S3), making that a better choice to test for inflation. As a reference, both inflation factors had a similar distribution on additive tests where there is less sparsity; for example both had a mean value of 1.02 for nonsynonymous tests (Fig. S4-S5). Overall, 1,604 of the 1,630 (98.4%) recessive analyses across all biobanks, ancestries and annotations had $\lambda_{95} < 1.25$, indicating sufficient control of test-statistic inflation.

Next, we assessed the inflation factors after the meta-analysis and the Cauchy combination and found no indication of inflation, with a mean $\lambda_{95} = 1.00$ and mean $\lambda_{GC} = 0.93$ (Fig. S6). All phenotypes showed $\lambda_{95} < 1.07$, except height ($\lambda_{GC} = 1.15$, $\lambda_{95} = 1.20$), which was the one yielding the highest number of significant associations (eleven with $p_{rec} < 7.53 \times 10^{-7}$). This is perhaps expected given the well-documented polygenic architecture[22,23] and association with autozygosity[24].

Finally, as a negative control and an additional test of calibration, we performed a meta-analysis of synonymous variant burdens under the recessive model. Similarly to the cauchy results described above, all phenotypes showed calibrated λ_{95} for the synonymous tests with a mean of 0.99 and range [0.43, 1.17]. This analysis yielded five gene-trait associations significant at FDR < 0.01 (see Sup. Table 6 for more details), all of which showing more significant additive than recessive effects ($p_{add} < p_{rec}$). Importantly, all cases have support from recent association studies, suggesting these signals likely reflect linkage disequilibrium with known associations involving common variants, and the synonymous variant may well not be the causal SNP. For instance, we associate *CEACAM19* with LDL cholesterol ($p_{rec} = 1.65 \times 10^{-20}$; $p_{add} = 2.59 \times 10^{-234}$); 95% of the 254 bi-allelic individuals in this gene were homozygous for rs76075198, a high-frequency (MAF=2.5% in UKB:EUR) variant which was previously associated with LDLC[25]. Similarly, we associate *TOMM40* with C-reactive protein and ($p_{rec} = 5.48 \times 10^{-12}$; $p_{add} = 1.46 \times 10^{-194}$), a gene in which an intron variant rs34095326 has been reported in the GWAS Catalog. Lastly, we associate *CCHCR1* with height ($p_{rec} = 3.42 \times 10^{-8}$; $p_{add} = 5.98 \times 10^{-41}$), a gene with several intron variants in the

GWAS Catalog (e.g. rs2073717), all of which are near high-frequency synonymous variants in our cohort (e.g. rs130077).

Note S6. Significant associations that are likely to be driven by known rare recessive disorders

We found recessive associations between COPD and *ODAD1* ($p_{rec} = 4.8 \times 10^{-7}$), as well as a 100kGP-specific signal for *DNAI1* (pLoF|damaging_misense $p_{rec} = 8.98 \times 10^{-10}$) which did not reach significance after meta-analysis (Cauchy $p_{rec} = 5.26 \times 10^{-6}$). These two genes are known to be recessive causes of primary ciliary dyskinesia (MIM: 615038 and 604366;[26–28]) and we suspected that the associations with COPD might be due to misdiagnosis. We reviewed medical records in 100kGP and found that of the 17 individuals of damaging bi-allelic variants in these two genes, 11 had COPD of which 10 had also PCD. When conditioning COPD association tests on PCD status in 100kGP, the signals were attenuated ($p_{rec} > 0.02$), suggesting potential misclassification due to overlapping clinical features. Of note, all PCD cases (n=50) in 100kGP who were included as COPD cases were classified as such due to having the J47 (bronchiectasis) code, versus 956 of all 3,801 COPD (25%) cases; in contrast, 72% (n=2,719) of all COPD cases had J44 (“Other chronic obstructive pulmonary disease”) versus only 6 out of 131 (5%) PCD cases. This supports the notion that these individuals with PCD in 100kGP certainly do not have ‘typical’ COPD and may have been misdiagnosed. Thus, it is likely that recessive variants in *ODAD1* and *DNAI1* do not associate with COPD independently of their effects on PCD.

We also detected an association between levels of aspartate aminotransferase (AST) and *PYGM* ($p_{rec} = 8.17 \times 10^{-10}$), driven by individuals of European ancestry from UKB and AOU. The association was yielded by pLoF variants, whereas the signal attenuated after considering missense variants (pLoF|damaging_misense $p_{rec} = 3.62 \times 10^{-2}$; nonsynonymous $p_{rec} = 8.98 \times 10^{-2}$). Recessive variants in *PYGM* cause Glycogen Storage Disease V (commonly known as McArdle disease), a disorder of glycogen metabolism leading to muscle damage after exercise, which could result in the release of AST into the bloodstream[29]; indeed, by checking medical records we confirmed that 60% of *PYGM* bi-allelic pLoF individuals (exact numbers can not be reported due to privacy constraints in AOU) were diagnosed with glycogen storage disease (ICD:E74). When adjusting for diagnosis of this disease, we observed a notable attenuation, with the association becoming non-significant after meta-analysis ($p_{rec} = 5.52 \times 10^{-6}$; Sup. Table 8). We thus conclude that the elevation of AST likely derives from McArdle-related rhabdomyolysis rather than from primary liver pathology.

Note S7. Alternative ways to decide on recessive over additive associations

In our main analysis we classify recessive over additive effects by comparing P-values from the corresponding models, and by specifically assessing when $p_{rec} < p_{add} / 100$ (the "100x rule"). This was based on previous work by Heyne et al.[30] who performed simulations with 200k individuals and different MAF thresholds and found that, among the recessive tests that were significant ($p_{rec} < 5.0 \times 10^{-8}$), the 100x rule had a true positive rate of 1.00 to identify recessive over additive effects (i. e. almost no false negatives). Likewise, precisely zero simulated-additive effects were identified as recessive (i.e. no false positives). We thus set out to use this rule in our analysis of recessive gene burden.

As an alternative approach, we explored a more comprehensive rule by also considering effect sizes, to handle cases where the additive model might be under-powered, or when the additive effect might have a similar magnitude to the recessive one. To that end, we compared the difference in the ($\log_{10}$) P-values to the ratio of effect size estimates (Sup. Fig 10). An alternative classification of recessive versus additive associations could thus be:

- Recessive if $p_{rec} < p_{add} / 100$ AND ($beta_{rec} / beta_{add} > 1.5$ OR $p_{add} > 0.05$),
- Additive if $p_{add} < p_{rec} / 100$ AND $beta_{rec} / beta_{add} < 1.5$,
- Ambiguous otherwise.

Applying this rule to the results of our meta-analysis would result in 16 putatively recessive associations, fully overlapping with our original 17 (Table 2), except for the ambiguous case of IBD - *NOD2*, a gene known for mixed inheritance (MIM: 605956). Other ambiguous cases include putatively dominant effects, or associations with strong recessive effects but more significant additive P-values, such as *FYCO1* - AST ($\beta_{rec} = 0.28$; $\beta_{add} = 0.10$) or *ABHD15* - Height ($\beta_{rec} = 0.27$; $\beta_{add} = 0.07$); see Sup. Table 5 for detailed estimates. The effect-size ratio threshold of 1.5 was chosen as a conservative margin to try to distinguish true recessive effects where the additive effect estimate is inflated or the recessive effect estimate is deflated due to sampling error, but similar thresholds would yield roughly the same classification, particularly for recessive ones.

Note S8. Refining association signals without P/LP variants

To help answer whether our recessive associations are due to variants with well-established recessive effects, or due to novel effects, we repeated association testing after using information from ClinVar[31]. Specifically, we extracted any variant being pathogenic (P) or

likely pathogenic (LP) in ClinVar (November 2025 release) across the genes involved in our recessive associations (Table 2), and matched these with all variants observed in our data based on the same reference:alternate allele. We then identified any individuals being homozygotes for one, or CH for two of such P/LP variants, and devised new recessive burdens to test for association, focusing on genes showing nominally-significant results ($P < 0.05$) in the first round of analysis at AOU, G&H and 100kGP.

The new set of gene burdens were drastically sparser than the original one, in many cases resulting in no genotypes at all, though *BTNL9, LECT2* and ENSG00000267561 showed no change, as expected given that ClinVar does not contain any P/LP variants for these genes (except for CNVs which we do not consider in our study). The reduction in genotypes for the rest of genes resulted in a marked attenuation of P-values which, in most cases, resulted in a complete loss of signal (Fig. S11). To conclude, our association signals with *FLG, HBB, SERPINA1, MUTYH, PYGM,* and *ODAD1*, several of which are novel, can be attributed to established monogenic alleles whose pleiotropic effects extend to the complex traits analysed here.

Note S9. Supplemental Acknowledgements

Genes & Health is/has recently been core-funded by Wellcome (WT102627, WT210561), the Medical Research Council (UK) (M009017, MR/X009777/1, MR/X009920/1), Higher Education Funding Council for England Catalyst, Barts Charity (845/1796), Health Data Research UK (for London substantive site), and research delivery support from the NHS National Institute for Health Research Clinical Research Network (North Thames). We acknowledge the support of the National Institute for Health and Care Research Barts Biomedical Research Centre (NIHR203330); a delivery partnership of Barts Health NHS Trust, Queen Mary University of London, St George's University Hospitals NHS Foundation Trust and St George's University of London Genes & Health is/has recently been funded by Alnylam Pharmaceuticals, Genomics PLC; and a Life Sciences Industry Consortium of AstraZeneca PLC, Bristol-Myers Squibb Company, GlaxoSmithKline Research and Development Limited, Maze Therapeutics Inc, Merck Sharp & Dohme LLC, Novo Nordisk A/S, Pfizer Inc, Takeda Development Centre Americas Inc. We thank Social Action for Health, Centre of The Cell, members of our Community Advisory Group, and staff who have recruited and collected data from volunteers. We thank the NIHR National Biosample Centre (UK Biocentre), the Social Genetic & Developmental Psychiatry Centre (King's College London), Wellcome Sanger Institute, and Broad Institute for sample processing, genotyping, sequencing and variant annotation. This work uses data provided by patients and collected by the NHS as part of their care and support. This research utilised Queen Mary University of London's Apocrita HPC facility, supported by QMUL Research-IT, http://doi.org/10.5281/zenodo.438045. We thank: Barts Health NHS Trust, NHS Clinical Commissioning Groups (City and Hackney, Waltham Forest, Tower Hamlets, Newham, Redbridge, Havering, Barking and Dagenham), East London NHS Foundation Trust, Bradford Teaching Hospitals NHS Foundation Trust, Public Health England (especially David Wyllie), Discovery Data Service/Endeavour Health Charitable Trust (especially David Stables), Voror Health Technologies Ltd (especially Sophie Don), NHS England (for what was NHS Digital) - for GDPR-compliant data sharing backed by individual written informed consent. Most of all we thank all of the volunteers participating in Genes & Health.

This research was made possible through access to data in the National Genomic Research Library, which is managed by Genomics England Limited (a wholly owned company of the Department of Health and Social Care). The National Genomic Research Library holds data provided by patients and collected by the NHS as part of their care and data collected as part of their participation in research. The National Genomic Research Library is funded by

the National Institute for Health Research and NHS England. The Wellcome Trust, Cancer Research UK and the Medical Research Council have also funded research infrastructure.

We gratefully acknowledge *All of Us* participants for their contributions, without whom this research would not have been possible. We also thank the National Institutes of Health's All of Us Research Program for making available the participant data [and/or samples and/or cohort] examined in this study. The *All of Us* Research Program is supported by the National Institutes of Health, Office of the Director: Regional Medical Centers: 1 OT2 OD026549; 1 OT2 OD026554; 1 OT2 OD026557; 1 OT2 OD026556; 1 OT2 OD026550; 1 OT2 OD 026552; 1 OT2 OD026553; 1 OT2 OD026548; 1 OT2 OD026551; 1 OT2 OD026555; IAA #: AOD 16037; Federally Qualified Health Centers: HHSN 263201600085U; Data and Research Center: 5 U2C OD023196; Biobank: 1 U24 OD023121; The Participant Center: U24 OD023176; Participant Technology Systems Center: 1 U24 OD023163; Communications and Engagement: 3 OT2 OD023205; 3 OT2 OD023206; and Community Partners: 1 OT2 OD025277; 3 OT2 OD025315; 1 OT2 OD025337; 1 OT2 OD025276.

This work was supported in part by Google Cloud Research Credits provided by Google.

This work was supported in part through the computational and data resources and staff expertise provided by Scientific Computing and Data at the Icahn School of Medicine at Mount Sinai and supported by the Clinical and Translational Science Awards (CTSA) grant UL1TR004419 from the National Center for Advancing Translational Sciences. The Mount Sinai BioMe Biobank has been supported by The Andrea and Charles Bronfman Philanthropies and in part by Federal funds from the NHLBI and NHGRI (U01HG00638001; U01HG007417; X01HL134588). We thank all participants in the Mount Sinai BioMe Biobank. We also thank all of our recruiters who have assisted in data collection and management, and we are grateful for the computational resources and staff expertise provided by Scientific Computing at the Icahn School of Medicine at Mount Sinai.

We thank all participants of BioBank Japan. S.N. was supported by AMED (JP25tm0424228, JP24tm0524009) and Japan Foundation for Applied Enzymology. Y.O. was supported by JSPS KAKENHI (25H01057), and AMED (JP24km0405217, JP24ek0109594, JP24ek0410113, JP24kk0305022, JP243fa627002, JP243fa627010, JP243fa627011, JP24zf0127008, JP24tm0524002, JP24wm0625504, JP24gm1810011), JST Moonshot R&D (JPMJMS2021, JPMJMS2024), Takeda Science Foundation, Ono Pharmaceutical Foundation for Oncology, Immunology, and Neurology, Bioinformatics Initiative of Osaka University Graduate School of Medicine, Institute for Open and Transdisciplinary Research

Initiatives, Center for Infectious Disease Education and Research (CiDER), and Center for Advanced Modality and DDS (CAMaD), Osaka University, RIKEN TRIP initiative (AGIS).
During the preparation of this work the authors used *ChatGPT* and *Claude* in order to simplify handling of results files and improve clarity in the manuscript. After using this tool/service, the authors reviewed and edited the content as needed and take full responsibility for the content of the published article.

Supplemental Figures

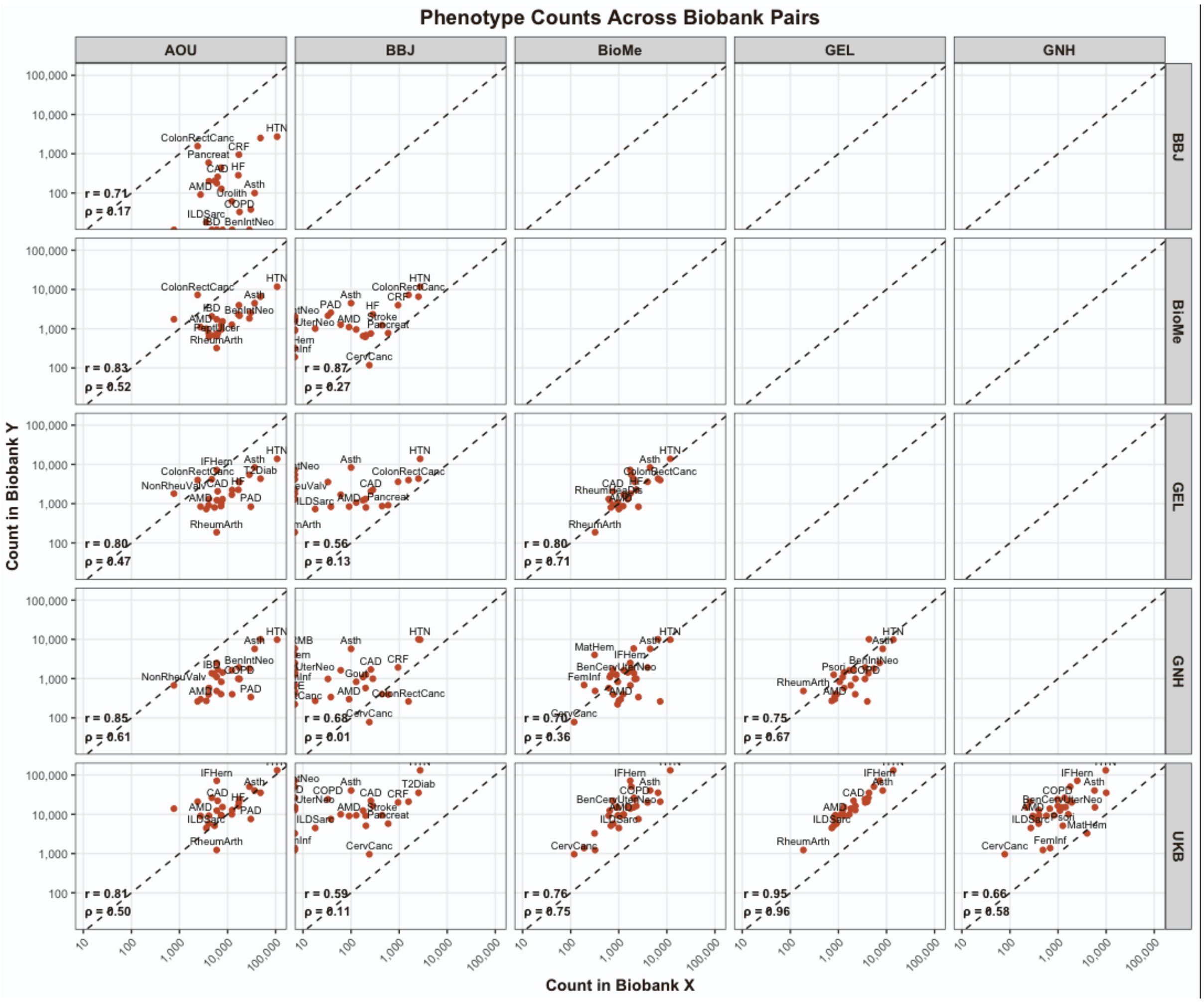

Figure S1: Scatter plot of the pairwise comparison of disease prevalence between biobanks.

The x-axis represents the prevalence of a specific trait in one biobank (labelled along the top), while the y-axis represents the prevalence of the same trait in another biobank (labelled on the right hand side). Each point corresponds to a specific trait aggregated across all ancestry groups. The diagonal dashed line indicates where the prevalence would be equal across biobanks. Pearson (r) and Spearman (ρ) correlation coefficients are shown in each panel, quantifying the strength and direction of the linear and rank-based relationships, respectively. The red dashed line indicates x = y. Please refer to Sup. Table 1 for the complete list of name abbreviations.

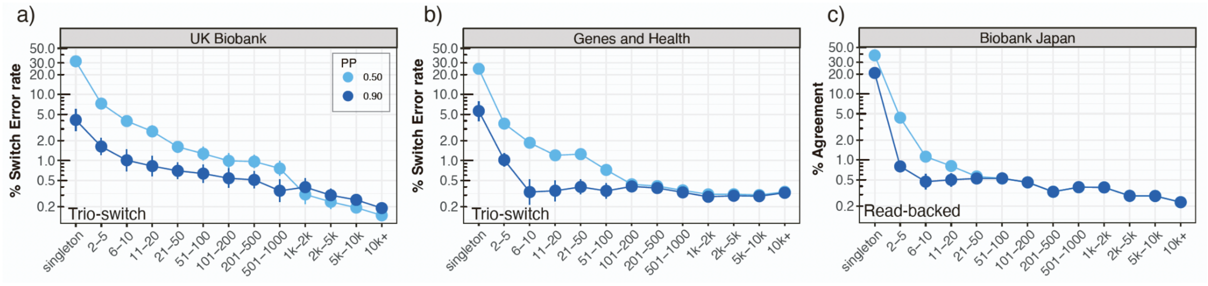

Figure S2: Assessment of phasing accuracy through trio-switch error rates and read-backed phasing.

Phasing accuracy assessment for three biobanks: The *x*-axis represents the MAC bin for the variants, while the *y*-axis shows phasing accuracy, measured by switch errors (a-b) or agreement between read-backed and statistically inferred haplotypes (c). Phasing accuracy is shown before (PP ≥ 0.5) and after (PP ≥ 0.9) filtering to confidently phased variants. Confidence intervals (CIs) indicate 95% binomial confidence intervals. All biobanks used pLoF variants, except for BBJ, which used pLoF plus damaging missense/protein-altering variants due to insufficient CH pLoF variants. Refer to Sup. Table 11 for the underlying data.

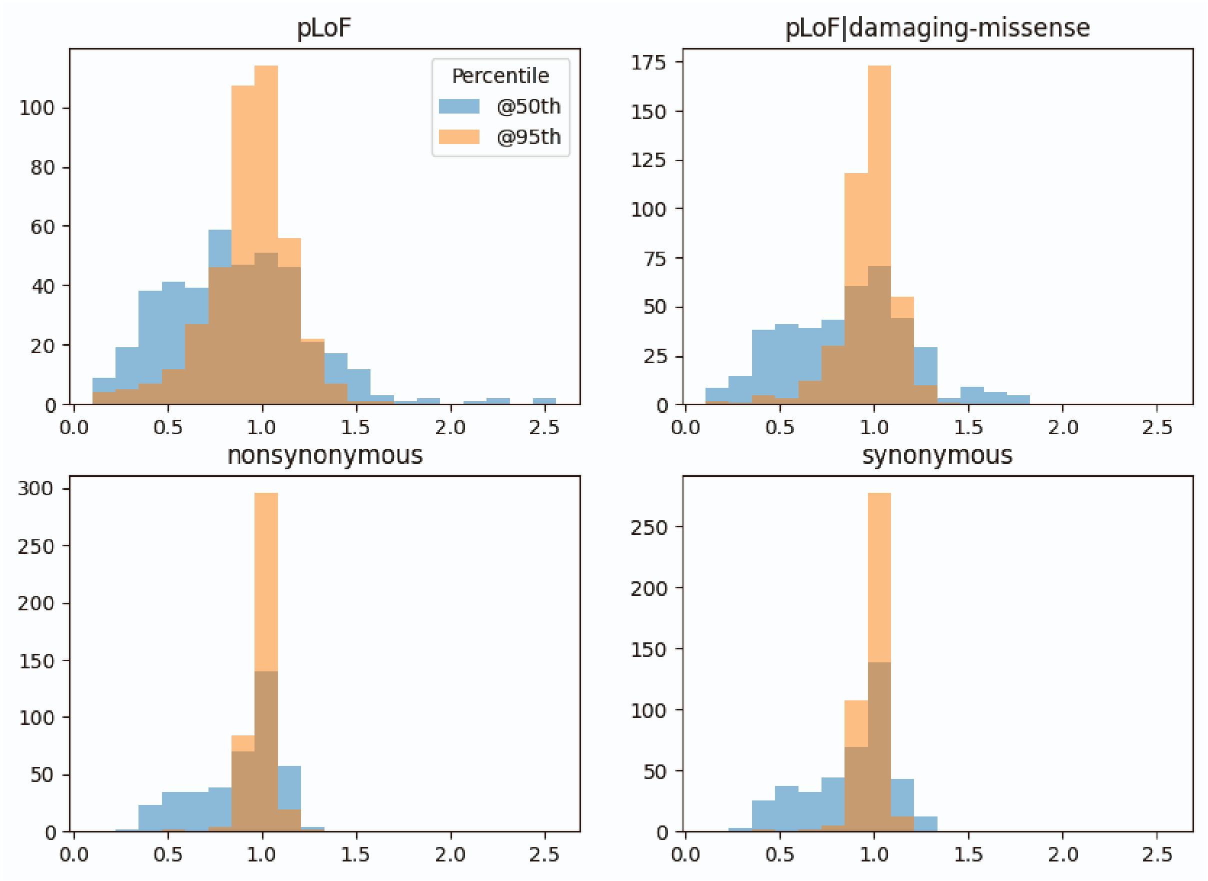

Figure S3: Distribution of inflation factors for recessive tests across all cohorts and phenotypes under consideration.

Histograms showing a comparison of the distribution of the λ_{GC} inflation factors (calculated at the median statistic) with that of λ_{95} (calculated at the 95th percentile) for all recessive tests and each annotation we consider. We note that the maximum values for each annotation are as follows (50th vs 95th): 2.57 vs 1.60 (pLoF), 1.81 vs 1.42 (pLoF|damaging-missense, 1.32 vs 1.27 (nonsynonymous), and 1.30 vs 1.174 (synonymous).

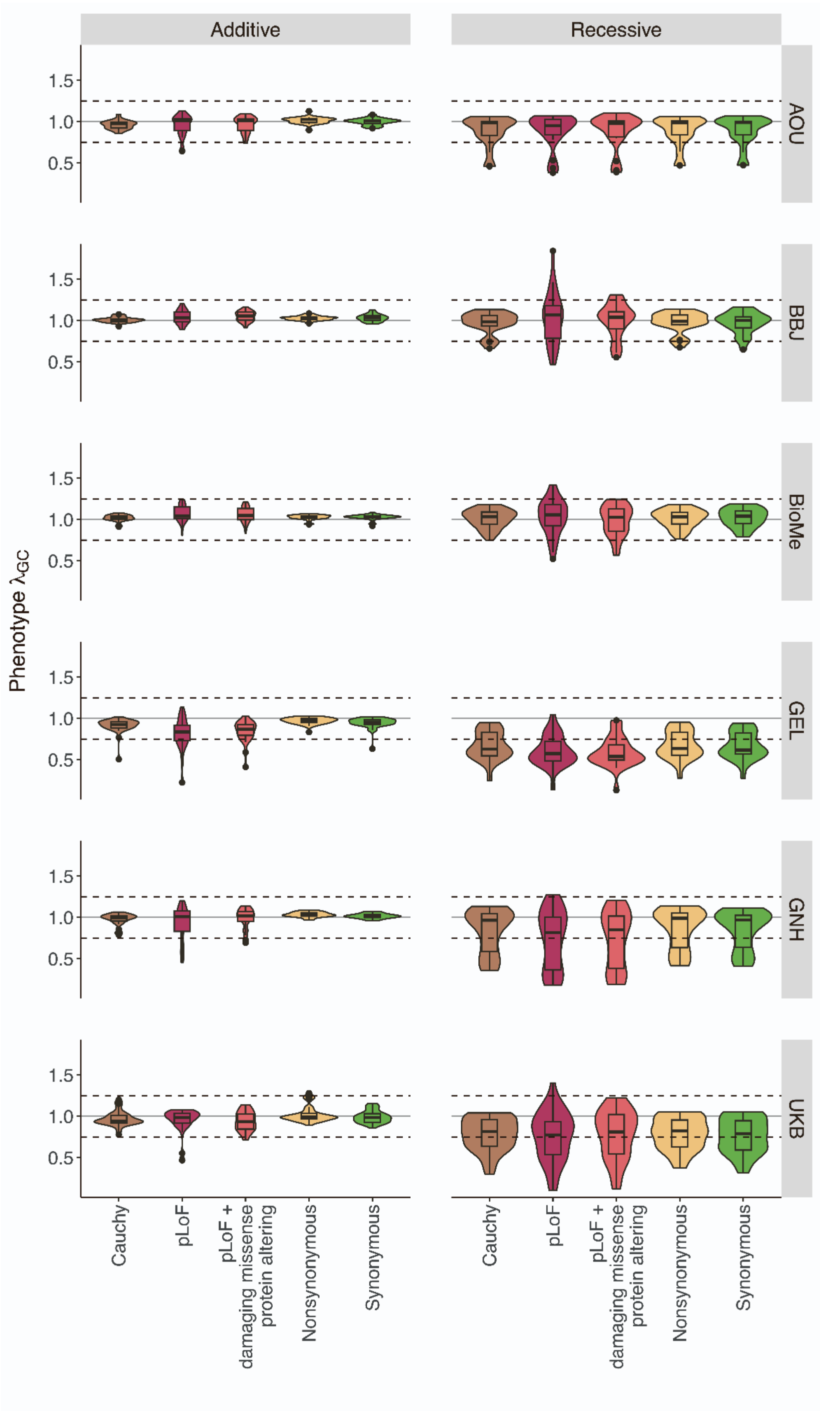

Figure S4: Comparison of test-statistic inflation across biobanks and annotation categories before meta-analysis using λ_{GC}

Violin plots with overlaid boxplots showing the distribution of inflation factors (λ_{GC}) across phenotypes on the y-axis. X-axis represents different variant annotation masks, including Cauchy-combined *P*-values. Dashed lines indicate $\lambda = 1.25$ and $\lambda = 0.75$ cutoffs. Violin shape depicts probability density; boxplot shows median, quartiles (Q1, Q3 as hinges), and whiskers extending to Q1−1.5×interquartile range (IQR) and Q3+1.5×IQR. Outliers beyond whiskers are plotted individually.

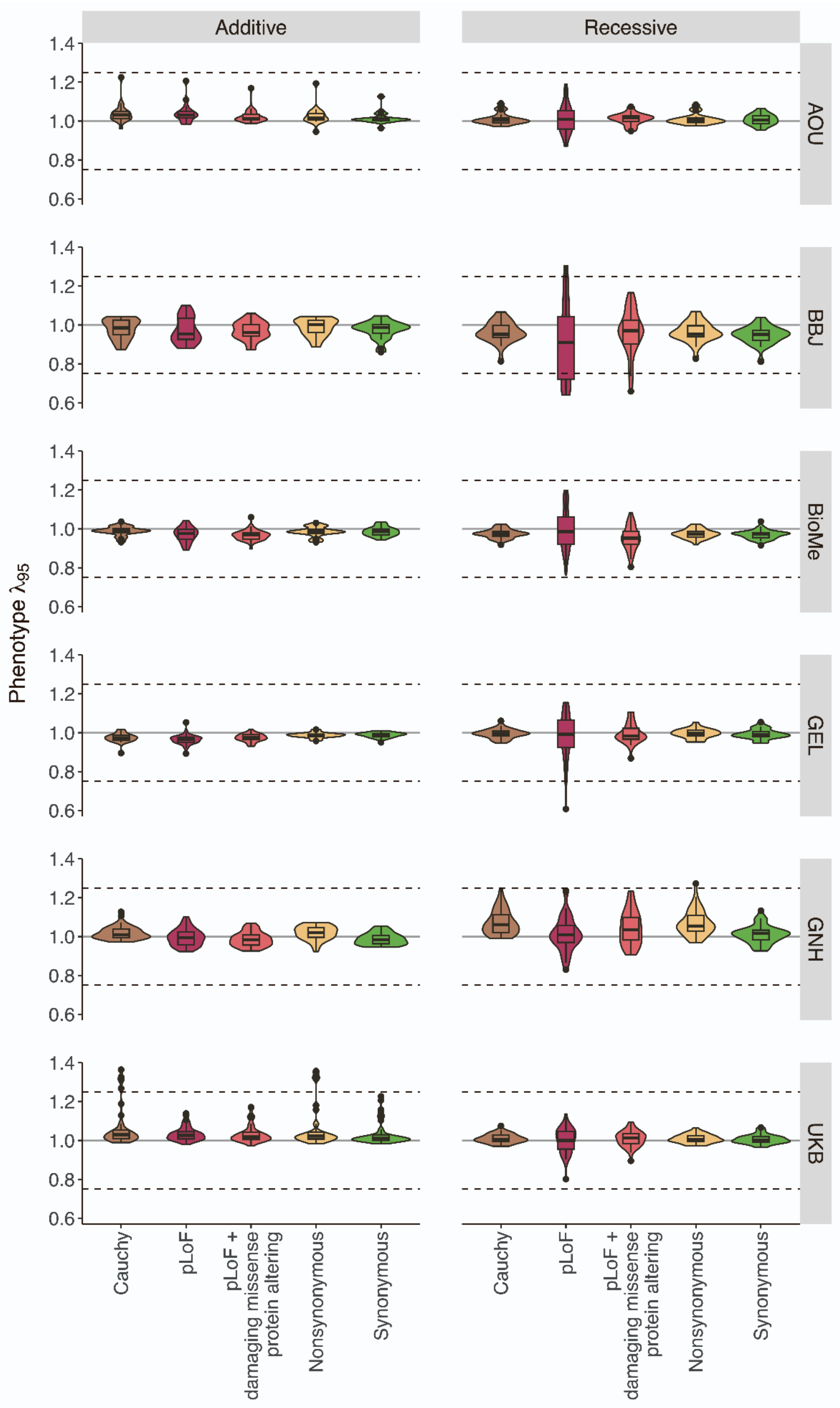

Figure S5: Comparison of test-statistic inflation across biobanks and annotation categories before meta-analysis using λ_{95}.

Violin plots with overlaid boxplots showing the distribution of inflation (λ_{95}, lambda at 95th percentile instead of 50th percentile) on the y-axis. X-axis represents different variant annotation masks, including Cauchy-combined *P*-values. Dashed lines indicate λ=1.25 and λ=0.75 cutoffs. Violin shape depicts probability density; boxplot shows median, quartiles (Q1, Q3 as hinges), and whiskers extending to Q1−1.5×IQR and Q3+1.5×IQR. Outliers beyond whiskers are plotted individually.

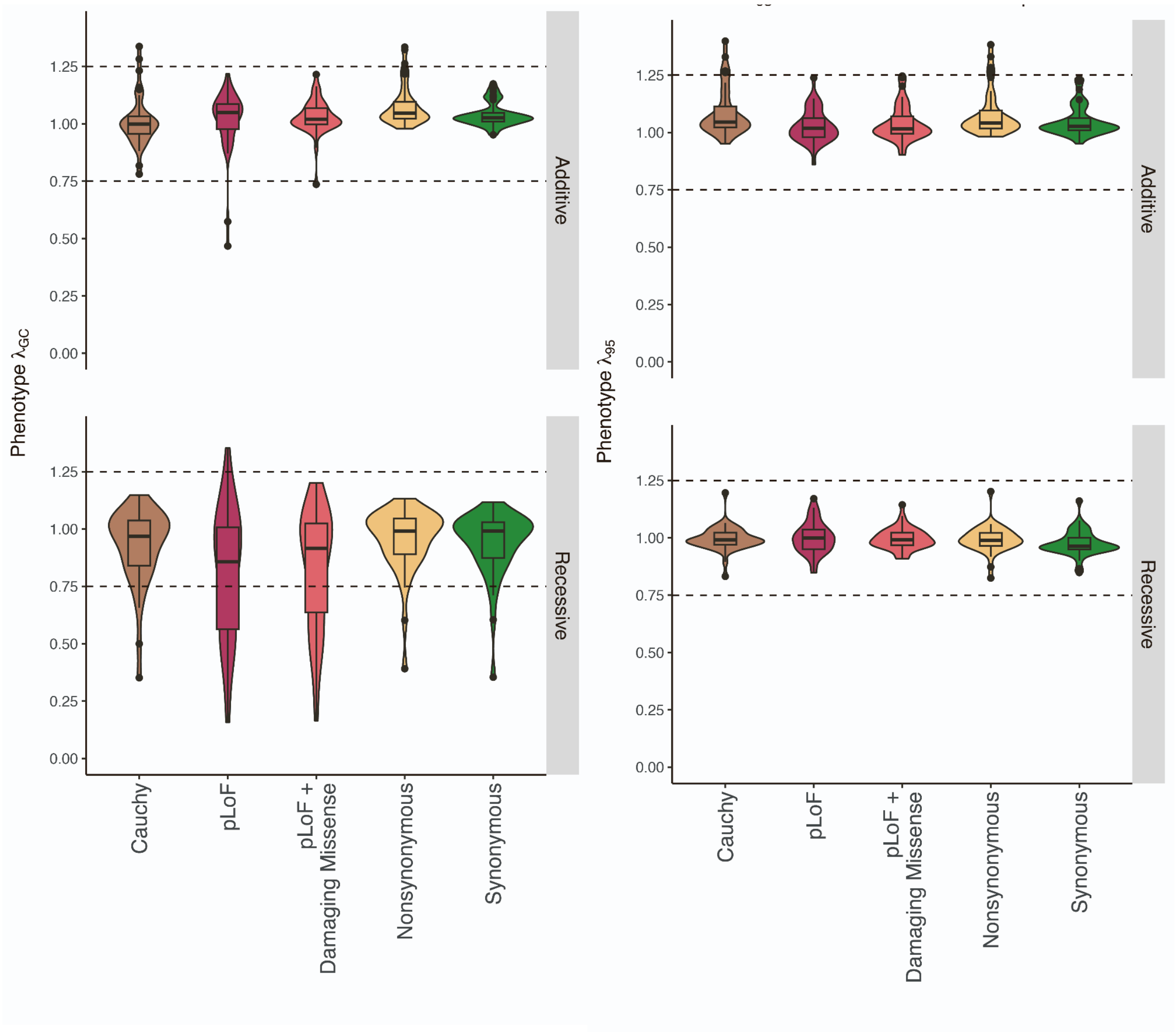

Figure S6: λ_{GC} and λ_{95} for additive and recessive meta-analyses by variant annotation masks.

Violin plots with overlaid boxplots showing the distribution of inflation factors across phenotypes for additive, recessive and nonadditive analyses. Specifically, we consider (**left**) λ_{GC} (genomic control lambda) and (**right**) λ_{95} (lambda estimated at the 95th percentile instead of 50th percentile). The x-axis represents different variant annotation masks, including combinations of *P*-values using the Cauchy distribution. Dashed lines indicate the λ=1.25 and λ=0.75 cutoffs on the y-axis. The violin plot's outer shape depicts the probability density, while the boxplot visualizes the median, quartiles (Q1 and Q3 as hinges), and whiskers extending to Q1−1.5×IQR and Q3+1.5×IQR. Data points beyond the whiskers (outliers) are plotted individually.

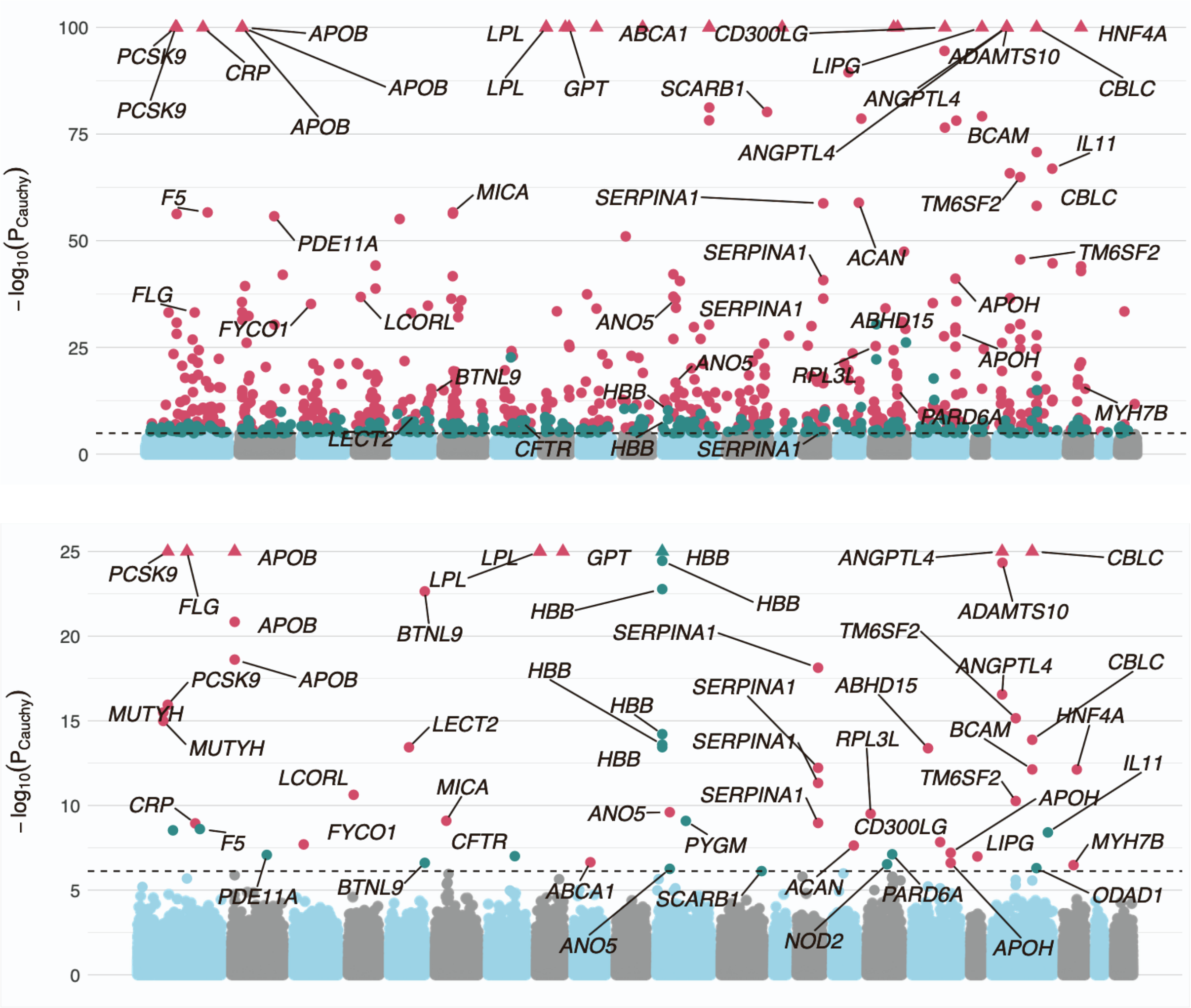

Figure S7: Manhattan plot for additive and recessive meta-analysis.

Manhattan plot for additive and recessive meta-analysis across six biobanks. The meta-analysis was performed first across four different variant annotation masks, followed by a combination of *P*-values using the Cauchy distribution, which are plotted here on the Y-axis. Each point represents a gene-trait association. Points are colored red if the gene-trait associations have been deemed significant (FDR< 0.01) in an analysis of Europeans in UKBB alone, and are otherwise colored green. To aid visualisation, *P*-values are truncated in the additive and recessive facets. Such truncated *P*-values are denoted by a triangle, and non-truncated *P*-values are circles.

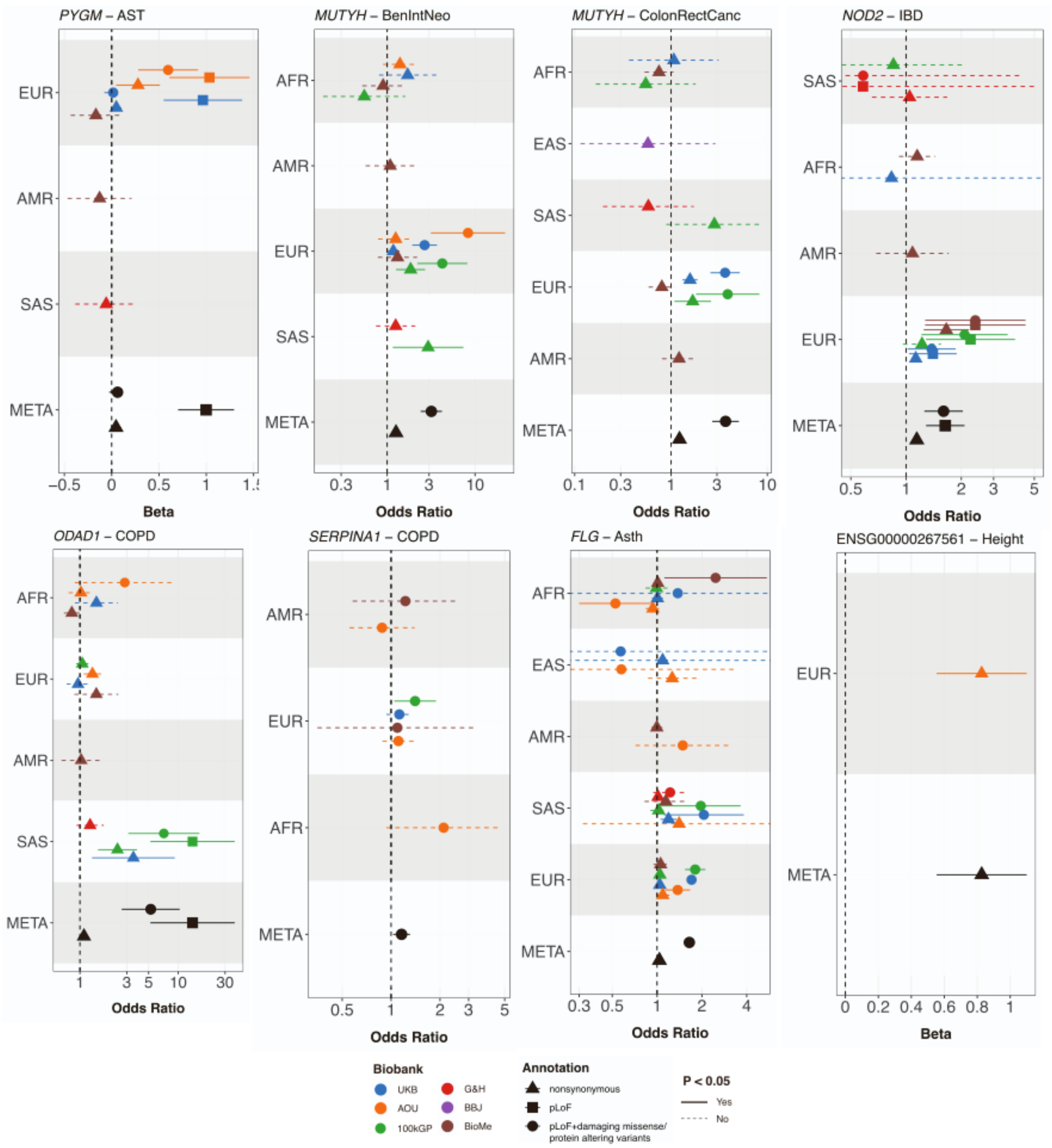

Figure S8: Forest plot of putatively recessive gene-trait associations detected by meta-analysis.

Forest plot of effect sizes or ORs, for quantitative and binary traits respectively, and 95% CIs across significant recessive gene-trait associations (FDR<1% corresponding to $p_{rec} < 7.5 \times 10^{-7}$), in addition to those presented in (main) Figure 5. The plot includes: 1) pLoF variants (squares); 2) pLoF|damaging_missense (circles); and 3) nonsynonymous (triangles), stratified by ancestry, with different biobanks indicated by different colors. We show only associations that we were able to test due to having at least five bi-allelic individuals, and with signals observed in at least two cohorts. To aid visualization, large CIs have been truncated, and dotted lines indicate associations with $p_{rec} > 0.05$. AST: Aspartate aminotransferase; Asth: asthma; please refer to Sup. Table 1 for the complete list of phenotype abbreviations.

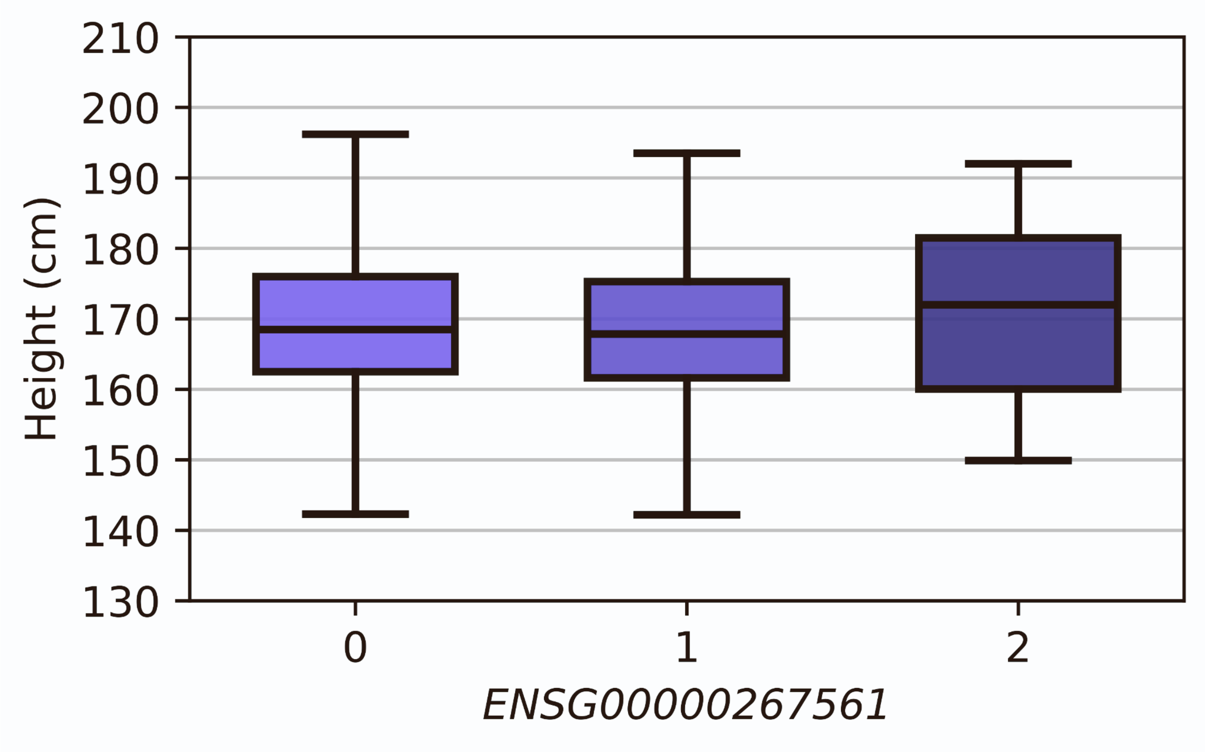

Figure S9: Box plot showing distribution of height (cm) stratified by genotype at ENSG00000267561.

Plot based on nonsynonymous variant burden in AOU:EUR, as that was the only cohort with sufficient number of individuals for association testing (N<20). Individuals are grouped by genotype class: 0 = reference homozygotes, 1 = heterozygotes, and 2 = bi-allelic individuals (homozygous or compound heterozygous). We note that outliers are not shown to comply with privacy rules in AOU.

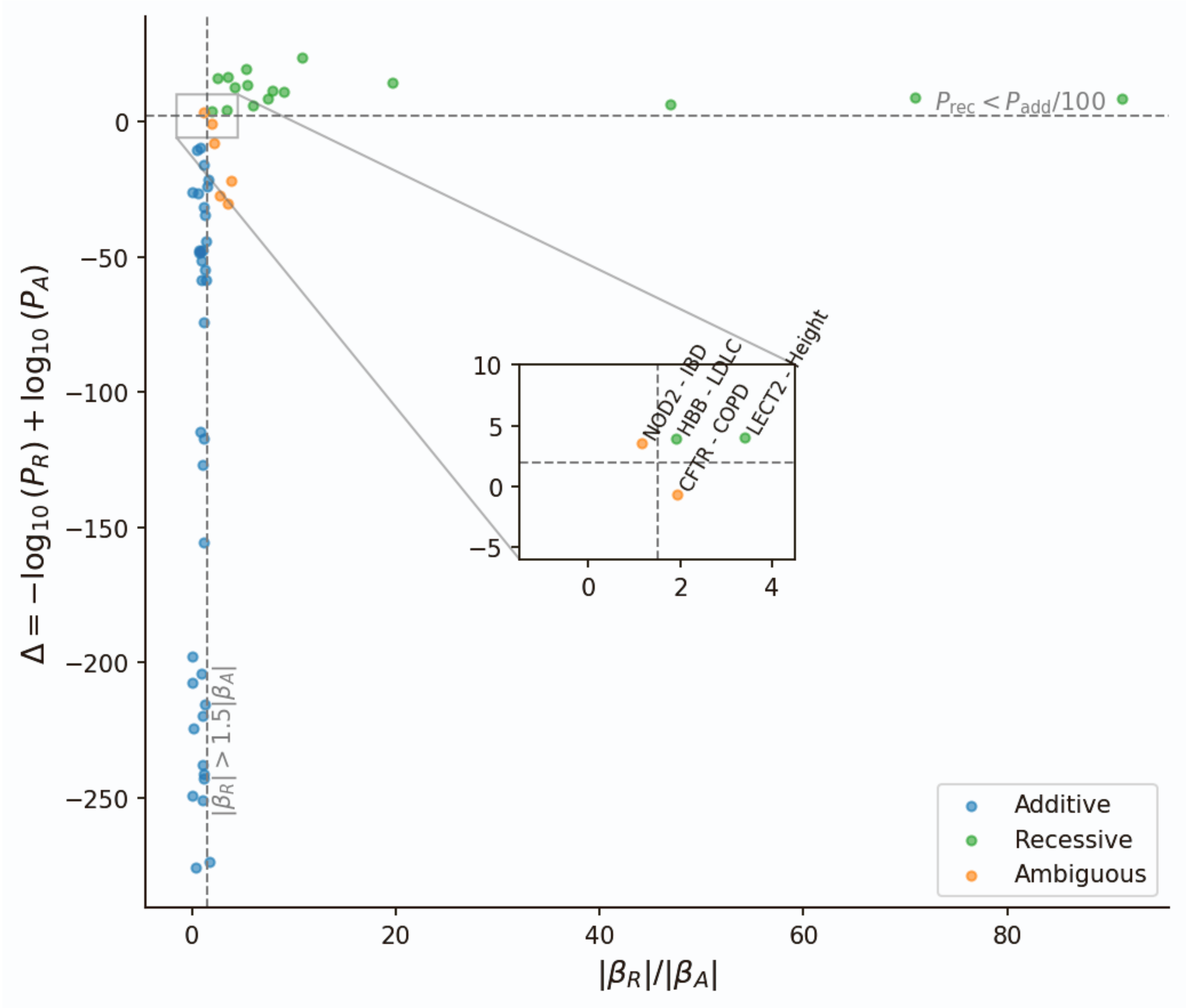

Figure S10: Assessing recessive versus additive architecture

Scatterplot comparing two indicators of inheritance mode, for each significant gene-trait association in our meta-analysis (FDR < 1%), after the Cauchy combination test. On the x-axis we show the absolute recessive effect ($|\beta_R|$) divided by absolute additive effect ($|\beta_A|$) with the vertical dashed line marking an indicative threshold of 1.5 as discussed in Sup. Note 7. On the y-axis we show the difference between $\log_{10}P_A$ and $\log_{10}P_R$, with the horizontal line indicating the threshold of two orders used in our main analysis. Points are coloured by this alternative classification, whereby green = recessive ($\Delta>0$ and $|\beta_R|>1.5|\beta_A|$), blue = additive ($\Delta<0$ and $|\beta_R| < 1.5|\beta_A|$), and orange = ambiguous (do not meet either pair of criteria). The inset enlarges the dense region near the origin and labels the four loci that lie close to the decision boundaries.

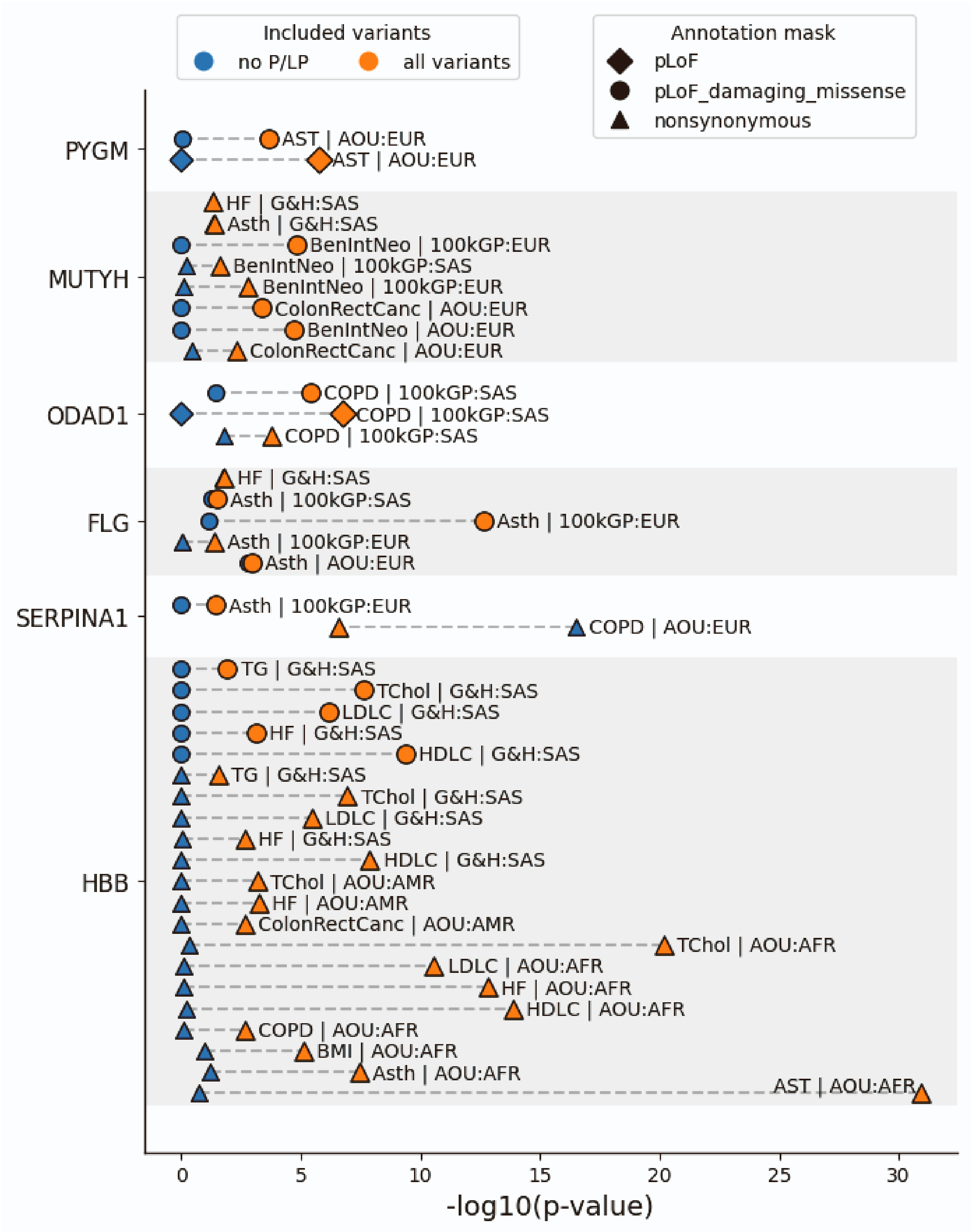

Figure S11: Forest plot summarising the attenuation in association signal after excluding P/LP variants.

We compare the $\log_{10}$ P-values for association testing when using all variants (orange) to those after dropping Pathogenic (P) or Likely-Pathogenic (LP) in ClinVar[31] (blue), stratified by annotation mask and considering genes which are identified with recessive associations in our main analysis. To aid interpretation, a *p*-value of 1.00 is used for cases with < 5 bi-allelic individuals after dropping P/LP variants.

Supplemental References

1. Chen, C.-Y., Chen, T.-T., Anne Feng, Y.-C., Yu, M., Lin, S.-C., Longchamps, R.J., Wang, S.-H., Hsu, Y.-H., Yang, H.-I., Kuo, P.-H., et al. (2024). Analysis across Taiwan Biobank, Biobank Japan, and UK Biobank identifies hundreds of novel loci for 36 quantitative traits. Cell Genom. *4*, 100640. https://doi.org/10.1016/j.xgen.2024.100640.

2. Chen, L., Magliano, D.J., and Zimmet, P.Z. (2011). The worldwide epidemiology of type 2 diabetes mellitus--present and future perspectives. Nat. Rev. Endocrinol. *8*, 228–236. https://doi.org/10.1038/nrendo.2011.183.

3. Gujral, U.P., and Kanaya, A.M. (2021). Epidemiology of diabetes among South Asians in the United States: lessons from the MASALA study. Ann. N. Y. Acad. Sci. *1495*, 24–39. https://doi.org/10.1111/nyas.14530.

4. Carter, P., Gray, L.J., Morris, D.H., Davies, M.J., and Khunti, K. (2013). South Asian individuals at high risk of type 2 diabetes have lower plasma vitamin C levels than white Europeans. J. Nutr. Sci. *2*, e21. https://doi.org/10.1017/jns.2013.15.

5. Bradley, D., and Hsueh, W. (2016). Type 2 diabetes in the elderly: Challenges in a unique patient population. J. Geriatr. Med. Gerontol. *2*. https://doi.org/10.23937/2469-5858/1510014.

6. Dendup, T., Feng, X., Clingan, S., and Astell-Burt, T. (2018). Environmental risk factors for developing type 2 diabetes mellitus: A systematic review. Int. J. Environ. Res. Public Health *15*. https://doi.org/10.3390/ijerph15010078.

7. Gaskin, D.J., Thorpe, R.J., Jr, McGinty, E.E., Bower, K., Rohde, C., Young, J.H., LaVeist, T.A., and Dubay, L. (2014). Disparities in diabetes: the nexus of race, poverty, and place. Am. J. Public Health *104*, 2147–2155. https://doi.org/10.2105/AJPH.2013.301420.

8. GBD Results Institute for Health Metrics and Evaluation. https://vizhub.healthdata.org/gbd-results/.

9. Caulfield, M., Davies, J., Dennys, M., Elbahy, L., Fowler, T., Hill, S., Hubbard, T., Jostins, L., Maltby, N., Mahon-Pearson, J., et al. (2020). National Genomic Research Library. (figshare). https://doi.org/10.6084/M9.FIGSHARE.4530893.V7 https://doi.org/10.6084/M9.FIGSHARE.4530893.V7.

10. Sosinsky, A., Ambrose, J., Cross, W., Turnbull, C., Henderson, S., Jones, L., Hamblin, A., Arumugam, P., Chan, G., Chubb, D., et al. (2024). Insights for precision oncology from the integration of genomic and clinical data of 13,880 tumors from the 100,000 Genomes Cancer Programme. Nat. Med. *30*, 279–289. https://doi.org/10.1038/s41591-023-02682-0.

11. Shi, S., Rubinacci, S., Hu, S., Moutsianas, L., Stuckey, A., Need, A.C., Palamara, P.F., Caulfield, M., Marchini, J., and Myers, S. (2024). A Genomics England haplotype reference panel and imputation of UK Biobank. Nat. Genet. *56*, 1800–1803. https://doi.org/10.1038/s41588-024-01868-7.

12. Karczewski, K.J., Francioli, L.C., Tiao, G., Cummings, B.B., Alföldi, J., Wang, Q., Collins, R.L., Laricchia, K.M., Ganna, A., Birnbaum, D.P., et al. (2020). The mutational constraint spectrum quantified from variation in 141,456 humans. Nature *581*, 434–443. https://doi.org/10.1038/s41586-020-2308-7.

13. Lim, E.T., Raychaudhuri, S., Sanders, S.J., Stevens, C., Sabo, A., MacArthur, D.G., Neale, B.M., Kirby, A., Ruderfer, D.M., Fromer, M., et al. (2013). Rare complete knockouts in humans: population distribution and significant role in autism spectrum disorders. Neuron *77*, 235–242. https://doi.org/10.1016/j.neuron.2012.12.029.

14. Martin, M., Patterson, M., Garg, S., O Fischer, S., Pisanti, N., Klau, G.W., Schöenhuth, A., and Marschall, T. (2016). WhatsHap: fast and accurate read-based phasing. bioRxiv. https://doi.org/10.1101/085050.

15. Sulem, P., Helgason, H., Oddson, A., Stefansson, H., Gudjonsson, S.A., Zink, F., Hjartarson, E., Sigurdsson, G.T., Jonasdottir, A., Jonasdottir, A., et al. (2015). Identification of a large set of rare complete human knockouts. Nat. Genet. *47*, 448–452. https://doi.org/10.1038/ng.3243.

16. Narasimhan, V.M., Hunt, K.A., Mason, D., Baker, C.L., Karczewski, K.J., Barnes, M.R., Barnett, A.H., Bates, C., Bellary, S., Bockett, N.A., et al. (2016). Health and population effects of rare gene knockouts in adult humans with related parents. Science *352*, 474–477. https://doi.org/10.1126/science.aac8624.

17. Saleheen, D., Natarajan, P., Armean, I.M., Zhao, W., Rasheed, A., Khetarpal, S.A., Won, H.-H., Karczewski, K.J., O'Donnell-Luria, A.H., Samocha, K.E., et al. (2017). Human knockouts and phenotypic analysis in a cohort with a high rate of consanguinity. Nature *544*, 235–239. https://doi.org/10.1038/nature22034.

18. Oddsson, A., Sulem, P., Sveinbjornsson, G., Arnadottir, G.A., Steinthorsdottir, V., Halldorsson, G.H., Atlason, B.A., Oskarsson, G.R., Helgason, H., Nielsen, H.S., et al. (2023). Deficit of homozygosity among 1.52 million individuals and genetic causes of recessive lethality. Nat. Commun. *14*, 3453. https://doi.org/10.1038/s41467-023-38951-2.

19. Sun, K.Y., Bai, X., Chen, S., Bao, S., Zhang, C., Kapoor, M., Backman, J., Joseph, T., Maxwell, E., Mitra, G., et al. (2024). A deep catalogue of protein-coding variation in 983,578 individuals. Nature. https://doi.org/10.1038/s41586-024-07556-0.

20. Malawsky, D.S., van Walree, E., Jacobs, B.M., Heng, T.H., Huang, Q.Q., Sabir, A.H., Rahman, S., Sharif, S.M., Khan, A., Mirkov, M.U., et al. (2023). Influence of autozygosity on common disease risk across the phenotypic spectrum. medRxiv. https://doi.org/10.1101/2023.02.01.23285346.

21. Jurgens, S.J., Wang, X., Choi, S.H., Weng, L.-C., Koyama, S., Pirruccello, J.P., Nguyen, T., Smadbeck, P., Jang, D., Chaffin, M., et al. (2024). Rare coding variant analysis for human diseases across biobanks and ancestries. Nat. Genet. *56*, 1811–1820. https://doi.org/10.1038/s41588-024-01894-5.

22. Yang, J., Weedon, M.N., Purcell, S., Lettre, G., Estrada, K., Willer, C.J., Smith, A.V., Ingelsson, E., O'Connell, J.R., Mangino, M., et al. (2011). Genomic inflation factors under polygenic inheritance. Eur. J. Hum. Genet. *19*, 807–812. https://doi.org/10.1038/ejhg.2011.39.

23. Sohail, M., Maier, R.M., Ganna, A., Bloemendal, A., Martin, A.R., Turchin, M.C., Chiang, C.W., Hirschhorn, J., Daly, M.J., Patterson, N., et al. (2019). Polygenic adaptation on height is overestimated due to uncorrected stratification in genome-wide association studies. Elife *8*. https://doi.org/10.7554/eLife.39702.

24. Clark, D.W., Okada, Y., Moore, K.H.S., Mason, D., Pirastu, N., Gandin, I., Mattsson, H., Barnes, C.L.K., Lin, K., Zhao, J.H., et al. (2019). Associations of autozygosity with a broad range of human phenotypes. Nat. Commun. *10*, 4957. https://doi.org/10.1038/s41467-019-12283-6.

25. Karczewski, K.J., Solomonson, M., Chao, K.R., Goodrich, J.K., Tiao, G., Lu, W., Riley-Gillis, B.M., Tsai, E.A., Kim, H.I., Zheng, X., et al. (2022). Systematic single-variant and gene-based association testing of thousands of phenotypes in 394,841 UK Biobank exomes. Cell Genom. *2*, 100168. https://doi.org/10.1016/j.xgen.2022.100168.

26. Onoufriadis, A., Paff, T., Antony, D., Shoemark, A., Micha, D., Kuyt, B., Schmidts, M., Petridi, S., Dankert-Roelse, J.E., Haarman, E.G., et al. (2013). Splice-site mutations in the axonemal outer dynein arm docking complex gene CCDC114 cause primary ciliary dyskinesia. Am. J. Hum. Genet. *92*, 88–98. https://doi.org/10.1016/j.ajhg.2012.11.002.

27. Knowles, M.R., Leigh, M.W., Ostrowski, L.E., Huang, L., Carson, J.L., Hazucha, M.J., Yin, W., Berg, J.S., Davis, S.D., Dell, S.D., et al. (2013). Genetic Disorders of Mucociliary Clearance Consortium, Exome sequencing identifies mutations in CCDC114 as a cause of primary ciliary dyskinesia. Am. J. Hum. Genet *92*, 99–106.

28. Guichard, C., Harricane, M.C., Lafitte, J.J., Godard, P., Zaegel, M., Tack, V., Lalau, G., and

Bouvagnet, P. (2001). Axonemal dynein intermediate-chain gene (DNAI1) mutations result in situs inversus and primary ciliary dyskinesia (Kartagener syndrome). Am. J. Hum. Genet. *68*, 1030–1035. https://doi.org/10.1086/319511.

29. Hannah, W.B., Derks, T.G.J., Drumm, M.L., Grünert, S.C., Kishnani, P.S., and Vissing, J. (2023). Glycogen storage diseases. Nat. Rev. Dis. Primers *9*.

30. Heyne, H.O., Karjalainen, J., Karczewski, K.J., Lemmelä, S.M., Zhou, W., FinnGen, Havulinna, A.S., Kurki, M., Rehm, H.L., Palotie, A., et al. (2023). Mono- and biallelic variant effects on disease at biobank scale. Nature *613*, 519–525. https://doi.org/10.1038/s41586-022-05420-7.

31. Landrum, M.J., Lee, J.M., Benson, M., Brown, G., Chao, C., Chitipiralla, S., Gu, B., Hart, J., Hoffman, D., Hoover, J., et al. (2016). ClinVar: public archive of interpretations of clinically relevant variants. Nucleic Acids Res. *44*, D862–D868. https://doi.org/10.1093/nar/gkv1222.
