# Supplementary material for: Meta-analysis across six global biobanks identifies recessive coding associations with complex traits and diseases
Source: Am J Hum Genet. 2026 May 1;113(6):1330–46. doi: 10.1016/j.ajhg.2026.04.005 (PMC13277689; doi:10.1016/j.ajhg.2026.04.005)
Supplement: Document S2. Article plus supplemental information [file mmc3.pdf]

ARTICLE

Meta-analysis across six global biobanks identifies recessive coding associations with complex traits and diseases

Graphical abstract

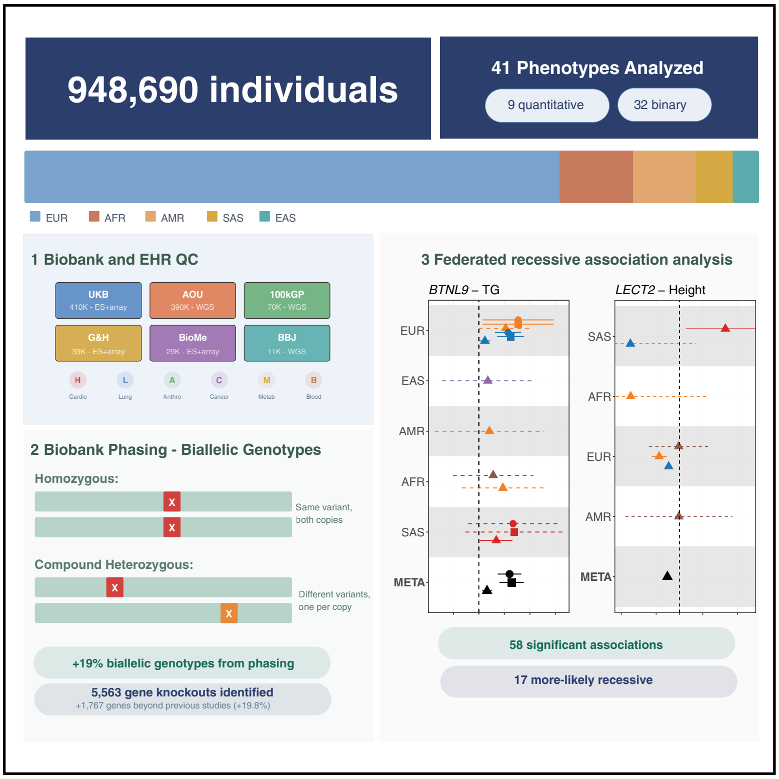

Authors

Frederik H. Lassen, Georgios Kalantzis, Andrea Eoli, ..., Henrike O. Heyne, Hilary C. Martin, Duncan S. Palmer

Correspondence

hcm@sanger.ac.uk (H.C.M.), duncan.palmer@stats.ox.ac.uk (D.S.P.)

We meta-analyze rare bi-allelic coding variation in approximately 1 million individuals across six biobanks, identifying 17 recessive gene-trait associations, including *HBB* with heart failure and *LECT2* with reduced height. Incorporating compound-heterozygous genotypes via statistical phasing increases the number of bi-allelic damaging genotypes by 19%, boosting discovery power.

Lassen et al., 2026, The American Journal of Human Genetics *113*, 1330–1346
June 4, 2026 © 2026 The Authors. Published by Elsevier Inc. on behalf of American Society of Human Genetics.
https://doi.org/10.1016/j.ajhg.2026.04.005

ARTICLE

Meta-analysis across six global biobanks identifies recessive coding associations with complex traits and diseases

Frederik H. Lassen,[1,19] Georgios Kalantzis,[2,19] Andrea Eoli,[3,4,5,20] Barney Hill,[1,20] Kyuto Sonehara,[2,6,20] Shinichi Namba,[6,20] Isaac Wade,[7,20] Sam Hodgson,[8] Wei Zhou,[9,10] BioBank Japan Project, Genes & Health Research Team, BRaVa Consortium, Benjamin M. Neale,[10,11,12] Konrad J. Karczewski,[11,12] Yukinori Okada,[6,13,14] David A. van Heel,[15] Sarah Finer,[8] Cecilia M. Lindgren,[7,16,17] Henrike O. Heyne,[3,4,5,11] Hilary C. Martin,[2,21,*] and Duncan S. Palmer[11,17,18,21,*]

Summary

Rare bi-allelic variation is a major contributor to human disease risk, yet its effects are difficult to study at scale in population cohorts owing to the limited number of individuals with putatively deleterious bi-allelic genotypes and the challenges of accurately phasing low-frequency variants. Here, we present recessive, gene-based analyses of rare and low-frequency variants in up to 948,690 exome- or whole-genome-sequenced individuals across six biobanks with linked electronic health records. Through statistical phasing, we inferred putatively damaging compound-heterozygous genotypes, increasing the number of bi-allelic damaging genotypes by 19%. Restricting to predicted loss-of-function (pLoF) variants, we identified 5,563 genes harboring bi-allelic genotypes, a 19.8% increase in putative knockouts. We then considered all low-frequency variants (minor allele frequency [MAF] <5%) and performed gene-based recessive association testing using putatively damaging bi-allelic genotypes, identifying 58 significant associations (false discovery rate [FDR] ≤1% or $p_{\text{rec}} \leq 7.5 \times 10^{-7}$) after meta-analysis and Cauchy combination of nonsynonymous annotations. Comparing recessive and additive models, we found 17 instances where recessive effects were more pronounced, including several previously unreported associations, such as *HBB* with heart failure ($p_{\text{rec}} = 2.6 \times 10^{-14}$; $p_{\text{add}} = 0.98$), *LECT2* with height ($p_{\text{rec}} = 3.7 \times 10^{-14}$; $p_{\text{add}} = 4.1 \times 10^{-10}$), and ENSG00000267561 with height ($p_{\text{rec}} = 2.9 \times 10^{-9}$; $p_{\text{add}} = 0.37$). This study demonstrates the potential of federated approaches to study the effects of rare bi-allelic variation.

Introduction

Large sample sizes are required to discover robust associations between rare or low-frequency genetic variants and complex traits. This is particularly true for recessive associations, as their detection requires perturbation of both gene copies, either through homozygous or compound-heterozygous (CH) variants. For homozygotes, in the absence of autozygosity, statistical power scales with the square of minor allele frequency (MAF) of variants, making recessive effects more difficult to detect than additive effects. Detecting associations driven by CH variants is even more challenging, as they must first be accurately phased and their expected frequency scales with the sum of the products of MAFs across all qualifying variant pairs within a gene. Consequently, traditional association studies have been underpowered to detect recessive contributions to complex traits. To this end, in this study, we have brought together genetic and phenotypic data from multiple biobanks, comprising approximately 950,000 individuals across five broad genetic-ancestry groups, enhancing our statistical power to detect rare recessive contributions to complex traits.

Individuals carrying bi-allelic loss-of-function (LoF) variants, which render both copies of a gene incapable of producing functional protein, have been called "human knockouts." These variants are of great interest to the scientific community, particularly for drug development, as

[1]Centre for Human Genetics, University of Oxford, Oxford, UK; [2]Wellcome Sanger Institute, Hinxton, UK; [3]Hasso Plattner Institute, Digital Engineering Faculty, University of Potsdam, Potsdam, Germany; [4]Windreich Department of Artificial Intelligence & Human Health, Icahn School of Medicine at Mount Sinai, New York, NY, USA; [5]Hasso Plattner Institute for Digital Health at Mount Sinai, Icahn School of Medicine at Mount Sinai, New York, NY, USA; [6]Department of Genome Informatics, Graduate School of Medicine, The University of Tokyo, Tokyo, Japan; [7]Nuffield Department of Population Health, Medical Sciences Division, University of Oxford, Oxford, UK; [8]Wolfson Institute of Population Health, Queen Mary University of London, London, UK; [9]Center for Genomic Medicine, Massachusetts General Hospital, Boston, MA, USA; [10]Stanley Center for Psychiatric Research, Broad Institute of MIT and Harvard, Cambridge, MA, USA; [11]Program in Medical and Population Genetics, Broad Institute of MIT and Harvard, Cambridge, MA, USA; [12]Novo Nordisk Foundation Center for Genomic Mechanisms of Disease, Broad Institute of MIT and Harvard, Cambridge, MA, USA; [13]Department of Statistical Genetics, Osaka University Graduate School of Medicine, Osaka, Japan; [14]Laboratory for Systems Genetics, RIKEN Center for Integrative Medical Sciences, Yokohama, Japan; [15]Blizard Institute, Queen Mary University of London, London, UK; [16]Ellison Institute of Technology, Oxford, UK; [17]Department of Statistics, University of Oxford, Oxford, UK; [18]The Pioneer Centre for SMARTbiomed, Big Data Institute, Li Ka Shing Centre for Health Information and Discovery, University of Oxford, Oxford, UK
[19]These authors contributed equally
[20]These authors contributed equally
[21]Senior author
*Correspondence: hcm@sanger.ac.uk (H.C.M.), duncan.palmer@stats.ox.ac.uk (D.S.P.)
https://doi.org/10.1016/j.ajhg.2026.04.005.
© 2026 The Authors. Published by Elsevier Inc. on behalf of American Society of Human Genetics.
This is an open access article under the CC BY license (http://creativecommons.org/licenses/by/4.0/).

they represent naturally occurring *in vivo* experiments that can potentially be used to assess the phenotypic consequences of pharmacological inhibition.[1] For instance, individuals with *PCSK9* (MIM: 607786) bi-allelic LoF variants exhibited exceptionally low low-density lipoprotein cholesterol (LDL-C) levels, directly leading to the development of PCSK9 inhibitors for managing hypercholesterolemia.[2] Identifying and characterizing even a single *bona fide* individual with a bi-allelic LoF genotype can inform drug discovery by anticipating potential safety concerns in clinical trials. The recent discovery of a healthy individual with bi-allelic LoF in *HAO1*[3] (MIM: 605023) provided critical validation for the safety of HAO1 inhibition as a treatment approach for primary hyperoxaluria type 1 (MIM: 259900), a therapy now advancing through late-stage clinical trials.[4]

These findings and others have led to a growing interest in characterizing the landscape of bi-allelic inactivation as well as conducting recessive association studies. However, past studies have either focused on gene-level associations in single biobanks[5] or have evaluated evidence on the variant rather than the gene level across biobanks.[6–8] Such constraints have limited statistical power for discovering associations compared to what is theoretically achievable. To our knowledge, no one has attempted to evaluate the gene-level evidence of recessive effects, considering both homozygotes and compound heterozygotes, across multiple biobanks.

Here, we combine data from across six biobanks with diverse genetic ancestries: UK Biobank (UKB), *All of Us* (AOU), the Genomics England 100,000 Genomes Project (100kGP), Genes and Health (G&H), Bio*Me* Biobank (BioMe), and BioBank Japan (BBJ), to analyze the role of rare and low-frequency bi-allelic recessive effects in complex disease and medically relevant quantitative traits. To this end, we conduct phasing to ascertain CH genotypes and perform federated gene-based testing for recessive effects across 41 complex traits and diseases (Figure 1). This work investigates rare and low-frequency recessive effects on health and disease across diverse ancestries and demonstrates the utility of combining large-scale cohorts through global collaboration to replicate known and identify additional recessive associations.

Methods

Study datasets

Informed consent was obtained from all participants in each contributing biobank, and all studies were approved by the relevant institutional and/or national research ethics committees.

The AOU research program, launched by the NIH, is building a cohort of at least 1 million participants from across the United States, with a focus on including populations that have historically been under-represented in biomedical research.[9] AOU collects biospecimens, electronic health records (EHRs), physical measurements, and survey data, and here we consider a set of 389,870 individuals with whole-genome sequencing (WGS) (Table 1), including 165,569 of non-European ancestries.

BBJ is a large-scale, multi-institutional, hospital-based registry launched in 2003 to facilitate personalized medicine through genomic research.[10–14] BBJ has collected DNA, serum samples, and clinical information from approximately 270,000 participants diagnosed with one or more of 51 common diseases, with data linked to EHR. Here, we analyze a subset of 11,462 whole-genome-sequenced individuals, all of which are of East Asian (EAS) genetic ancestry (see "ancestry definition" section below).

BioMe[15] is an EHR-linked biobank established in 2007 by the Icahn School of Medicine at Mount Sinai in New York City. The enrollment approach involves collecting plasma samples and it is non-selective, resulting in a cohort with a high ethnic, socioeconomic, and medical diversity. Here, we worked with a subset of 28,759 BioMe participants having exome sequencing (ES) data with a highly diverse inferred genetic ancestry, such as admixed American (AMR; $n = 8{,}393$), African (AFR; $n = 8{,}814$), European (EUR; $n = 9{,}767$), South Asian (SAS; $n = 973$), and EAS ($n = 812$).

G&H is a long-term, community-based study of British Pakistani and British Bangladeshi individuals aged 16 years and older living in the UK, combining ES and genotyping array data matched with participants' EHR from both primary and secondary care.[6,16,17] Since recruitment started in 2015, more than 60,000 individuals have contributed their genetic, health, and lifestyle data, although here we focus on a pilot subset of 39,148 individuals who had both genotype and ES data, a cohort recently analyzed by Kim et al.[18]

The 100kGP run by Genomics England is a large-scale clinical sequencing initiative within the UK's National Health Service (NHS). It focuses on collecting WGS and clinical data from NHS patients with rare diseases, cancers, and infections.[19,20] In the case of rare disease, 100kGP also recruits relatives in a family-based study design. As a result, 100kGP contains a large number of families within the rare-disease arm: 12,723 individuals in duos, 45,617 in trios, and the remaining being unrelated individuals; this structure is expected to help improve phasing quality for low-frequency variants and, thus, improve the ability to identify CHs.[21]

UKB is a large, population-based, prospective cohort study from the UK comprising genetic, health, and lifestyle data from about 500,000 individuals aged 40–69 years at recruitment (2006–2010).[22] Lassen et al.[5] previously performed a CH analysis using a subset of 175,587 EUR individuals. Here, we expand that, incorporating up to 409,938 ES samples across four ancestries (Table 1).

General quality-control guidelines for biobanks

To ensure consistency across biobanks, general guidelines for variant- and sample-level quality control, ancestry inference, and statistical phasing were collaboratively defined in advance. Each biobank then performed quality control independently, following these shared principles, which we describe in this section. To support practical implementation, analysts applied biobank-specific parameters or thresholds if deemed appropriate.

Variant calling

We processed ES or WGS reads using GATK[23] or Hail.[24] Our workflow included aligning reads to the GRCh38DH reference genome using BWA-MEM, or GRCh37D5 and lifting-over to GRCh38 (i.e., for BBJ),[25] and marking duplicate reads with Picard.[26] We performed base quality score recalibration using GATK

Table 1. Number of damaging bi-allelic genotypes observed across ancestries

		Homozygotes		Compound heterozygotes			CH to homozygote ratio	
**Subcohort**	***n***	**pLoF**	**Damaging-missense**	**pLoF**	**Damaging-missense**	**pLoF| damaging _missense**	**pLoF**	**All damaging**
UKB: AFR	6,597	2,377	2,793	449	516	1,346	0.189	0.447
UKB: EAS	1,647	205	386	14	72	30	0.068	0.196
UKB: EUR	395,325	49,874	96,790	9,588	28,466	13,573	0.192	0.352
UKB: SAS	6,369	1,247	2,793	152	516	259	0.122	0.229
AOU: AFR	79,956	20,569	50,817	NA	N/A	N/A	N/A	N/A
AOU: AMR	72,123	7,938	23,315	N/A	N/A	N/A	N/A	N/A
AOU: EAS	9,441	1,447	3,588	N/A	N/A	N/A	N/A	N/A
AOU: EUR	224,301	23,426	59,619	N/A	N/A	N/A	N/A	N/A
AOU: SAS	4,049	781	2,154	N/A	N/A	N/A	N/A	N/A
100kGP: EUR	62,326	8,978	39,854	2,415	12,831	5,068	0.269	0.416
100kGP: SAS	7,187	4,837	18,547	332	2,004	848	0.069	0.136
G&H: SAS	39,148	14,793	38,316	1,479	5,563	2,895	0.100	0.187
BioMe: AFR	8,814	4,121	8,694	689	1,839	778	0.167	0.258
BioMe: AMR	8,393	940	2,146	152	548	237	0.162	0.304
BioMe: EAS	812	675	920	6	68	19	0.009	0.058
BioMe: EUR	9,767	1,490	3,750	217	576	187	0.146	0.187
BioMe: SAS	973	285	527	24	62	18	0.084	0.128
BBJ: EAS	11,462	1,744	7,307	208	1,673	612	0.119	0.275
Total	948,690	145,727	362,316	15,725	54,734	96,329	0.108	0.190

Total number of samples (*n*) and observed bi-allelic genotypes across subcohorts, measured as homozygous or compound-heterozygous (CH) events. Here, "damaging-missense" refers to the class of damaging-missense/protein-altering variants (low-confidence LoF, variants with REVEL $\geq$0.773 or CADD $\geq$28.1, or splicing variants with SpliceAI $\Delta \geq 0.5$) and pLoF|damaging_missense to individuals who are CH for any combination of pLoF or damaging variants (methods). The last two columns report the ratio of CH to homozygotes for pLoF or for all damaging bi-allelic genotypes (i.e., pLoF|pLoF, damaging-missense|-damaging-missense, or pLoF|damaging_missense). The biobanks included are UK Biobank (UKB), Genes and Health (G&H), 100k Genomes Project (100kGP), BioBank Japan (BBJ), BioMe, and *All of Us* (AOU). N/A, not applicable (AOU was not phased, so CH genotypes were not ascertained).

BaseRecalibrator. Following these steps, we conducted variant calling using GATK HaplotypeCaller to generate gVCF files. Joint calling was then performed using GenomicsDBImport and GenotypeGVCFs. Finally, we applied variant quality score recalibration (VQSR) separately for SNPs or insertions or deletions (indels) using VariantRecalibrator and ApplyVQSR.

Sample and variant quality control

We filtered to sites where at least 85% of samples have a mean coverage of 20× or higher. Individual genotypes were filtered based on allelic depth (total sequencing depth [DP] $\geq$10) and genotype quality (GQ $\geq$ 20). Variants were excluded if they fell within low-complexity regions (LCRs), failed VQSR filtering, or lay outside the padded target intervals for ES data (using 50-bp padding). Samples were excluded based on call rate, mean depth, mean genotype quality, FREEMIX[27] contamination estimates, and proportion of chimeric reads. These thresholds were determined empirically by examining the distribution of the relevant metric.

After these initial filters, we performed a final pass through the data and excluded sites with low call rates or deviation from Hardy-Weinberg equilibrium (HWE, $p < 10^{-10}$). We additionally removed samples falling outside of 5.93 median absolute deviations from the median (corresponding to 4 standard deviations [SDs] from the mean under a normally distributed variable) of the mean transition/transversion ratio, and heterozygous/homozygous alternate, or insertion/deletion ratio, within each ancestry group and sequencing platform.

Sex imputation

We performed sex imputation to confirm that the reported sex matched the genetically inferred sex. To that end, we calculated the *F*-statistic for each sample using the non-pseudoautosomal region on chromosome X and removed samples fulfilling any of these criteria:

- Sex is unknown in the phenotype files
- *F*-statistic $\geq$0.6 for reported females
- *F*-statistic <0.6 for reported males
- *F*-statistic >0.6 with <100 calls on the Y chromosome

Ancestry definition

To guard against spurious associations driven by population stratification in our analysis, we define ancestry labels to subset our analyses to within each biobank before meta-analyzing the

results. We inferred genetic ancestry, considering five super-populations (AFR, AMR, EUR, EAS, SAS) based on genetic similarity to reference populations from the 1,000 Genomes Project (1kGP).[28] Henceforth, we use these acronyms to imply genetically inferred ancestry from the indicated continental region. We first performed principal-component analysis (PCA) on the 1kGP samples, using LD-pruned autosomal variants, then projected the biobank samples onto this PCA space. Next, we trained a random-forest classifier on the super-population labels of 1kGP and used the model to predict the super-population label for each biobank sample. These genetically inferred ancestry labels are based on genetic similarity to 1kGP reference groups and are not intended to represent self-identified race/ethnicity or sociocultural identity.

Variant annotation

Variants were annotated using VEP v105.[29] In summary, we employed the LOFTEE v1.04[30] plugin to classify protein truncating variants (PTVs) as high-confidence or low-confidence LoF variants. Missense and protein-altering variants were annotated using CADD v1.6[31] and REVEL v7[32] scores from DBNSFP v4.3,[33] with CADD v1.6 separately applied to indels. We also used SpliceAI v1.3[34] to annotate variants with splice information. We restricted our analysis to matched annotation from the NCBI and EMBL-EBI (MANE) Select transcripts when available, otherwise defaulting to GENCODE v39[35] canonical transcripts. Variants were hierarchically classified as follows:

(1) pLoF: high-confidence LoF filtered by LOFTEE
(2) Damaging-missense/protein-altering: low-confidence LoF, variants with REVEL-Score $\geq$ 0.773 or CADD-Phred score $\geq$28.1, or splicing variants with SpliceAI Δ score $\geq$0.5
(3) Other missense: missense/start-loss/stop-loss/in-frame indel not categorized above
(4) Synonymous: synonymous variants with SpliceAI Δ score <0.2

Statistical phasing and recessive burden calculation

To facilitate phasing of genetic data across biobanks, we developed a SHAPEIT5[36] wrapper using snakemake.[37] This wrapper was designed for easy sharing and integration into various biobank workflows and was deployed to phase genotypes in G&H, BBJ, and BioMe. In this pipeline, phasing is conducted on single-chromosome chunks after combining ES and array data, with exome variants prioritized in cases of overlap. The phasing process employs a two-stage approach: first, common variants (MAF > 0.001) are phased, which then serve as a scaffold for phasing the rare variants. This method enables efficient and accurate phasing of both common and rare genetic variants across large-scale biobank datasets.

Statistical phasing for UKB has already been extensively described in Lassen et al.[5] Here, we expand that effort by considering the 409,938 ES samples available after quality control, following a similar two-step procedure with SHAPEIT5[36] as described above. For 100kGP, we used the phased genotypes provided by Shi et al.,[21] who performed additional variant- and sample-level quality control prior to phasing (Note S2). Lastly, we did not phase AOU due to limited computational resources; we instead devised recessive burden scores by only considering homozygous genotypes.

We assessed phasing accuracy using read-backed phasing and trio switch error rates (SERs). For G&H, we calculated SERs using 100 trios (272 samples) of SAS ancestry, while, for UKB, we used 99 trios (297 samples) of EUR ancestry. In the case of BBJ, where trios were not available, we conducted read-backed phasing on all autosomes using WhatsHap.[38] We provide all details in Note S3. Phasing accuracy was not explicitly validated in 100kGP, as trios had already been used to perform transmission phasing,[21] nor in BioMe, as no trios were available.

We developed a custom C++ tool that we deployed across environments to identify and annotate individuals with bi-allelic genotypes. Using phased data, we collapsed rare and low-frequency variants (MAF <5%) across individual haplotypes or gene copies. An individual was classified as mono-allelic if only a single gene copy was affected and bi-allelic if both gene copies were affected, thus being homozygous or CH. Using this terminology, we modeled effects based on either the recessive encoding ([0, 0, 1]), or the additive encoding ([0, 1, 2]), reflecting the number of haplotypes affected in that individual. (We note that the latter is different from the standard additive gene burden testing, which tests for an association between the [potentially weighted] sum of qualifying variants within each individual, rather than the number of gene copies carrying at least one qualifying variant.) We refer to these as the rare recessive burden and the corresponding additive burden, respectively. This classification was applied to different sets of variant annotations, assigning equal weights on each variant (or pair of variants), yielding the following types of burden scores: (1) pLoF, (2) pLoF or damaging-missense/protein-altering (referred to as pLoF|damaging_missense), (3) pLoF or damaging-missense/protein-altering or other missense (referred to as nonsynonymous), (4) synonymous (as a negative control).

Phenotype curation

Phenotypes were selected by representatives from each biobank through nomination of International Classification of Disease (ICD)10 and ICD9 case and control inclusion/exclusion criteria, resulting in a list of 32 binary and nine quantitative phenotypes (Tables S1 and S2). The selected disease endpoints encompassed a broad spectrum of health conditions, including cardiovascular diseases, respiratory disorders, various cancers, and age-related conditions. These ranged from common conditions such as hypertension (prevalence of 31.9% with 249,823 cases across all subcohorts) and type 2 diabetes (11.5%, 92,086 cases) to rarer and sex-specific conditions including cervical cancer (0.4%, 921 cases) and female infertility (0.7%, 1,513 cases). For the six female-specific phenotypes we considered, only the female samples were included in the analysis. No quantitative traits were available for 100kGP. See Note S1 for more details on phenotyping.

Association analysis

We performed association testing with the recessive burden using SAIGE[39] or Regenie.[40] Both tools are scalable implementations of a two-step association testing framework that control for covariates and allow for sample relatedness and high case-control imbalance. Each analysis was carried out separately for each ancestry group (hereafter referred to as "subcohort"). For SAIGE, we constructed sparse genetic relatedness matrices (GRMs) using 2,000 randomly selected markers and a relatedness cutoff of 0.05.[41] We included age, age^2, sex, age·sex, age^2·sex, and first 10 genetic principal components (PCs) as fixed-effect covariates. In the UKB analysis, we also included sequencing batch and recruitment center. Quantitative traits were subjected to inverse-normal transformation. For binary traits, Firth correction was applied

for tests with $p < 0.01$. For each ancestry within each biobank, we tested for an association between recessive burden and phenotype among (gene, phenotype) pairs with at least five individuals harboring bi-allelic genotypes and available phenotype data. This restriction results in distinct collections of gene-based tests for each (biobank, ancestry, trait) tuple. Lastly, we tested the additive burden across all available genes, retaining only those genes for which recessive summary statistics were also available.

Meta-analysis

After collecting statistics across biobanks we conducted a fixed-effects meta-analysis using Stouffer's method.[42] This approach weights p values from each study by their effective sample size (N_{eff}). Specifically, the method converts p values to signed Z scores, which are then combined in a weighted sum

$$Z = \frac{\sum_{i=1}^{k} \sqrt{N_{\text{eff,i}}} \cdot \left(- \Phi^{-1}(p_i) \right) \cdot \text{sign}(\beta_i)}{\sqrt{\sum_{i=1}^{k} N_{\text{eff,i}}}}$$

where k is the number of studies (i.e., subcohorts within a biobank), N_{eff} is the effective sample size of study i, p_{i} is the p value from study i, Φ^{-1} is the inverse standard normal cumulative distribution function, and sign(β_{i}) is the direction of effect in study i. The effective sample size for subcohort i, $N_{\text{eff},i}$, and binary traits is defined using the number of cases and controls in each subcohort as

$$N_{\text{eff},i} = \frac{4}{\frac{1}{N_{1,i}} + \frac{1}{N_{\text{controls},i}}}$$

whereas, for quantitative traits, it is equal to the number of individuals in the analysis. The meta-analysis p value was then computed as $p = 1 - \Phi(Z)$ for one-tailed tests, or $p = 2(1 - \Phi(|Z|))$ for two-tailed tests, where Φ is the standard normal cumulative distribution function (CDF).

As a follow-up analysis, we performed an inverse-variance weighted meta-analysis to obtain estimates of effect sizes for our recessive associations and estimated the total number of individuals considered in each underlying test. To adhere to privacy restrictions when reporting genotype counts (e.g., less than 20 for AOU), we provide a lower and an upper bound instead of a single count when appropriate.

We further tested for heterogeneity among studies using Cochran's Q test.[43] The meta-analytic effect size was calculated as

$$\overline{\beta} = \frac{\sum_{i=1}^{k} \omega_i \beta_i}{\sum_{i=1}^{k} \omega_i}$$

where β_i is the effect size of study i, and $w_{\text{i}} = N_{\text{eff},i}$ is the weight assigned to study i. Then, Cochran's Q statistic was computed as

$$Q = \sum_{i=1}^{k} \omega_i (\beta_i - \overline{\beta})^2.$$

Under the null hypothesis of homogeneity, Q follows a χ^2 distribution with $k-1$ degrees of freedom, where k is the number of studies. The p value for the heterogeneity test was calculated as $p_{\text{het}} = p(\chi^2_{\text{k}-1} > \text{Q})$. This test quantifies the extent of between-study variability in effect sizes beyond what would be expected by chance. We then inspected cohort-specific estimates and classified heterogeneity into three patterns based on concordance of effect direction among the largest subcohorts and the presence of discordant, typically imprecise estimates from smaller subcohorts.

Cauchy combination of p values across annotation masks

Following Liu and Xie[44] and recent rare-variant association studies,[45,46] we compute a single p value per gene-trait pair using the Cauchy-combination test (CCT), which allows us to combine multiple correlated p values per gene-trait pair. For d individual p values p_i, $i = 1, 2, \ldots, d$, the CCT statistic is defined as:

$$T_{\text{Cauchy}} = \sum_{i=1}^{d} \omega_i \tan((0.5 - P_i)\pi)\cdot$$

The weights ω_i must be non-negative and sum to one. Here, we set $\omega_i = 1/d$ for all i (uniform weights). The transformation $\tan((0.5 - p_{\text{i}})\pi)$ follows the standard Cauchy distribution if each p_i follows the null distribution, i.e., is uniformly distributed between 0 and 1. The combined p value can therefore be evaluated as $p_{\text{Cauchy}} = F_{\text{C}}(T_{\text{Cauchy}})$, where $F_{\text{C}}(\cdot)$ is the CDF of the standard Cauchy distribution. When applicable, we used the CCT to combine the p values from the pLoF, pLoF|damaging_missense, and nonsynonymous tests.

Power comparison between meta-analysis and UKB

To assess the gain in statistical power achieved through meta-analysis compared to using the UKB:EUR subcohort (our largest one), we compared χ^2 statistics derived from both approaches. Our meta-analysis incorporated data from up to six biobanks. For each gene-trait association pair, we converted p values from both the UKB:EUR analysis (p_{UKB}) and the meta-analysis (p_{META}) to χ^2 statistics (1 degree of freedom) using the inverse CDF (F^{-1}) of the χ^2 distribution. We then calculated the ratio of these χ^2 statistics ($\chi^2_{\text{META}}/\chi^2_{\text{UKB}}$), which approximates the increase in relative effective sample size under the assumption of a fixed-effect size across the analyzed subcohorts. This ratio serves as an estimate of power gain, with a ratio greater than 1 indicating increased statistical power in the meta-analysis compared to the UKB alone.

Additional analyses for significant associations

We performed additional tests for several of the reported associations to distinguish putative recessive associations from signals likely driven by previously established effects. This included the association between *PYGM* and aspartate aminotransferase (AST) in UKB:EUR and AOU:EUR, for which we repeated testing while conditioning on glycogen storage disease (ICD10: E74), since *PYGM* is a recessive gene for this disorder (MIM[47]: 608455; see Note S6). Likewise, we re-evaluated all our significant associations with *HBB* (MIM: 141900), a gene that has well-established recessive effects on β-thalassemia (MIM: 613985) and sickle-cell disease (MIM: 603903). We did so by conditioning on disease status for thalassemia, sickle-cell disorders, or hereditary hemolytic anemias in AOU:AFR (Phecodes[48]: GE_970.2, GE_970.1, GE_970.6, respectively) and G&H:SAS (ICD10: D56, D57, D58). Finally, we followed up on the association between bi-allelic variation in *LECT2* (MIM: 602882) and height, repeating testing after dropping bi-allelic genotypes involving rs62623707, a known additive association.[49]

Results

Following quality control in each biobank (methods), our analysis included 410,000 samples from UKB, 390,000

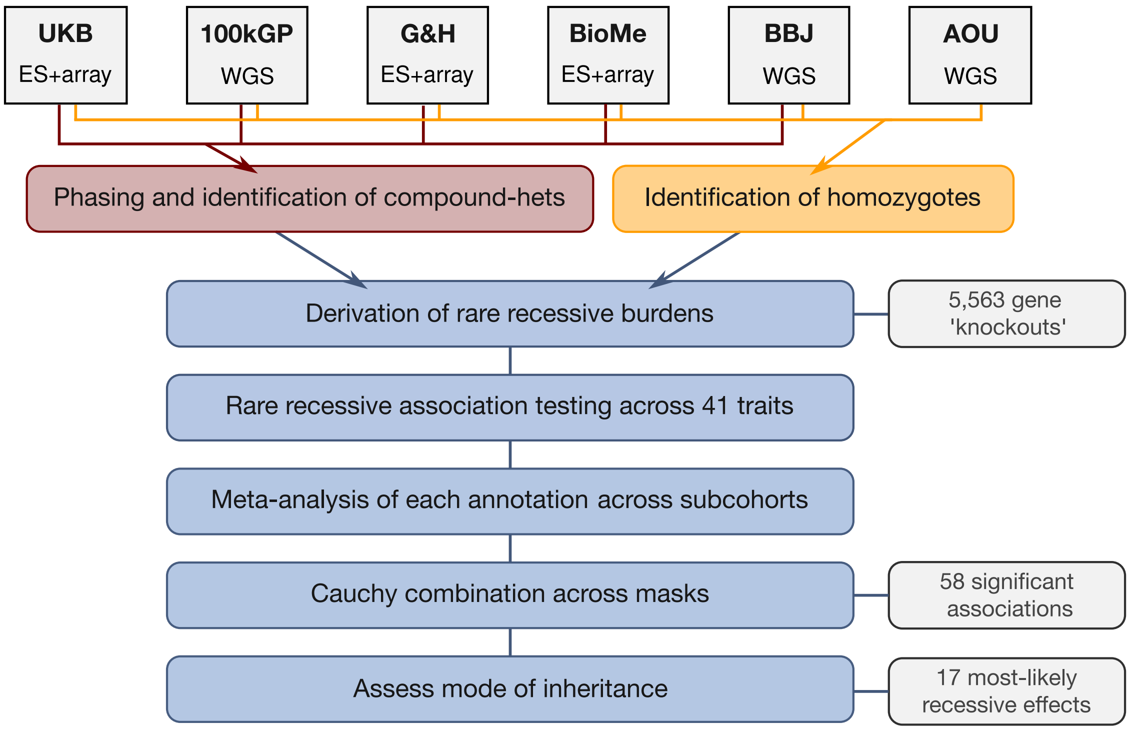

Figure 1. Flowchart summarizing analyses within this study
UK Biobank (UKB), Genes and Health (G&H), 100,000 Genomes Project (100kGP), BioMe, BioBank Japan (BBJ), and *All of Us* (AOU) were included in our study, totaling 948,690 samples. For 100kGP, BBJ, and AOU, we worked with whole-genome sequencing (WGS), whereas, for the remaining biobanks, we jointly processed exome sequencing (ES) and genotyping array data. All biobanks were phased besides AOU (methods) and up to 41 binary and quantitative traits were analyzed across biobanks. We performed association testing in up to four annotation masks in each biobank and meta-analyzed all (mask, gene) test statistics across biobanks using Stouffer's method.[42] We then combined the resultant meta-analyzed p values across masks using the CCT[44] and compared recessive to additive models to infer mode of inheritance.

from AOU, 70,000 from 100kGP, 39,000 from G&H, 29,000 from BioMe, and 11,000 from BBJ (Figure 2; Table 1). The combined sample was predominantly of European genetic ancestry (72.9%, $n = 691{,}719$), largely due to UKB's composition (96.4% EUR) (Figure 2). The remaining 256,971 individuals of non-European ancestry were distributed across multiple ancestry groups, with AFR and AMR ancestry representing the second- and third-largest groups with 95,000 and 81,000 individuals, respectively.

Characterizing bi-allelic variation across biobanks

Until recently, investigations of phenome-wide consequences of bi-allelic variants have mainly focused on homozygosity[6–8,50] or have been limited to a single cohort.[5] To identify CH variants, we statistically phased rare and low-frequency (MAF $\leq$ 0.05) variants ascertained from ES or WGS in each biobank using SHAPEIT5.[36] We restricted our analysis to confidently phased genotypes with posterior probability (PP) >0.90, ensuring high-confidence CH variant calls. For 100kGP, we used phased quality-controlled WGS data from Shi et al.[21] (methods). We were unable to phase AOU due to the computational intensity of phasing WGS datasets, and thus we only considered homozygous and not CH genotypes in that biobank (Figure 1).

Next, we evaluated phasing accuracy across ancestries and biobanks using trios (when available) or read-backed phasing of short-read sequencing data (Note S3; Figure S2; Table S11). Across cohorts, statistical phasing showed low overall SERs, with elevated errors confined to ultra rare variants (minor allele count $\leq$ 5). Importantly, restricting to genotypes with PP > 90%, as used in all downstream analyses, substantially reduced error rates across all ancestries and allele frequencies, such as 0.19% (UKB), 0.32% (G&H), or 0.30% (BBJ). These results demonstrate the reliability of the phasing framework and support the use of the obtained CH genotypes in subsequent analyses.

Including CH variants significantly enhanced our analysis of bi-allelic pLoF genotypes, increasing their number by 17.2% from 91,566 to 107,291 (Table 1, excluding the AOU subcohorts). The extent of this increase varied across subcohorts, reflecting differences in consanguinity within them (e.g., 18.7% in G&H, 27.5% in BBJ, and 41.6% in European individuals from 100kGP). To quantify the change in the number of genes available for testing, we restricted to genes with at least five individuals harboring a pLoF genotype (the threshold used in the association tests below) and found that 1,151 genes would have been tested (across five biobanks); CH variation

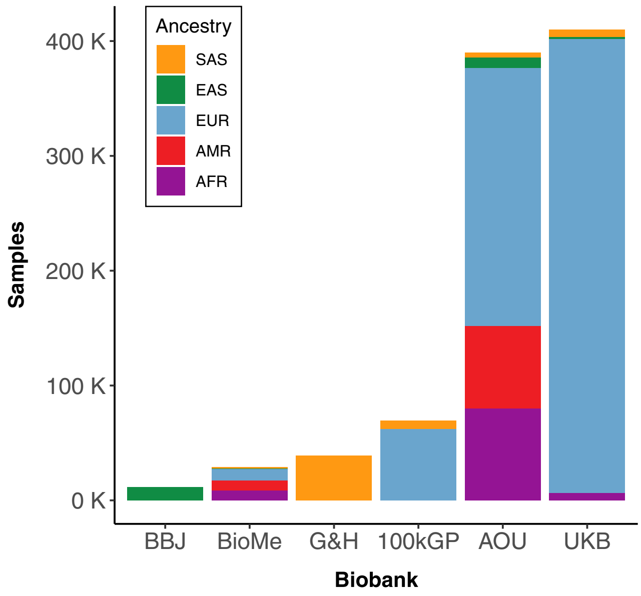

Figure 2. Sample sizes of biobanks analyzed in our study
Barplot showing the overview of quality-controlled samples available for downstream analyses, stratified by biobank and ancestry assignment. This plot highlights the contribution of each biobank; refer to Table 1 for the exact counts.

increased the collection of testable genes by 8.9%, to 1,253 genes (Table S3). When considering combinations of pLoF or damaging-missense variants, incorporating CH genotypes increased the number of testable genes by 11.1% (4,469 vs. 4,966). Overall, incorporating CH variants provided a considerable expansion of our dataset to detect rare bi-allelic effects phenome-wide.

There has been long-standing interest in identifying bi-allelic gene inactivation (gene knockouts),[51–54] with a recent large-scale study reporting 4,848 such genes in a sample of 983,578 individuals.[55] In our analysis of six biobanks—five of which included phased data—we surveyed pLoF variants with MAF < 1% (to match previous studies) and identified bi-allelic genotypes in 5,563 genes, expanding the known set of genes with knockouts by 1,767 (+19.8%, for a total of 8,925 unique genes). Notably, 1,371 of these additional genes were found in individuals of non-European ancestry, concentrated in SAS subcohorts (1,111 genes), underscoring the importance of including diverse populations even in modest sample sizes, particularly if there is increased autozygosity.[17] See Note S4 for more details and Table S4 for the complete gene list.

Meta-analysis of rare recessive association studies

We performed recessive gene-based association testing between bi-allelic variation in (biobank, ancestry) pairs and up to 41 phenotypes (32 binary and nine quantitative traits; Figure S1; Tables S1 and S2). Specifically, we split each biobank into genetically inferred ancestry groups, analyzed each of these separately, and meta-analyzed across subcohorts. Our analysis incorporated the following variant consequence combinations, which we refer to as “masks”: pLoF, pLoF|damaging_missense (see methods for a detailed definition), nonsynonymous (i.e., pLoF plus all missense/protein-altering), and synonymous variants as a negative control (methods). We assessed the summary statistics for inflation by calculating λ_{95} (the genomic inflation factor calculated at the 95th percentile) for each annotation mask and each subcohort. The vast majority of tested traits were well calibrated, with a median λ_{95} of 0.96 for pLoF tests or 0.98 for pLoF|damaging_missense across subcohorts (Note S5; Figures S3, S4, and S5).

Next, for each predicted damaging annotation mask (pLoF, pLoF|damaging_missense, or nonsynonymous), we performed a fixed-effects meta-analysis across subcohorts using Stouffer's method.[42] We then calculated a single p value per gene-trait pair by combining the meta-analyzed p values across annotations using the CCT (methods). Following our meta-analysis, the Cauchy-combined p values displayed no systematic inflation ($0.83 < \lambda_{95} < 1.20$; median $= 0.99$; Figures S3, S4, and S6). As a negative control, we meta-analyzed p values from the synonymous burden tests, which were also well calibrated ($0.85 < \lambda_{95} < 1.16$; median $= 0.96$; Figure S6).

Our meta-analysis of predicted damaging variants included 41 traits and up to 17,726 genes, resulting in 657,048 tests following the Cauchy-combination step. To account for multiple testing, we applied a Benjamini-Hochberg correction with FDR < 0.01, corresponding to a p value threshold for significance of 7.53×10^{-7}. Using Cauchy-combination p values, we identified 58 significant associations spanning 39 unique genes and 18 traits (Table S5; Figure 3B). Five meta-analyzed synonymous bi-allelic association tests were significant (FDR < 0.01; $p < 3.42 \times 10^{-8}$; Table S6). All five have been previously implicated in association studies of the same phenotype,[56,57] providing reassurance that our framework is well calibrated in terms of false positives (Note S5). Of the 58 significant gene-phenotype associations reported, the pLoF mask was tested in 14, of which five were significant. In all of these cases, the pLoF mask was the most significant (Table S5). Lastly, we found that 19 of the 58 significant gene-trait associations showed evidence for between-cohort heterogeneity (Cochran's Q, $p_{het} < 0.05/89$; Table S7) for at least one annotation, corresponding to 29 mask-specific tests. Inspection of cohort-specific estimates revealed three recurring patterns: concordant directions with differing magnitudes among all contributing subcohorts (7/29); concordant directions among the largest cohorts with a small number of subsignificant, direction-discordant estimates from smaller subcohorts (16/29); and discordant directions involving both smaller and larger cohorts (6/29). These patterns suggest that most heterogeneity reflects differences in cohort ascertainment and power, particularly in subcohorts with limited numbers of individuals harboring bi-allelic

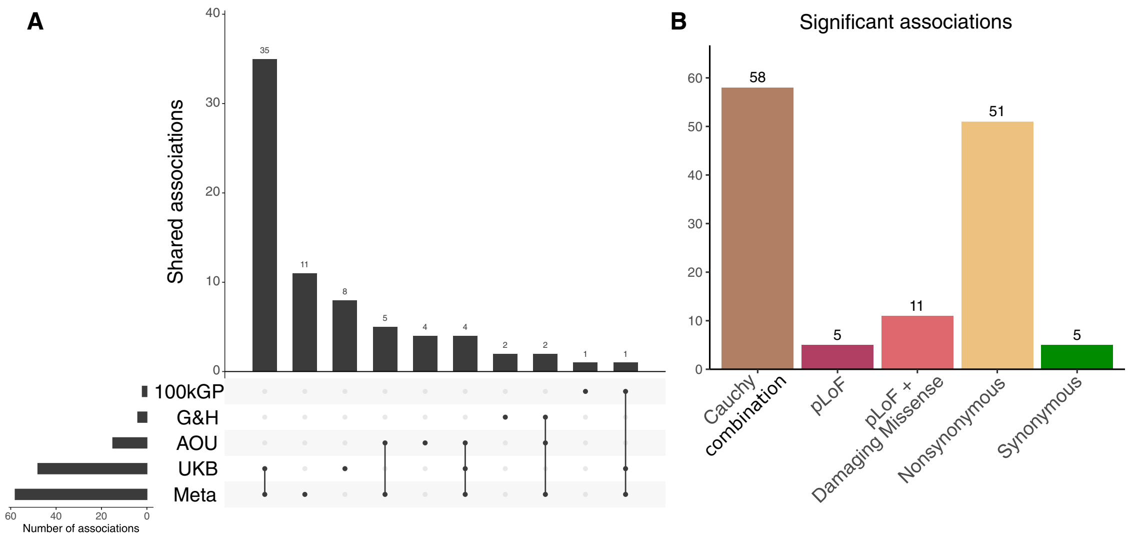

Figure 3. Overview of recessive associations by biobank and variant annotation
(A) Upset plot and bar plot (inset) showing the number of significant (FDR < 0.01) recessive associations discovered in each biobank and between biobanks. To obtain the number of significant hits discovered in each biobank, we first meta-analyzed all subcohorts (if any) and then combined all available annotation specific *p* values with the CCT. This resulted in two, four, 15, and 48 for 100kGP, G&H, AOU, and UKB respectively, whereas BioMe and BBJ did not yield any significant associations.
(B) Number of significant associations ($p < 7.53 \times 10^{-7}$) in meta-analysis per variant mask, and combination of *p* values across masks through Cauchy combination (except synonymous). See Table S5 for the exact *p* values.

genotypes, rather than inconsistent evidence for association in the largest contributing cohorts.

To assess the value of the meta-analysis, we investigated how many associations would have been discovered if each biobank had been analyzed separately. We followed the methodology in the primary analysis meta-analyzing subcohorts within biobank with Stouffer's method before calculating within-biobank FDRs, adjusting for the varying number of genes tested per biobank, and applying the CCT across annotation masks within each biobank. As expected due to its large sample size, UKB yielded the highest number of significant recessive associations ($n = 48$; Figure 3A). Interestingly, of the 15 significant associations in AOU, eight were found solely in AFR individuals; these all involved *HBB*, for which no bi-allelic EUR individuals were observed.

We also investigated whether our meta-analysis improved association power by comparing χ^2 statistics derived from *p* values in recessive tests of the UKB:EUR subcohort (the largest subgroup in our study) to the χ^2 from the meta-analysis (Figure S7A). Of the original 58 significant (FDR < 0.01) recessive gene-trait associations, we were able to compare 52 gene-trait pairs with at least five individuals harboring bi-allelic genotypes in UKB:EUR. Among these, 39 (75%) demonstrated higher χ^2 statistics in the meta-analysis compared to UKB, one showed almost no change in χ^2 (to one decimal place precision), and 12 (23.1%) had higher χ^2 in UKB compared to the meta-analysis; in total, the meta-analysis showed a median increase in χ^2 of 14.7%. We observed notable increases in χ^2 statistics for certain associations, such as for *BTNL9* (MIM: 620648) and high-density lipoprotein cholesterol (HDL-C) ($\chi^2_{\text{UKB}} = 64.3$, $\chi^2_{\text{meta}} = 99.1$) or *MUTYH* (MIM: 604933) and benign and *in situ* intestinal neoplasms ($\chi^2_{\text{UKB}} = 34.5$, $\chi^2_{\text{meta}} = 66.7$), exemplifying the general gain in power achieved through meta-analysis. However, we also observed instances where analyzing a single biobank alone yielded stronger associations, such as *FLG* (MIM: 135940) with asthma ($\chi^2_{\text{UKB}} = 259.8$, $\chi^2_{\text{meta}} = 222.1$). This variability highlights a key trade-off of a cross-biobank meta-analysis: while it often boosts power, it can also attenuate strong signals when variant effects vary across cohorts or are aggregated with differing frequencies and functional impact, as in the case of the nonsynonymous burden.[58]

Comparing recessive and additive genetic models

To determine whether a significant gene-trait association is more likely to be recessive rather than tagging an additive effect, we determined the corresponding additive burden (0, one, or two haplotypes affected; methods) for each annotation and tested for association in each biobank. We then performed an additive meta-analysis across biobanks for each annotation mask followed by the CCT as in the recessive meta-analysis described above. Finally, we adopted the heuristic introduced by Heyne et al.[50] and considered that a recessive mode of inheritance was more likely if the recessive *p* value was more than two orders of magnitude smaller than its additive counterpart (see Note S7 for details and an alternative approach).

Of the 58 significant recessive gene-trait meta-analyzed associations, 17 (spanning 10 genes and 11 traits) fulfilled this criterion (Table 2). Importantly, several of these genes were listed in OMIM[47] with a recessive mode of

Table 2. Overview of the 17 recessive associations discovered through meta-analysis

Binary

Phenotype	Gene	p_{rec}	p_{add}	$\exp(\beta_{rec})$ (OR)	Other studies	Comments
Chronic obstructive pulmonary disease	*ODAD1*	5.0×10^{-7}	0.58	13.99	MIM: 615038 (AR)	likely reflecting misdiagnosis of primary ciliary dyskinesia (MIM: 615067), known to be caused by AR variants in this gene[65,66]
Benign intestinal neoplasm	*MUTYH*	3.2×10^{-16}	3.2×10^{-5}	3.18	MIM: 604933 (AR)	known AR cause of multiple colorectal adenomas[59]
Colon and rectal cancer		1.0×10^{-15}	0.25	3.68		
Asthma	*FLG*	3.1×10^{-50}	6.5×10^{-34}	1.65	MIM: 135940 (AR)	known AR cause of ichthyosis vulgaris (MIM: 146700), often accompanied by asthma[60]
Chronic obstructive pulmonary disease	*SERPINA1*	7.4×10^{-19}	3.8×10^{-6}	1.32	MIM: 107400 (AR)	known AR cause of emphysema due to alpha-1 antitrypsin (AAT; MIM: 613490) deficiency[67]
Inflammatory bowel disease	*NOD2*	3.0×10^{-7}	9.5×10^{-4}	1.14	MIM: 605956 (Mu)	known AR cause of early-onset Crohn disease[62]
Heart Failure	*HBB*	2.6×10^{-14}	0.98	2.64	–	conditional analyses suggest these are not fully attributed to haemoglobinopathies, e.g., sickle-cell disease (MIM: 603903) or β-thalassemia (MIM: 613985), known to be due to *HBB* mutations.
**Continuous**	–	–	–	β_{rec} (SD)	–	
LDL cholesterol	*HBB*	6.1×10^{-15}	5.5×10^{-11}	−0.46	Koyama et al.[7] (AR)	
Total cholesterol		3.6×10^{-25}	6.0×10^{-9}	−0.54	Koyama et al.[7] (AR)	
Aspartate aminotransferase		3.2×10^{-26}	2.2×10^{-2}	0.45	–	
BMI		3.6×10^{-14}	5.6×10^{-3}	−0.30	–	
HDL cholesterol		1.7×10^{-23}	4.9×10^{-4}	−0.54	Nielsen et al.[68] (add)	
HDL cholesterol	*BTNL9*	2.3×10^{-23}	9.3×10^{-15}	−0.48	Koyama et al.[7] (AR)	knockouts have higher T cell activation in adipose/gut, which could lead to lipid derangements[69,70]
Triglycerides		2.5×10^{-7}	0.57	0.26		
Height	*LECT2*	3.7×10^{-14}	4.1×10^{-10}	−0.05	DeWan et al.[71] (add)	missense variant rs62623707 has been fine-mapped as likely causal[72]
Height	ENSG00000267561	2.9×10^{-9}	0.37	0.83	–	previous studies have associated intronic variants in the region; our results may point to the causal gene
Aspartate aminotransferase	*PYGM*	8.2×10^{-10}	0.60	1.00	–	mediated by glycogen storage disease V (MIM: 232600), known to be due to AR mutations in this gene[73]

We report the significant associations (FDR < 0.01) that most likely have a recessive mode of inheritance ($p_{rec} < p_{add}/100$; methods). The table is split into binary (top) and quantitative (lower) phenotypes, reporting recessive odds ratios (ORs) and effect size estimates (β_{rec}, in SD units of the transformed trait), respectively, of the most significant mask after inverse-variance meta-analysis (see Table S5 for all estimates). We also report related entries in Online Mendelian Inheritance in Man (MIM) (if any) and information we gathered in support of each case from the GWAS Catalog[57] or Open Targets.[72] AR, autosomal recessive; Mu, multiple inheritance modes; add, additive (from genome-wide association study [GWAS]).

inheritance for the same or related conditions, serving as positive controls that validate our approach. These included *MUTYH* (MIM: 604933)[59] with colon/rectal cancer ($p_{rec} = 1.0 \times 10^{-15}$) and with benign and *in situ* intestinal neoplasms ($p_{rec} = 3.2 \times 10^{-16}$), *FLG* (MIM: 135940) with asthma ($p_{rec} = 3.1 \times 10^{-50}$),[60] *SERPINA1* (MIM: 107400) with chronic obstructive pulmonary disease (COPD) ($p_{rec} = 7.4 \times 10^{-19}$),[61] and *NOD2* (MIM: 605956) with inflammatory bowel disease (IBD) ($p_{rec} = 3.0 \times 10^{-7}$). Common and rare variants at the *NOD2* locus are known to be associated with IBD, predominantly determined via additive association testing, although rare recessive mutations have been reported to cause early-onset Crohn disease.[62–64]

Table S5 indicates which mask was most significant for each of the 58 gene-trait associations. Five were most significant for the pLoF mask; of these, 4/5 were classified as more likely to be recessive ($-\log_{10}(p_{rec}) > -\log_{10}(p_{add}) + 2$). Five were most significant for the pLoF|damaging_missense mask (three, 60%, classified as recessive) and 48 for the nonsynonymous mask (10/48, 21%, classified as recessive). Fisher's exact testing showed that the pLoF proportion was significantly higher than the nonsynonymous one ($p = 0.014$), suggesting that recessive models with

more strict variant masks (e.g., pLoF) are more likely to ca pture true recessive effects. This finding is in line with the observation that the vast majority of recessive Mendelian disease genes have an LoF mechanism, whereas domina nt-acting genes may have loss- or gain-of-function mechanisms.[74]

Details of likely-recessive associations found

Individuals with bi-allelic *BTNL9* variants exhibited a significant reduction of HDL-C ($p_{rec} = 2.3 \times 10^{-23}$ and $p_{add} = 9.3 \times 10^{-15}$) and higher triglyceride levels ($p_{rec} = 2.5 \times 10^{-7}$ and $p_{add} = 0.57$). Across biobanks and ancestries, the association with HDL-C was strongest among European-ancestry individuals from AOU and UKB with bi-allelic pLoF genotypes (AOU, $p_{rec} = 6.2 \times 10^{-9}$; odds ratio [OR] = 0.46, 95% confidence interval [CI] = [0.36, 0.60]; UKB, $p_{rec} = 3.2 \times 10^{-16}$; OR = 0.65, 95% CI = [0.59, 0.72]; Figure 4). A similar pattern was observed for triglycerides (AOU, $p_{rec} = 0.029$; OR = 1.36, 95% CI = [1.03, 1.79]; UKB, $p_{rec} = 3.5 \times 10^{-6}$; OR = 1.28, 95% CI = [1.15, 1.42]). We also observed a strong signal when considering all nonsynonymous bi-allelic variants in AOU ($p_{rec} = 5.2 \times 10^{-8}$, OR = 0.57 95% CI = [0.47, 0.70]), with six of the seven subcohorts exhibiting the same direction of effect (Figure 4). *BTNL9* encodes an immunoregulatory protein that modulates T cell activation and its dysfunction may contribute to HDL deficiency by promoting inflammation that disrupts cholesterol metabolism.[69,70] The genetic link between *BTNL9* and HDL-C or triglycerides is supported by a growing number of recent sequencing-based studies, such as an additive association of the pLoF rs200884524 detected in a Polynesian cohort,[75] and recessive effects of rs367635312,[7] a pLoF that also drove the signal of association in our study (MAF ≈ 1% in UKB and AOU).

We found significant ($p < 5.0 \times 10^{-7}$) associations between *HBB* and a variety of traits, including total cholesterol ($p_{rec} = 3.6 \times 10^{-25}$), HDL-C ($p_{rec} = 1.7 \times 10^{-23}$), LDL-C ($p_{rec} = 6.1 \times 10^{-15}$), AST ($p_{rec} = 3.2 \times 10^{-26}$), body mass index (BMI) ($p_{rec} = 3.6 \times 10^{-14}$), and heart failure ($p_{rec} = 2.6 \times 10^{-14}$), with more significant evidence of association under a recessive model compared to an additive model (Table S2). These associations were driven by AFR individuals in AOU and SAS individuals in G&H; no EUR individuals harbored bi-allelic pLoF or protein-altering/missense variants (Figure 4). Koyama et al.[7] recently reported similar recessive associations (rs334 with LDL-C and total cholesterol) in AFR individuals that were more significant than additive effect-size estimates fitted to the same data. Associations between *HBB* and both lipid traits and heart failure were seen in G&H:SAS and AOU:AFR individuals, whereas *HBB* associations with AST and BMI were restricted to AOU:AFR individuals (Figure 4). We hypothesized that these effects may represent indirect consequences of hemoglobinopathies caused by defects in *HBB* (MIM: 141900) (for example, treatment effects, blood transfusion, or chronic ill health). Alternatively, they may be direct effects of hemoglobinopathies, the metabolic consequences of which include hypolipidemia (consistent with our findings of negative effects on lipids; Figure 4), driven by increased cholesterol requirements for erythrocyte membrane synthesis coupled with elevated LDL clearance by an overactive reticuloendothelial system.[76]

To test whether the associations between *HBB* and these associated phenotypes were driven by individuals diagnosed with hemoglobinopathies, we repeated our analysis in G&H:SAS and AOU:AFR while conditioning on diagnosis status for β-thalassemia (MIM: 613985), sickle-cell (MIM: 603903) or other hereditary anemias (methods). In all cases, the effect sizes attenuated, but all associations remained significant after meta-analyzing the conditional summary statistics from G&H and AOU (Table S8), suggesting that hemoglobinopathies do not fully account for the associations we detect with *HBB*. When considering the same mask (nonsynonymous), the extent to which the effect sizes changed after conditioning on any hemoglobinopathy diagnosis was similar between the two cohorts for AST and lipids, with an average attenuation of ∼14% (Table S8). For heart failure, the attenuation was stronger in AOU than G&H (before versus after conditioning: $OR_{before,AOU:AFR} = 2.64$, $p = 1.6 \times 10^{-13}$; $OR_{after,AOU:AFR} = 1.88$, $p = 1.2 \times 10^{-6}$; 29% attenuation; $OR_{before,G\&H:SAS} = 2.89$, $p = 2.7 \times 10^{-3}$; $OR_{after,G\&H:SAS} = 2.42$, $p = 7.2 \times 10^{-3}$; 16% attenuation), and, for BMI, there was minimal attenuation in AOU, whereas the effect was not significant in G&H before or after conditioning. These differences between cohorts may reflect differences in the consequences of specific variants that are included in the tests, and/or differences in the types of hemoglobinopathies common among individuals of SAS genetic ancestry in G&H versus individuals of AFR genetic ancestry in AOU. In G&H, the majority (60%) of hemoglobinopathy diagnoses were for thalassemia and 31% were for sickle-cell disorders; in contrast, the equivalent fractions in AOU (AFR) were 45% and 33%, respectively.

Further work is required to characterize the phenotype, treatment, and long-term health outcomes of *HBB* carriers to determine the extent to which the associations we have found are direct or indirect consequences of these different hemoglobinopathies. Nonetheless, our findings align well with the literature. Individuals with β-thalassemia who harbor bi-allelic pathogenic variants in *HBB* show a characteristic cardiometabolic profile—lower total, LDL, and HDL cholesterol and higher triglycerides—distinct from heterozygotes.[77] The link to heart-failure risk is biologically plausible given iron-overload cardiomyopathy documented in thalassemia and sickle-cell cohorts.[78,79] More broadly, population data from Finland demonstrate that lower hemoglobin associates with lower BMI, healthier lipid panels, and reduced

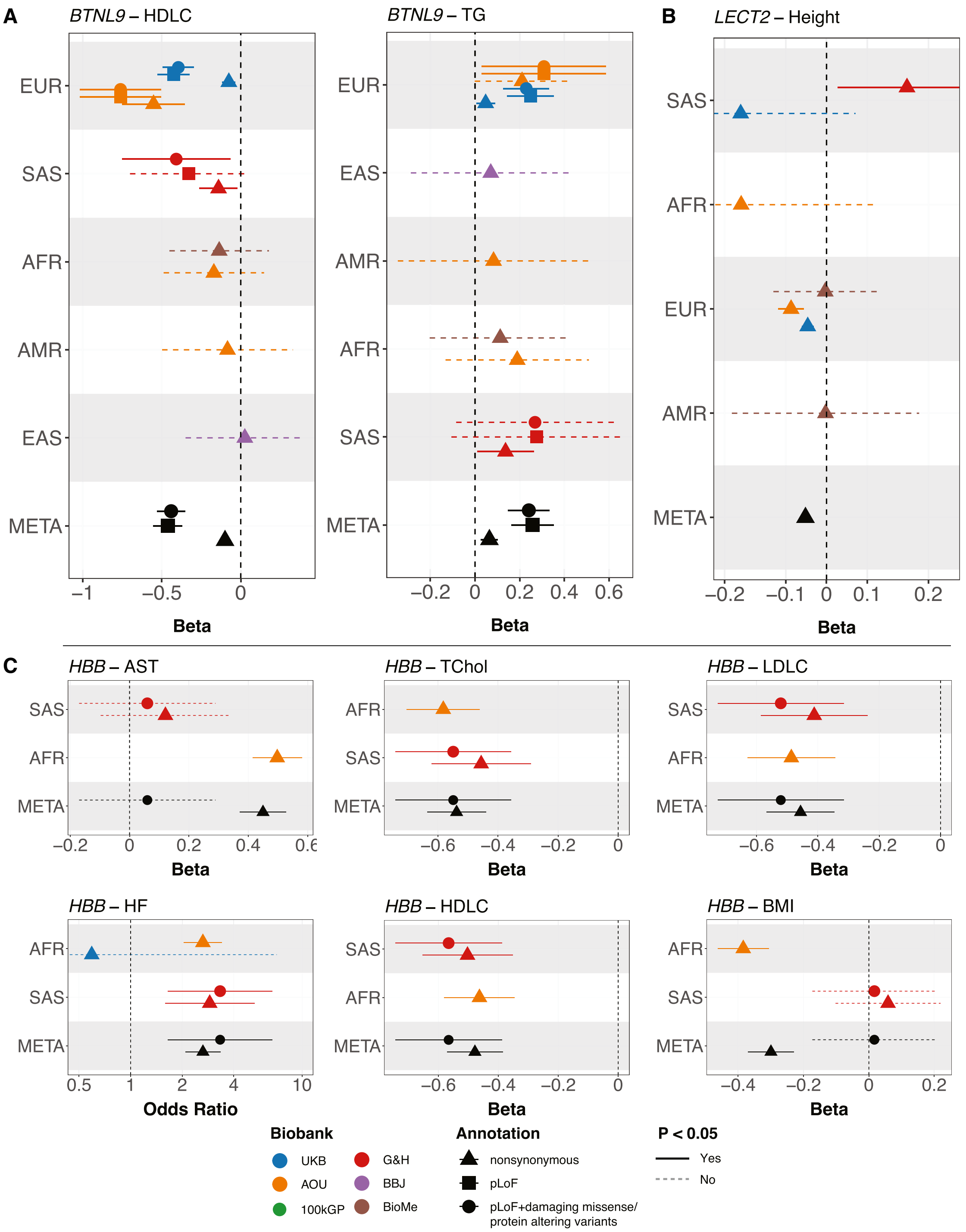

Figure 4. Forest plot of nine putatively recessive gene-trait associations detected by meta-analysis
Forest plot with ORs or effect sizes (betas) and 95% CIs across several recessive gene-trait associations discussed in the text, focusing on *BTNL9* (A), *LECT2* (B), and *HBB* (C), with the remaining displayed in Figure S8. The plot includes pLoF variants (squares), pLoF|damaging_missense (circles), and nonsynonymous (triangles), stratified by ancestry, with different biobanks indicated by different colors. Associations were evaluated only when at least five individuals with bi-allelic genotypes were present, and we display results for associations tested in at least two cohorts and effect size estimates determined through inverse-variance weighted meta-analysis. To aid visualization, large CIs have been truncated, and dashed lines indicate associations with $p_{rec} > 0.05$. TG, triglycerides; AST, aspartate aminotransferase; TChol, total cholesterol; HF, heart failure.

inflammation,[80] mirroring the protective cardiometabolic pattern we observe in HBB knockouts.

Finally, we observed two putatively recessive associations with height: *LECT2* ($p_{rec} = 3.7 \times 10^{-14}$), and an uncharacterized gene, ENSG00000267561 ($p_{rec} = 2.9 \times 10^{-9}$). Both associations were obtained using nonsynonymous variants, with the latter only found in a single cohort (AOU:EUR OR = 2.28, 95% CI = [1.73, 3.00]). For *LECT2*, our association was robustly supported by negative effects in European samples of UKB and AOU, although G&H showed a nominally significant association in the opposite direction (Figure 4). Kichaev et al.[49] identified a missense variant in *LECT2* (rs62623707) associated with height, which was subsequently fine-mapped as likely causal by Open Targets.[72] When we repeated the recessive burden test after excluding rs62623707 (methods), the signal ablated (AOU:EUR $p > 0.5$; UKB:EUR could not be tested as fewer than five bi-allelic genotypes remained), indicating that the observed association is driven entirely by this variant. *ENSG00000267561* is near several common SNPs associated with height by Yengo et al.,[81] including rs1325237 (2.5 kb downstream) and rs486133 (100 kb upstream). Open Targets has prioritized *SELENOF* (MIM: 606254) as the most likely causal gene at this locus, although with only a modest locus-to-gene score of 0.241.[72] *SELENOF* is a plausible candidate, involved in selenium metabolism, which has been implicated in cartilage development.[82] Further work is required to determine whether both of these genes independently impact height at this locus and the biological mechanism by which this uncharacterized gene *ENSG00000267561* affects height. It is notable that height is a trait with one of the strongest signals of inbreeding depression; Clark et al.[83] found increased homozygosity to be associated with reduced height. Here, we find negative effects for rare bi-allelic variants in *LECT2* but positive effects for *ENSG00000267561* (Figures 4, S8, and S9).

Discussion

This study presents a cross-biobank catalog of homozygous and CH genotypes, enabling a systematic meta-analysis of rare recessive genetic variation across 41 phenotypes and 948,690 individuals. Our findings demonstrate the value of phasing large-scale biobanks, as inclusion of CH variants provided a 19% increase in the number of identifiable bi-allelic pLoF or pLoF|damaging_missense genotypes and enabled association analyses of additional genes that would have remained unexplored when considering homozygous variants alone. The meta-analysis successfully validated several established recessive genes while identifying additional recessive associations, such as those between *ENSG00000267561* and height and between *HBB* and heart failure, lipids, and BMI.

We systematically examined recessive gene-based effects across both multiple biobanks and a broad range of phenotypes, expanding the work of Lassen et al.[5] in several ways. First, we recapitulate most associations with increased power, owing to using roughly five times more samples than the original study. In particular, our meta-analysis increased the median χ^2 values by 26% over Lassen et al. for associations with $p_{rec} < 1.0 \times 10^{-5}$ in both studies. Second, although we consider a subset of the binary phenotypes analyzed by Lassen et al., we also analyzed an additional two disease traits (maternal hemorrhage and varicose veins) and nine quantitative traits (Tables S1 and S2). In addition to the increased sample size, this meta-analysis adds value by bringing in samples from non-European ancestry groups to boost discovery: seven of our 17 significant recessive associations (*ODAD1* [MIM: 615038], plus six involving *HBB*) were driven by AFR/SAS cohorts, with no signal in EUR cohorts where the relevant variants are absent or at very low frequency.

While meta-analysis generally improved statistical power, yielding 11 associations that were not significant in any individual biobank (Figure 3B), the extent of improvement varied across gene-trait pairs, with some associations showing more significant signals in the UKB alone. This does not imply that the associations identified in UKB are false positives. Rather, it likely indicates that the sample sizes and/or case prevalence in other biobanks are too low to detect effects with the same magnitude as in UKB or reflects the heterogeneity of phenotype definitions across biobanks.[58] Future work could explore meta-analysis models that account for heterogeneity in effect sizes across cohorts, such as random-effects frameworks, to improve power while accommodating cohort-specific variation.

Of the 17 significant (FDR < 0.01) and likely recessive associations in Table 2, two findings are likely attributable to misdiagnosis or secondary effects of known recessive Mendelian conditions. The first involves COPD and *ODAD1*,[65,66] in which recessive variants cause primary ciliary dyskinesia (MIM: 615067), a lung disease with similar respiratory symptoms but distinct pathophysiology. The second is between bi-allelic variation in *PYGM* ($p_{rec} = 8.2 \times 10^{-10}$; $p_{add} = 0.6$) and AST. *PYGM* is implicated in glycogen storage disease V (MIM: 232600) (commonly known as McArdle disease), a recessive disorder of glycogen metabolism in muscle that can lead to elevated AST due to muscle damage.[73] Re-analysis after excluding all ClinVar[84] pathogenic/likely-pathogenic alleles caused 12 of our recessive signals to disappear, highlighting that most of the observed associations are driven by known monogenic disease variants (Notes S6 and S8; Figure S11). Evidently, population biobanks at this scale can recapitulate recessive Mendelian disease signals without clinical cohort ascertainment, using linked health-record phenotypes. These cases also illustrate how apparent genetic associations with common conditions may in fact represent diverse clinical manifestations of rare recessive disorders and underscore the ability of our approach to capture meaningful genetic signals. Notably,

the corresponding ORs can range from large to modest depending on annotation mask and phenotype definition: broader masks that include less severe or heterogeneous alleles can dilute effects (e.g., *ODAD1*: COPD pLoF OR = 13.99 versus nonsynonymous OR = 1.10), and proxy EHR phenotypes may further attenuate associations.

Our study has several limitations. First, our approach to determining which associations are most likely recessive is sub-optimal (Note S7; Figure S10). Specifically, comparing recessive versus additive p values assumes equally powered analyses for each locus, which is likely not the case. This problem is exaggerated in the context of meta-analysis, as power may also deviate within and between multiple studies due to differences in case ascertainment and variant allele frequencies. Second, due to limited resources, we did not phase any AOU subcohort and only considered homozygotes; misspecifying the total bi-allelic burden undoubtedly reduced power. To quantify the associated loss in power, we performed an analysis only with homozygous genotypes for our 17 recessive associations and estimated that incorporating compound heterozygotes increased power by a median of 13.8% in terms of χ^2 statistics (Table S10). Third, in the current fixed-effects meta-analysis, we combine p values across biobanks, weighting each one by the corresponding effective sample size (N_{eff}). An important assumption here is that N_{eff}, used during meta-analysis with Stouffer's method, is derived assuming all individuals are unrelated, which might not hold for biobanks with high parental relatedness, such as G&H, therefore assigning larger weights. A future analysis should therefore consider more accurate estimation of sample size in a way that accounts for the relatedness structure in the population, prior to meta-analysis. Finally, because our recessive burden tests included variants with relatively high frequency (MAF < 5%), some of the top associations may reflect tagging of nearby common variants and so could be refined by conditioning on high-frequency variants in the region.

Despite these limitations, our study demonstrates the power of large-scale, gene-based recessive association studies enabled by cross-biobank collaboration and statistical phasing. By integrating diverse cohorts and leveraging both homozygous and CH genotypes, we offer insights into the contribution of rare and low-frequency coding variants to complex traits. This work provides a foundation for future recessive studies as sequencing becomes more widespread and data-sharing frameworks mature.

Data and code availability

Summary statistics from individual cohorts and meta-analysis are available at https://doi.org/10.5281/zenodo.16312669. The pipeline we developed for phasing is available at https://github.com/BRaVa-genetics/snakemake_pipeline_for_phasing. Scripts for other parts of our analysis are available at https://github.com/BRaVa-genetics/brava_recessive_analysis. Research on the de-identified patient data used in this publication can be carried out in the Genomics England Research Environment subject to a collaborative agreement that adheres to patient-led governance. All interested readers will be able to access the data in the same manner that the authors accessed the data. For more information about accessing the data, interested readers may contact research-network@genomicsengland.co.uk or access the relevant information on the Genomics England website: https://www.genomicsengland.co.uk/research.

Consortia

The members of the BBJ project are Koichi Matsuda, Yuji Yamanashi, Yoichi Furukawa, Takayuki Morisaki, Yukinori Okada, Yoshinori Murakami, Yoichiro Kamatani, Kaori Muto, Akiko Nagai, Yusuke Nakamura, Wataru Obara, Ken Yamaji, Kazuhisa Takahashi, Satoshi Asai, Yasuo Takahashi, Shinichi Higashiue, Shuzo Kobayashi, Hiroki Yamaguchi, Yasunobu Nagata, Satoshi Wakita, Yasushi Okazaki, Naoyuki Matsumoto, Chikako Nito, Yu-ki Iwasaki, Shigeo Murayama, Kozo Yoshimori, Yoshio Miki, Daisuke Obata, Masahiko Higashiyama, Kenta Motomura, Hidenobu Koga, and Yukihiro Koretsune.

The members of the G&H research team are Eamonn Maher, Shabana Chaudhary, Joseph Gafton, Karen A. Hunt, Shapna Hussain, Kamrul Islam, Mohammed Bodrul Mazid, Elizabeth Owor, Jessry Russell, Nishat Safa, John Solly, Marie Spreckley, David A. Van Heel, Jan Whalley, Ishevanhu Zengeya, Emily Mantle, Shaheen Akhtar, Samina Ashraf, Dan Mason, John Wright, Daniel MacArthur, Michael Simpson, Richard C. Trembath, Gerome Breen, Raymond Chung, Sang Hyuck Lee, Omar Asgar, Joanne Harvey, Karen Tricker, Caroline Winckley, Hanifa Khatun, Amna Asif, Claudia Langenberg, Grainne Colligan, Ceri Durham, Bill Newman, Ahsan Khan, Hilary Martin, Teng Heng, Matt Hurles, Vivek Iyer, Georgios Kalantzis, Vladimir Ovchinnikov, Iaroslav Popov, Klaudia Walter, Panos Deloukas, David Collier, Ana Angel, Saeed Bidi, Fabiola Eto, Sarah Finer, Chris Griffiths, Sam Hodgson, Benjamin M Jacobs, Rohini Mathur, Caroline Morton, Asma Qureshi, Stuart Rison, Annum Salman, Miriam Samuel, Moneeza K. Siddiqui, Daniel Stow, Sabina Yasmin, Julia Zöllner, and Sheik Dowlut.

The members of the BRaVa consortium are Nathalie Chami, Ron Do, Karol Estrada, Sarah Finer, Jeremy Guez, Henrike Heyne, Barney Hill, Sam Hodgson, Yuval Itan, Maarja Jõeloo, Georgios Kalantzis, Masahiro Kanai, Konrad J. Karzcewski, Athanasios Kousathanas, Satoshi Koyama, Frederik H. Lassen, Cecilia M. Lindgren, Ruth J.F. Loos, Wenhan Lu, Hilary Martin, Loukas Moutsianas, Shinichi Namba, Pradeep Natarajan, Benjamin M. Neale, Yukinori Okada, Duncan S. Palmer, Gina M. Peloso, Palta Priit, Augusto Rendon, Ghislain Rocheleau, Zachary B. Rodriguez, Omid Sadeghi-Alavijeh, Margaret Sunitha Selvaraj, Jonathan A. Shortt, Roelof A.J. Smit, Kyuto Sonehara, David van Heel, Nicholas Vartanian, Anurag Verma, Ha My T. Vy, Isaac A. Wade, Dapeng Wang, Zhi Yu, and Wei Zhou.

Acknowledgments

We thank the Human Genetics Informatics group at the Wellcome Sanger Institute for code development support and preparation of the G&H whole-exome sequencing data. We thank Klaudia Walter, Nikolas Baya, Kate Burley, Laura Fachal, Athanasios Kousathanas, and Dongjing Liu for useful discussions and Simone Rubinacci for SHAPEIT5 support.

Research was conducted using UKB (application 11867). We gratefully acknowledge AOU participants for their contributions, without whom this research would not have been possible. We thank the NIH's AOU Research Program for making available the participant cohorts examined in this study. F.H.L. was supported by the Wellcome Trust (224894/Z/21/Z) and Medical Sciences Doctoral Training Centre, University of Oxford. C.M.L. was supported by the Li Ka Shing Foundation, NIHR Oxford Biomedical Research Centre, NIH (1P50HD104224-01), Gates Foundation (INV-024200), and Wellcome Trust Investigator Award (WTIA) (221782/Z/20/Z). D.S.P is supported by a WTIA (221782/Z/20/Z) and Pioneer Centre for SMARTbiomed. This research was funded in part by Wellcome (grant no. 220540/Z/20/A, "Wellcome Sanger Institute Quinquennial Review 2021–2026"; to G.K. and H.C.M.) and the German Research Foundation (DFG; 516649954 to H.O.H.). W.Z. was supported by NHGRI (K99/R00HG012222). We acknowledge the Pioneer Center for Statistical and Computational Methods for Advanced Research to Transform Biomedicine (SMARTbiomed), DNRF grant number P4. For open access, the authors have applied a CC-BY license. Additional acknowledgments are provided in Note S9.

Declaration of interests

F.H.L. is a director and shareholder at Omos Biosciences Ltd. but conducted this work as a student at the University of Oxford. C.M.L. owns equity in Population Health Partners and its subsidiaries, reports grants from Bayer AG and Novo Nordisk, and has a partner who works at Ochre Bio. B.M.N. is a member of the scientific advisory board at Deep Genomics and Neumora Therapeutics, Inc.

Supplemental information

Supplemental information can be found online at https://doi.org/10.1016/j.ajhg.2026.04.005.

Received: September 15, 2025
Accepted: April 8, 2026
Published: May 1, 2026

References

1. Minikel, E.V., Karczewski, K.J., Martin, H.C., Cummings, B.B., Whiffin, N., Rhodes, D., Alföldi, J., Trembath, R.C., van Heel, D.A., Daly, M.J., et al. (2020). Evaluating drug targets through human loss-of-function genetic variation. Nature *581*, 459–464. https://doi.org/10.1038/s41586-020-2267-z.
2. Blom, D.J., Hala, T., Bolognese, M., Lillestol, M.J., Toth, P.D., Burgess, L., Ceska, R., Roth, E., Koren, M.J., Ballantyne, C.M., et al. (2014). A 52-week placebo-controlled trial of evolocumab in hyperlipidemia. N. Engl. J. Med. *370*, 1809–1819. https://doi.org/10.1056/NEJMoa1316222.
3. McGregor, T.L., Hunt, K.A., Yee, E., Mason, D., Nioi, P., Ticau, S., Pelosi, M., Loken, P.R., Finer, S., Lawlor, D.A., et al. (2020). Characterising a healthy adult with a rare HAO1 knockout to support a therapeutic strategy for primary hyperoxaluria. eLife *9*, e54363. https://doi.org/10.7554/eLife.54363.
4. Mackinnon, S.R., Zarganes-Tzitzikas, T., Adams, C.J., Brennan, P.E., and Yue, W.W. (2025). Luminescence-based complementation assay to assess target engagement and cell permeability of glycolate oxidase (HAO1) inhibitors. Biochimie *228*, 71–81. https://doi.org/10.1016/j.biochi.2024.08.011.
5. Lassen, F.H., Venkatesh, S.S., Baya, N., Hill, B., Zhou, W., Bloemendal, A., Neale, B.M., Kessler, B.M., Whiffin, N., Lindgren, C.M., and Palmer, D.S. (2024). Exome-wide evidence of compound heterozygous effects across common phenotypes in the UK Biobank. Cell Genom. *4*, 100602. https://doi.org/10.1016/j.xgen.2024.100602.
6. Heng, T.H., Walter, K., Huang, Q.Q., Karjalainen, J., Daly, M.J., Heyne, H.O., Malawsky, D.S., Kalantzis, G., and Martin, H.C. (2025). Widespread recessive effects on common diseases in a cohort of 44,000 British Pakistanis and Bangladeshis with high autozygosity. Am. J. Hum. Genet. *112*, 1316–1329. https://doi.org/10.1016/j.ajhg.2025.03.020.
7. Koyama, S., Yu, Z., Choi, S.H., Jurgens, S.J., Selvaraj, M.S., Klarin, D., Huffman, J.E., Clarke, S.L., Trinh, M.N., Ravi, A., et al. (2024). Exome wide association study for blood lipids in 1,158,017 individuals from diverse populations. Preprint at medRxiv. https://doi.org/10.1101/2024.09.17.24313718.
8. Guindo-Martínez, M., Amela, R., Bonàs-Guarch, S., Puiggròs, M., Salvoro, C., Miguel-Escalada, I., Carey, C.E., Cole, J.B., Rüeger, S., Atkinson, E., et al. (2021). The impact of non-additive genetic associations on age-related complex diseases. Nat. Commun. *12*, 2436. https://doi.org/10.1038/s41467-021-21952-4.
9. All of Us Research Program Genomics Investigators (2024). Genomic data in the All of Us Research Program. Nature *627*, 340–346. https://doi.org/10.1038/s41586-023-06957-x.
10. Akiyama, M., Okada, Y., Kanai, M., Takahashi, A., Momozawa, Y., Ikeda, M., Iwata, N., Ikegawa, S., Hirata, M., Matsuda, K., et al. (2017). Genome-wide association study identifies 112 new loci for body mass index in the Japanese population. Nat. Genet. *49*, 1458–1467. https://doi.org/10.1038/ng.3951.
11. Akiyama, M., Ishigaki, K., Sakaue, S., Momozawa, Y., Horikoshi, M., Hirata, M., Matsuda, K., Ikegawa, S., Takahashi, A., Kanai, M., et al. (2019). Characterizing rare and low-frequency height-associated variants in the Japanese population. Nat. Commun. *10*, 4393. https://doi.org/10.1038/s41467-019-12276-5.
12. Kanai, M., Akiyama, M., Takahashi, A., Matoba, N., Momozawa, Y., Ikeda, M., Iwata, N., Ikegawa, S., Hirata, M., Matsuda, K., et al. (2018). Genetic analysis of quantitative traits in the Japanese population links cell types to complex human diseases. Nat. Genet. *50*, 390–400. https://doi.org/10.1038/s41588-018-0047-6.
13. Sakaue, S., Kanai, M., Tanigawa, Y., Karjalainen, J., Kurki, M., Koshiba, S., Narita, A., Konuma, T., Yamamoto, K., Akiyama, M., et al. (2021). A cross-population atlas of genetic associations for 220 human phenotypes. Nat. Genet. *53*, 1415–1424. https://doi.org/10.1038/s41588-021-00931-x.
14. Hirata, M., Nagai, A., Kamatani, Y., Ninomiya, T., Tamakoshi, A., Yamagata, Z., Kubo, M., Muto, K., Kiyohara, Y.,

Mushiroda, T., et al. (2017). Overview of BioBank Japan follow-up data in 32 diseases. J. Epidemiol. *27*, S22–S28. https://doi.org/10.1016/j.je.2016.12.006.
15. Belbin, G.M., Cullina, S., Wenric, S., Soper, E.R., Glicksberg, B.S., Torre, D., Moscati, A., Wojcik, G.L., Shemirani, R., Beckmann, N.D., et al. (2021). Toward a fine-scale population health monitoring system. Cell *184*, 2068–2083.e11. https://doi.org/10.1016/j.cell.2021.03.034.
16. Finer, S., Martin, H.C., Khan, A., Hunt, K.A., MacLaughlin, B., Ahmed, Z., Ashcroft, R., Durham, C., MacArthur, D.G., McCarthy, M.I., et al. (2020). Cohort Profile: East London Genes & Health (ELGH), a community-based population genomics and health study in British Bangladeshi and British Pakistani people. Int. J. Epidemiol. *49*, 20–21i. https://doi.org/10.1093/ije/dyz174.
17. Malawsky, D.S., van Walree, E., Jacobs, B.M., Heng, T.H., Huang, Q.Q., Sabir, A.H., Rahman, S., Sharif, S.M., Khan, A., Mirkov, M.U., et al. (2023). Influence of autozygosity on common disease risk across the phenotypic spectrum. Cell *186*, 4514–4527.e14. https://doi.org/10.1016/j.cell.2023.08.028.
18. Kim, H.I., DeBoever, C., Walter, K., Kalantzis, G., Li, C., Mozaffari, S.V., Kundu, K., Jacobs, B.M., Mohammadi-Shemirani, P., Musolf, A.M., et al. (2026). Exome sequencing and analysis of 44,028 British South Asians enriched for high autozygosity. Nat. Genet. *58*, 821–830. https://doi.org/10.1038/s41588-026-02553-7.
19. Turnbull, C., Scott, R.H., Thomas, E., Jones, L., Murugaesu, N., Pretty, F.B., Halai, D., Baple, E., Craig, C., Hamblin, A., et al. (2018). The 100 000 Genomes Project: bringing whole genome sequencing to the NHS. BMJ *361*, k1687. https://doi.org/10.1136/bmj.k1687.
20. Caulfield, M., Davies, J., Dennys, M., Elbahy, L., Fowler, T., Hill, S., Hubbard, T., Jostins, L., Maltby, N., Mahon-Pearson, J., et al. (2020). National Genomic Research Library. (figshare). https://doi.org/10.6084/M9.FIGSHARE.4530893.V7
21. Shi, S., Rubinacci, S., Hu, S., Moutsianas, L., Stuckey, A., Need, A.C., Palamara, P.F., Caulfield, M., Marchini, J., and Myers, S. (2024). A Genomics England haplotype reference panel and imputation of UK Biobank. Nat. Genet. *56*, 1800–1803. https://doi.org/10.1038/s41588-024-01868-7.
22. Bycroft, C., Freeman, C., Petkova, D., Band, G., Elliott, L.T., Sharp, K., Motyer, A., Vukcevic, D., Delaneau, O., O'Connell, J., et al. (2018). The UK Biobank resource with deep phenotyping and genomic data. Nature *562*, 203–209. https://doi.org/10.1038/s41586-018-0579-z.
23. McKenna, A., Hanna, M., Banks, E., Sivachenko, A., Cibulskis, K., Kernytsky, A., Garimella, K., Altshuler, D., Gabriel, S., Daly, M., and DePristo, M.A. (2010). The Genome Analysis Toolkit: a MapReduce framework for analyzing next-generation DNA sequencing data. Genome Res. *20*, 1297–1303. https://doi.org/10.1101/gr.107524.110.
24. Hail Team. "Hail: Cloud-native genomic dataframes and batch computing" https://github.com/hail-is/hail (2025)
25. Li, H. (2013). Aligning sequence reads, clone sequences and assembly contigs with BWA-MEM. Preprint at arXiv. https://doi.org/10.48550/arXiv.1303.3997.
26. Broad, Institute. (2025). Picard: A set of command line tools for manipulating high-throughput sequencing data and formats. http://broadinstitute.github.io/picard.
27. Jun, G., Flickinger, M., Hetrick, K.N., Romm, J.M., Doheny, K.F., Abecasis, G.R., Boehnke, M., and Kang, H.M. (2012). Detecting and estimating contamination of human DNA samples in sequencing and array-based genotype data. Am. J. Hum. Genet. *91*, 839–848. https://doi.org/10.1016/j.ajhg.2012.09.004.
28. 1000 Genomes Project Consortium, Auton, A., Brooks, L.D., Durbin, R.M., Garrison, E.P., Kang, H.M., Korbel, J.O., Marchini, J.L., McCarthy, S., McVean, G.A., and Abecasis, G.R. (2015). A global reference for human genetic variation. Nature *526*, 68–74. https://doi.org/10.1038/nature15393.
29. McLaren, W., Gil, L., Hunt, S.E., Riat, H.S., Ritchie, G.R.S., Thormann, A., Flicek, P., and Cunningham, F. (2016). The ensembl variant effect predictor. Genome Biol. *17*, 122. https://doi.org/10.1186/s13059-016-0974-4.
30. Karczewski, K.J., Francioli, L.C., Tiao, G., Cummings, B.B., Alföldi, J., Wang, Q., Collins, R.L., Laricchia, K.M., Ganna, A., Birnbaum, D.P., et al. (2020). The mutational constraint spectrum quantified from variation in 141,456 humans. Nature *581*, 434–443. https://doi.org/10.1038/s41586-020-2308-7.
31. Rentzsch, P., Witten, D., Cooper, G.M., Shendure, J., and Kircher, M. (2019). CADD: predicting the deleteriousness of variants throughout the human genome. Nucleic Acids Res. *47*, D886–D894. https://doi.org/10.1093/nar/gky1016.
32. Ioannidis, N.M., Rothstein, J.H., Pejaver, V., Middha, S., McDonnell, S.K., Baheti, S., Musolf, A., Li, Q., Holzinger, E., Karyadi, D., et al. (2016). REVEL: An ensemble method for predicting the pathogenicity of rare missense variants. Am. J. Hum. Genet. *99*, 877–885. https://doi.org/10.1016/j.ajhg.2016.08.016.
33. Liu, X., Li, C., Mou, C., Dong, Y., and Tu, Y. (2020). dbNSFP v4: a comprehensive database of transcript-specific functional predictions and annotations for human nonsynonymous and splice-site SNVs. Genome Med. *12*, 103. https://doi.org/10.1186/s13073-020-00803-9.
34. Jaganathan, K., Kyriazopoulou Panagiotopoulou, S., McRae, J.F., Darbandi, S.F., Knowles, D., Li, Y.I., Kosmicki, J.A., Arbelaez, J., Cui, W., Schwartz, G.B., et al. (2019). Predicting splicing from primary sequence with deep learning. Cell *176*, 535–548.e24. https://doi.org/10.1016/j.cell.2018.12.015.
35. Frankish, A., Carbonell-Sala, S., Diekhans, M., Jungreis, I., Loveland, J.E., Mudge, J.M., Sisu, C., Wright, J.C., Arnan, C., Barnes, I., et al. (2023). GENCODE: reference annotation for the human and mouse genomes in 2023. Nucleic Acids Res. *51*, D942–D949. https://doi.org/10.1093/nar/gkac1071.
36. Hofmeister, R.J., Ribeiro, D.M., Rubinacci, S., and Delaneau, O. (2023). Accurate rare variant phasing of whole-genome and whole-exome sequencing data in the UK Biobank. Nat. Genet. *55*, 1243–1249. https://doi.org/10.1038/s41588-023-01415-w.
37. Mölder, F., Jablonski, K.P., Letcher, B., Hall, M.B., van Dyken, P.C., Tomkins-Tinch, C.H., Sochat, V., Forster, J., Vieira, F.G., Meesters, C., et al. (2021). Sustainable data analysis with Snakemake. F1000Res. *10*, 33. https://doi.org/10.12688/f1000research.29032.3.
38. Martin, M., Patterson, M., Garg, S., O Fischer, S., Pisanti, N., Klau, G.W., Schöenhuth, A., and Marschall, T. (2016). WhatsHap: fast and accurate read-based phasing. Preprint at bioRxiv. https://doi.org/10.1101/085050.
39. Zhou, W., Bi, W., Zhao, Z., Dey, K.K., Jagadeesh, K.A., Karczewski, K.J., Daly, M.J., Neale, B.M., and Lee, S. (2022). SAIGE-GENE+ improves the efficiency and accuracy of set-based rare variant association tests. Nat. Genet. *54*, 1466–1469. https://doi.org/10.1038/s41588-022-01178-w.

40. Mbatchou, J., Barnard, L., Backman, J., Marcketta, A., Kosmicki, J.A., Ziyatdinov, A., Benner, C., O'Dushlaine, C., Barber, M., Boutkov, B., et al. (2021). Computationally efficient whole-genome regression for quantitative and binary traits. Nat. Genet. *53*, 1097–1103. https://doi.org/10.1038/s41588-021-00870-7.
41. Zhou, W., Nielsen, J.B., Fritsche, L.G., Dey, R., Gabrielsen, M.E., Wolford, B.N., LeFaive, J., VandeHaar, P., Gagliano, S.A., Gifford, A., et al. (2018). Efficiently controlling for case-control imbalance and sample relatedness in large-scale genetic association studies. Nat. Genet. *50*, 1335–1341. https://doi.org/10.1038/s41588-018-0184-y.
42. Stouffer, S., Suchman, E., DeVinney, L.C., Star, S., and Williams, R. (1949). The American soldier: Adjustment during army life. Studies in social psychology in World War II) *1*.
43. Higgins, J.P.T., and Thompson, S.G. (2002). Quantifying heterogeneity in a meta-analysis. Stat. Med. *21*, 1539–1558. https://doi.org/10.1002/sim.1186.
44. Liu, Y., and Xie, J. (2019). Cauchy combination test: a powerful test with analytic p-value calculation under arbitrary dependency structures. J. Am. Stat. Assoc. *115*, 393–402. https://doi.org/10.1080/01621459.2018.1554485.
45. Jurgens, S.J., Wang, X., Choi, S.H., Weng, L.-C., Koyama, S., Pirruccello, J.P., Nguyen, T., Smadbeck, P., Jang, D., Chaffin, M., et al. (2024). Rare coding variant analysis for human diseases across biobanks and ancestries. Nat. Genet. *56*, 1811–1820. https://doi.org/10.1038/s41588-024-01894-5.
46. Liu, Y., Chen, S., Li, Z., Morrison, A.C., Boerwinkle, E., and Lin, X. (2019). ACAT: A fast and powerful p value combination method for rare-variant analysis in sequencing studies. Am. J. Hum. Genet. *104*, 410–421. https://doi.org/10.1016/j.ajhg.2019.01.002.
47. Hamosh, A., Scott, A.F., Amberger, J.S., Bocchini, C.A., and McKusick, V.A. (2005). Online Mendelian Inheritance in Man (OMIM), a knowledgebase of human genes and genetic disorders. Nucleic Acids Res. *33*, D514–D517. https://doi.org/10.1093/nar/gki033.
48. Shuey, M.M., Stead, W.W., Aka, I., Barnado, A.L., Bastarache, J.A., Brokamp, E., Campbell, M., Carroll, R.J., Goldstein, J.A., Lewis, A., et al. (2023). Next-generation phenotyping: introducing phecodeX for enhanced discovery research in medical phenomics. Bioinformatics *39*, btad655. https://doi.org/10.1093/bioinformatics/btad655.
49. Kichaev, G., Bhatia, G., Loh, P.-R., Gazal, S., Burch, K., Freund, M.K., Schoech, A., Pasaniuc, B., and Price, A.L. (2019). Leveraging polygenic functional enrichment to improve GWAS power. Am. J. Hum. Genet. *104*, 65–75. https://doi.org/10.1016/j.ajhg.2018.11.008.
50. Heyne, H.O., Karjalainen, J., Karczewski, K.J., Lemmelä, S.M., Zhou, W., Palotie, A., Daly, M.J., Havulinna, A.S., Kurki, M., and Rehm, H.L. (2023). Mono- and bi-allelic variant effects on disease at biobank scale. Nature *613*, 519–525. https://doi.org/10.1038/s41586-022-05420-7.
51. Sulem, P., Helgason, H., Oddson, A., Stefansson, H., Gudjonsson, S.A., Zink, F., Hjartarson, E., Sigurdsson, G.T., Jonasdottir, A., Jonasdottir, A., et al. (2015). Identification of a large set of rare complete human knockouts. en. Nat. Genet. *47*, 448–452. https://doi.org/10.1038/ng.3243.
52. Narasimhan, V.M., Hunt, K.A., Mason, D., Baker, C.L., Karczewski, K.J., Barnes, M.R., Barnett, A.H., Bates, C., Bellary, S., Bockett, N.A., et al. (2016). Health and population effects of rare gene knockouts in adult humans with related parents. Science *352*, 474–477. https://doi.org/10.1126/science.aac8624.
53. Oddsson, A., Sulem, P., Sveinbjornsson, G., Arnadottir, G.A., Steinthorsdottir, V., Halldorsson, G.H., Atlason, B.A., Oskarsson, G.R., Helgason, H., Nielsen, H.S., et al. (2023). Deficit of homozygosity among 1.52 million individuals and genetic causes of recessive lethality. Nat. Commun. *14*, 3453. https://doi.org/10.1038/s41467-023-38951-2.
54. Saleheen, D., Natarajan, P., Armean, I.M., Zhao, W., Rasheed, A., Khetarpal, S.A., Won, H.-H., Karczewski, K.J., O'Donnell-Luria, A.H., Samocha, K.E., et al. (2017). Human knockouts and phenotypic analysis in a cohort with a high rate of consanguinity. Nature *544*, 235–239. https://doi.org/10.1038/nature22034.
55. Sun, K.Y., Bai, X., Chen, S., Bao, S., Zhang, C., Kapoor, M., Backman, J., Joseph, T., Maxwell, E., Mitra, G., et al. (2024). A deep catalogue of protein-coding variation in 983,578 individuals. Nature *631*, 583–592. https://doi.org/10.1038/s41586-024-07556-0.
56. Karczewski, K.J., Solomonson, M., Chao, K.R., Goodrich, J.K., Tiao, G., Lu, W., Riley-Gillis, B.M., Tsai, E.A., Kim, H.I., Zheng, X., et al. (2022). Systematic single-variant and gene-based association testing of thousands of phenotypes in 394,841 UK Biobank exomes. Cell Genom. *2*, 100168. https://doi.org/10.1016/j.xgen.2022.100168.
57. Cerezo, M., Sollis, E., Ji, Y., Lewis, E., Abid, A., Bircan, K.O., Hall, P., Hayhurst, J., John, S., Mosaku, A., et al. (2025). The NHGRI-EBI GWAS Catalog: standards for reusability, sustainability and diversity. Nucleic Acids Res. *53*, D998–D1005. https://doi.org/10.1093/nar/gkae1070.
58. Monti, R., Eick, L., Hudjashov, G., Läll, K., Kanoni, S., Wolford, B.N., Wingfield, B., Pain, O., Wharrie, S., Jermy, B., et al. (2024). Evaluation of polygenic scoring methods in five biobanks shows larger variation between biobanks than methods and finds benefits of ensemble learning. Am. J. Hum. Genet. *111*, 1431–1447. https://doi.org/10.1016/j.ajhg.2024.06.003.
59. Theodoratou, E., Campbell, H., Tenesa, A., Houlston, R., Webb, E., Lubbe, S., Broderick, P., Gallinger, S., Croitoru, E.M., Jenkins, M.A., et al. (2010). A large-scale meta-analysis to refine colorectal cancer risk estimates associated with MUTYH variants. Br. J. Cancer *103*, 1875–1884. https://doi.org/10.1038/sj.bjc.6605966.
60. Thyssen, J.P., Godoy-Gijon, E., and Elias, P.M. (2013). Ichthyosis vulgaris: the filaggrin mutation disease: Ichthyosis vulgaris. Br. J. Dermatol. *168*, 1155–1166. https://doi.org/10.1111/bjd.12219.
61. Laurell, C.-B., and Eriksson, S. (2013). The electrophoretic α1-globulin pattern of serum in α1-antitrypsin deficiency. 1963 COPD *10*, 3–8. https://doi.org/10.3109/15412555.2013.771956.
62. Horowitz, J.E., Warner, N., Staples, J., Crowley, E., Gosalia, N., Murchie, R., Van Hout, C., Fiedler, K., Welch, G., King, A.K., et al. (2021). Mutation spectrum of NOD2 reveals recessive inheritance as a main driver of Early Onset Crohn's Disease. Sci. Rep. *11*, 5595. https://doi.org/10.1038/s41598-021-84938-8.
63. Liu, Z., Liu, R., Gao, H., Jung, S., Gao, X., Sun, R., Liu, X., Kim, Y., Lee, H.-S., Kawai, Y., et al. (2023). Genetic architecture of the inflammatory bowel diseases across East Asian and

European ancestries. Nat. Genet. *55*, 796–806. https://doi.org/10.1038/s41588-023-01384-0.
64. Sazonovs, A., Stevens, C.R., Venkataraman, G.R., Yuan, K., Avila, B., Abreu, M.T., Ahmad, T., Allez, M., Ananthakrishnan, A.N., Atzmon, G., et al. (2022). Large-scale sequencing identifies multiple genes and rare variants associated with Crohn's disease susceptibility. Nat. Genet. *54*, 1275–1283. https://doi.org/10.1038/s41588-022-01156-2.
65. Knowles, M.R., Leigh, M.W., Ostrowski, L.E., Huang, L., Carson, J.L., Hazucha, M.J., Yin, W., Berg, J.S., Davis, S.D., Dell, S.D., et al. (2013). Exome sequencing identifies mutations in CCDC114 as a cause of primary ciliary dyskinesia. Am. J. Hum. Genet. *92*, 99–106. https://doi.org/10.1016/j.ajhg.2012.11.003.
66. Onoufriadis, A., Paff, T., Antony, D., Shoemark, A., Micha, D., Kuyt, B., Schmidts, M., Petridi, S., Dankert-Roelse, J.E., Haarman, E.G., et al. (2013). Splice-site mutations in the axonemal outer dynein arm docking complex gene CCDC114 cause primary ciliary dyskinesia. Am. J. Hum. Genet. *92*, 88–98. https://doi.org/10.1016/j.ajhg.2012.11.002.
67. Crystal, R.G. (1990). Alpha 1-antitrypsin deficiency, emphysema, and liver disease. Genetic basis and strategies for therapy. J. Clin. Investig. *85*, 1343–1352. https://doi.org/10.1172/jci114578.
68. Nielsen, J.B., Rom, O., Surakka, I., Graham, S.E., Zhou, W., Roychowdhury, T., Fritsche, L.G., Gagliano Taliun, S.A., Sidore, C., Liu, Y., et al. (2020). Loss-of-function genomic variants highlight potential therapeutic targets for cardiovascular disease. Nat. Commun. *11*, 6417. https://doi.org/10.1038/s41467-020-20086-3.
69. Arnett, H.A., Escobar, S.S., and Viney, J.L. (2009). Regulation of costimulation in the era of butyrophilins. Cytokine *46*, 370–375. https://doi.org/10.1016/j.cyto.2009.03.009.
70. Reilly, N.A., Lutgens, E., Kuiper, J., Heijmans, B.T., and Jukema, J.W. (2021). Effects of fatty acids on T cell function: role in atherosclerosis. Nat. Rev. Cardiol. *18*, 824–837. https://doi.org/10.1038/s41569-021-00582-9.
71. DeWan, A.T., Cahill, M.E., Cornejo-Sanchez, D.M., Li, Y., Dong, Z., Fabiha, T., Sun, H., Wang, G., and Leal, S.M. (2023). Variants in JAZF1 are associated with asthma, type 2 diabetes, and height in the United Kingdom biobank population. Front. Genet. *14*, 1129389. https://doi.org/10.3389/fgene.2023.1129389.
72. Mountjoy, E., Schmidt, E.M., Carmona, M., Schwartzentruber, J., Peat, G., Miranda, A., Fumis, L., Hayhurst, J., Buniello, A., Karim, M.A., et al. (2021). An open approach to systematically prioritize causal variants and genes at all published human GWAS trait-associated loci. Nat. Genet. *53*, 1527–1533. https://doi.org/10.1038/s41588-021-00945-5.
73. Nogales-Gadea, G., Brull, A., Santalla, A., Andreu, A.L., Arenas, J., Martín, M.A., Lucia, A., de Luna, N., and Pinós, T. (2015). McArdle disease: Update of reported mutations and polymorphisms in the PYGM gene. Hum. Mutat. *36*, 669–678. https://doi.org/10.1002/humu.22806.
74. Yates, T.M., Ansari, M., Thompson, L., Hunt, S.E., Uhalte, E.C., Hobson, R.J., Marsh, J.A., Wright, C.F., and Firth, H.V. (2024). Curating genomic disease-gene relationships with Gene2Phenotype (G2P). Genome Med. *16*, 127. https://doi.org/10.1186/s13073-024-01398-1.
75. Carlson, J.C., Krishnan, M., Rosenthal, S.L., Russell, E.M., Zhang, J.Z., Hawley, N.L., Moors, J., Cheng, H., Dalbeth, N., de Zoysa, J.R., et al. (2023). A stop-gain variant in BTNL9 is associated with atherogenic lipid profiles. HGG Adv. *4*, 100155. https://doi.org/10.1016/j.xhgg.2022.100155.
76. Haghpanah, S., Davani, M., Samadi, B., Ashrafi, A., and Karimi, M. (2010). Serum lipid profiles in patients with beta-thalassemia major and intermedia in southern Iran. J. Res. Med. Sci. *15*, 150–154.
77. Mohamed, S.O.O., Mohamed, A.E.A., Salih, M.S.K., Salih, K.S.K., Abdelrahman, A.S.E.E., Abdelgadir, A.G.A., Ahmedkaroum, M.G.A., Abdalla, G.A., Fadil, H.A.M., Abdelrahman, M.A.M., and Salih, N.S.A. (2024). Serum lipid profile abnormalities among beta-thalassemia patients: a systematic review and meta-analysis. Lipids Health Dis. *23*, 388. https://doi.org/10.1186/s12944-024-02377-6.
78. Gujja, P., Rosing, D.R., Tripodi, D.J., and Shizukuda, Y. (2010). Iron overload cardiomyopathy: better understanding of an increasing disorder. J. Am. Coll. Cardiol. *56*, 1001–1012. https://doi.org/10.1016/j.jacc.2010.03.083.
79. Sawicki, K.T., De Jesus, A., and Ardehali, H. (2023). Iron metabolism in cardiovascular disease: Physiology, mechanisms, and therapeutic targets. Circ. Res. *132*, 379–396. https://doi.org/10.1161/CIRCRESAHA.122.321667.
80. Auvinen, J., Tapio, J., Karhunen, V., Kettunen, J., Serpi, R., Dimova, E.Y., Gill, D., Soininen, P., Tammelin, T., Mykkänen, J., et al. (2021). Systematic evaluation of the association between hemoglobin levels and metabolic profile implicates beneficial effects of hypoxia. Sci. Adv. *7*, eabi4822. https://doi.org/10.1126/sciadv.abi4822.
81. Yengo, L., Vedantam, S., Marouli, E., Sidorenko, J., Bartell, E., Sakaue, S., Graff, M., Eliasen, A.U., Jiang, Y., Raghavan, S., et al. (2022). A saturated map of common genetic variants associated with human height. Nature *610*, 704–712. https://doi.org/10.1038/s41586-022-05275-y.
82. Kang, D., Lee, J., Wu, C., Guo, X., Lee, B.J., Chun, J.-S., and Kim, J.-H. (2020). The role of selenium metabolism and selenoproteins in cartilage homeostasis and arthropathies. Exp. Mol. Med. *52*, 1198–1208. https://doi.org/10.1038/s12276-020-0408-y.
83. Clark, D.W., Okada, Y., Moore, K.H.S., Mason, D., Pirastu, N., Gandin, I., Mattsson, H., Barnes, C.L.K., Lin, K., Zhao, J.H., et al. (2019). Associations of autozygosity with a broad range of human phenotypes. Nat. Commun. *10*, 4957. https://doi.org/10.1038/s41467-019-12283-6.
84. Landrum, M.J., Lee, J.M., Benson, M., Brown, G., Chao, C., Chitipiralla, S., Gu, B., Hart, J., Hoffman, D., Hoover, J., et al. (2016). ClinVar: public archive of interpretations of clinically relevant variants. Nucleic Acids Res. *44*, D862–D868. https://doi.org/10.1093/nar/gkv1222.

The American Journal of Human Genetics, Volume *113*

Supplemental information

Meta-analysis across six global biobanks identifies recessive coding associations with complex traits and diseases

Frederik H. Lassen, Georgios Kalantzis, Andrea Eoli, Barney Hill, Kyuto Sonehara, Shinichi Namba, Isaac Wade, Sam Hodgson, Wei Zhou, BioBank Japan Project, Genes & Health Research Team, BRaVa Consortium, Benjamin M. Neale, Konrad J. Karczewski, Yukinori Okada, David A. van Heel, Sarah Finer, Cecilia M. Lindgren, Henrike O. Heyne, Hilary C. Martin, and Duncan S. Palmer

Supplemental Material and Methods

Supplemental Notes

Note S1. Phenotyping and case counts across biobanks

As a data quality check, we compared the disease burden across biobanks (Fig. S1). Most UK-based cohorts showed high correlation in phenotype prevalence, e.g. r = 0.95 for UKB-100kGP, or r = 0.75 for 100kGP-G&H. Phenotype prevalence in AOU also showed high correlation with most other biobanks e.g. 0.83 with BioMe, or 0.81 with UKB. Prevalence in all three UK-based cohorts had low correlation with BBJ (maximum Spearman correlation r = 0.44), which is potentially a reflection of the fact that the UK studies incorporate both primary and secondary care data, while BBJ is a hospital-based cohort, enriched for sick individuals, as well as having a distinct ancestry composition[1]. Nonetheless, the general high concordance suggests a robust overall approach to case identification across the consortium.

We investigated specific instances where disease incidence differed markedly across biobanks, likely reflecting both established epidemiological trends and distinct characteristics of each biobank. A striking example is the case of Type 2 Diabetes (T2D) cases. The global adult T2D prevalence is projected to reach 7.7% by 2030[2], which is significantly lower than the rates we observed in our study e.g. 25.7% in G&H, or 8.6% in UKB. This disparity aligns with the documented higher prevalence of T2D in South Asian populations[3,4] and aging individuals[5], as well as in diverse urban settings[6,7]. Further examples underscore how biobank-specific factors can significantly influence observed patterns of disease occurrence. Asthma prevalence demonstrates this clearly: while the global prevalence was around 3.3% in 2021[8], we observed markedly different rates across biobanks. BBJ had only 0.8% (100 cases) with asthma codes, far below Japan's population prevalence of 3.5%, likely because only very severe cases have these codes recorded in a hospital setting. In contrast, UKB and G&H, which had primary care as well as secondary care records, had much higher rates at 9.7% and 14.7%, respectively, whereas the UK average is 10.0%. Similarly, cancer prevalence patterns reflect biobank-specific recruitment strategies. In 100kGP, 7.3% (4,276 cases) were diagnosed with breast cancer, nearly double the 3.7% observed in UKB. This high rate aligns with 100kGP's focus on cancer patients as part of its rare disease and

cancer sequencing initiative[9,10]. These examples highlight how the design and focus of each biobank can lead to significant variations in disease prevalence.

Note S2. Preprocessing for the 100,000 Genomes Project

The following workflow is adapted from Genomics-England's documentation available online. To avoid computationally cumbersome recalling of variant calls in WGS from 100kGP, and in order to harmonise the processing perfectly with other datasets, we leveraged the quality-controlled Aggregated Variant Call (AggV2) dataset, a multi-sample variant call format (VCF) comprising 78,195 germline genomes and 722 million SNPs and small indels (≤ 50bp) upon release. This dataset was constructed by merging single-sample gVCF files, with sample and variant-level quality control described below. Samples were sequenced on Illumina HiSeq X instruments using 150bp paired-end reads, one lane per sample. Data processing employed the Illumina North Star v4 Whole Genome Sequencing Workflow (NSV4, v2.6.53.23), using iSAAC Aligner (v03.16.02.19) and Starling Small Variant Caller (v2.4.7). Reads were aligned to the Homo sapiens NCBI GRCh38 assembly with decoys. Samples were individually quality controlled and required to meet the following criteria:

- Sample contamination (freemix) < 0.03
- Ratio of single nucleotide polymorphism (SNV) heterozygous to homozygous calls
- Total number of SNVs between 3.2M-4.7M
- Array concordance > 90%
- Median fragment size > 250bp
- Excess of chimeric reads < 5%
- Percentage of mapped reads > 60%
- Percentage of AT dropout < 10%

Additionally, samples were required to have at least 95% of the genome covered at 15x or above with well mapped reads (mapping quality > 10) after discarding duplicates. Variants were split from multi-allelic into bi-allelic and indels were left-aligned using vt v0.57721. Variants were filtered based on the following criteria:

- Missingness ≤ 5%
- Median depth ≥ 10
- Median genotype quality ≥ 15
- ≥ 25% of heterozygous calls showing no significant allele imbalance
- ≥ 50% of sites with complete genotype data

- HWE mid *p*-value ≥ 10^{-5} in unrelated samples of inferred European ancestry

Finally, sex-specific QC was performed for the X chromosome. In summary, 722,342,407 (74.8%) autosomal variants passed all QC filters.

Statistical Phasing

For the 100kGP we used phased genotypes generated by Shi et al.[11] using SHAPEIT4, who performed additional variant- and sample-level quality control prior to phasing. The following description is adapted from their work. This quality control process evaluated genotype quality, depth, missingness, allelic balance, Mendelian errors, HWE equilibrium, and gnomAD[12] allele frequency concordance. Shi et al. removed singletons in unrelated individuals, as these could not be phased statistically using SHAPEIT4. Overall, these stringent filters reduced the number of variants from 722M to 342M.

The phasing strategy, as described by Shi et al., involved a multi-step strategy that exploited familial relationships within 100kGP. First, they used duos and trios to obtain phase by direct transmission in offspring. Using those phased haplotypes as scaffold, they then phased the remaining unphased genotypes in related samples using SHAPEIT4 (v4.2.2). Then, using all phases as scaffold, they phased common variants (MAF>0.01) for unrelated samples using SHAPEIT4. Finally, the remaining rare variants in unrelated samples were phased using the phased common variants as a scaffold, and any phased related samples as a reference panel. Phasing of related individuals was conducted at the chromosome level, while the latter step was carried out in regions of approximately 300,000 sites, with 30,000 sites on each side as a buffer. The resulting phases for regional segments were merged and concatenated using the ligate tool from bcftools. Shi et al. assessed phasing accuracy by evaluating the performance of 100kGP as a reference panel through genotype imputation in 589 trios from the 1000 Genomes Project, using SHAPEIT4. Phasing accuracy was then evaluated by comparing imputed haplotypes to those inferred from Mendelian inheritance patterns in each trio.

Phenotyping and additional control exclusions in 100kGP

We excluded probands in 100kGP diagnosed with either global developmental delay (n = 12,191), intellectual disability (n = 9,149), or autism (n = 4,609), identified through manual curation of ICD and Human Phenotype Ontology (HPO) codes. We refer to these terms collectively as NDD. We deemed this exclusion necessary for two reasons: firstly, NDD patients in our cohort were predominantly very young (median age 15 years), making them more likely to be classified as controls for many common adult-onset diseases investigated

here. Secondly, NDD patients are likely to be enriched for rare bi-allelic variants[13]. The combination of these factors could potentially lead to an artificial enrichment of rare bi-allelic variants in our control group, risking spurious associations where the absence of these variants might appear to increase disease risk. To mitigate this potential bias, we excluded a total of 13,976 NDD probands, reducing our sample size to 69,513.

Note S3. Assessment of phasing quality across cohorts

We evaluated phasing accuracy across multiple biobanks and ancestries (EUR, SAS, and EAS) using complementary strategies tailored to the available data (Sup. Figure 2 and Sup. Table 11).

For UKB and G&H, we leveraged 99 and 100 parent–offspring trios, respectively, to estimate switch-error rates (SER) after statistical phasing. Across all variants, the average SER was low, with 0.16% in UKB and 0.35% in G&H, indicating overall high-quality statistical phasing. As expected, rare variants showed elevated error rates: singletons (MAC = 1) exhibited the highest SER, reaching 31.77% in UKB and 24.29% in G&H, with errors decreasing steadily as MAC increased. Importantly, restricting to high-confidence phased genotypes (posterior probability, PP > 90%), which is what we used in all downstream analyses, substantially reduced the SER, with 4.12% and 5.63% for MAC = 1 in UKB and G&H respectively, providing reassurance that phasing quality for rare variants is well controlled after posterior filtering.

In BBJ, where trio data were not available, we benchmarked statistical phasing against read-backed phasing from short-read sequencing calls using WhatsHap[14]across 1000 randomly sampled individuals of EAS ancestry. We then used custom R scripts to determine the agreement between read-backed phased and statistically inferred haplotypes. This process involved identifying pairs of variants in close proximity (150 - 250bp) on short-read sequences from .bam or .cram files using WhatsHap[14] with default parameters. Two variants on the same short-sequencing read must originate from the same haplotype (in *cis*), while variants on different reads are likely to originate from opposite haplotypes (in *trans*). Given this, phasing accuracy was estimated as the proportion of read-backed configurations of variant pairs that agreed with the configuration of statistically inferred variants. Similarly to UKB and G&H, error rates were highest for singletons, at 38.26%, but decreased rapidly for low-frequency variants (e.g. 4.36% for 1<MAC<5). Applying the same PP > 90% criterion yielded markedly improved error rates, with an average of 0.30% across all variants. This demonstrates that posterior filtering effectively enriches for correctly phased haplotypes in

BBJ as well, supporting the robustness of our phasing strategy across cohorts and ancestries.

Note S4. Gene knockouts and comparison with other studies

To date, several studies have systematically identified and reported rare predicted bi-allelic LoF individuals, collectively encompassing 7,149 unique autosomal genes with knockouts (KO hereafter)[12,15–18]. Sun et al.[19], the most recent study, observed 4,848 genes in a sample of 983,578 individuals, considering pLoF variants with MAF < 1%. Following these studies, we set out to survey pLoF (HC by LOFTEE; Methods) variants in 19,334 unique human protein-coding genes across our six biobanks, to identify bi-allelic genotypes, using MAF < 1% to match the literature; this threshold is different to the one for our recessive association study for which we used a more lenient threshold of 5% to ensure sufficient numbers of bi-allelic genotypes.

While working with SHAPEIT5 for statistical phasing, we noticed that missing genotypes (if any) are imputed during the integral early steps of the program. We quantified the rate of missing-then-imputed pLoF-homozygotes and found it to be between 0.1% and 2% across cohorts. Although these rates would imply a miniscule effect in our association study – and many of these genotypes might be correctly imputed anyway – we decided to exclude these from our KO analysis to avoid reporting genes with false knockouts. Thus, for this analysis, we used the post-QC unphased data to ascertain homozygotes and the phased data for compound-heterozygotes.

We found a total of 5,563 genes harbouring KOs (Sup. Table 4) across chromosomes 1-22. Of these, 2,293 (41.2%) distinct genes had a bi-allelic pLoF in a single individual, as expected since pLoFs tend to be rare. Of the 3,270 genes observed with two or more bi-allelic individuals, the majority (n = 1,265, 38.7%) were restricted in two biobanks, whereas only 195 (6.0%) were observed in five or six biobanks. G&H yielded the highest number of unique genes with bi-allelic pLoF genotypes (2,519 genes), surpassing the whole of UKB (2,077 genes), despite a roughly x10 difference in sample size (39k vs 395k), due to the higher autozygosity[20]. In contrast, when considering specifically CH variation, UKB:EUR contributed the highest number of unique genes (827 genes), followed by 100kGP:EUR (431 genes), and then G&H (257 genes). Notably, 62.9% (1111/1767) of the novel KO genes were detected in individuals of SAS ancestry. Overall, these observations show how sample demography and ancestry composition – particularly autozygosity in consanguineous populations – profoundly shape the landscape of bi-allelic loss-of-function variation.

Note S5. Testing summary statistics for inflation

We assessed the quality of summary statistics by calculating two genomic inflation metrics - λ_{GC} and λ_{95}, calculated at the 50th and 95th percentiles of the test statistic distribution, respectively - across 41 traits, six biobanks, four variant annotation categories, and any available ancestry. λ_{95} is considered more appropriate for rare variant studies, where test statistics are often deflated due to sparse data[21]. Indeed, considering all recessive tests, the distribution of λ_{95} values was closer to one compared to that of λ_{GC} values, across all annotations (Fig. S3), making that a better choice to test for inflation. As a reference, both inflation factors had a similar distribution on additive tests where there is less sparsity; for example both had a mean value of 1.02 for nonsynonymous tests (Fig. S4-S5). Overall, 1,604 of the 1,630 (98.4%) recessive analyses across all biobanks, ancestries and annotations had $\lambda_{95} < 1.25$, indicating sufficient control of test-statistic inflation.

Next, we assessed the inflation factors after the meta-analysis and the Cauchy combination and found no indication of inflation, with a mean $\lambda_{95} = 1.00$ and mean $\lambda_{GC} = 0.93$ (Fig. S6). All phenotypes showed $\lambda_{95} < 1.07$, except height ($\lambda_{GC} = 1.15$, $\lambda_{95} = 1.20$), which was the one yielding the highest number of significant associations (eleven with $p_{rec} < 7.53 \times 10^{-7}$). This is perhaps expected given the well-documented polygenic architecture[22,23] and association with autozygosity[24].

Finally, as a negative control and an additional test of calibration, we performed a meta-analysis of synonymous variant burdens under the recessive model. Similarly to the cauchy results described above, all phenotypes showed calibrated λ_{95} for the synonymous tests with a mean of 0.99 and range [0.43, 1.17]. This analysis yielded five gene-trait associations significant at FDR < 0.01 (see Sup. Table 6 for more details), all of which showing more significant additive than recessive effects ($p_{add} < p_{rec}$). Importantly, all cases have support from recent association studies, suggesting these signals likely reflect linkage disequilibrium with known associations involving common variants, and the synonymous variant may well not be the causal SNP. For instance, we associate *CEACAM19* with LDL cholesterol ($p_{rec} = 1.65 \times 10^{-20}$; $p_{add} = 2.59 \times 10^{-234}$); 95% of the 254 bi-allelic individuals in this gene were homozygous for rs76075198, a high-frequency (MAF=2.5% in UKB:EUR) variant which was previously associated with LDLC[25]. Similarly, we associate *TOMM40* with C-reactive protein and ($p_{rec} = 5.48 \times 10^{-12}$; $p_{add} = 1.46 \times 10^{-194}$), a gene in which an intron variant rs34095326 has been reported in the GWAS Catalog. Lastly, we associate *CCHCR1* with height ($p_{rec} = 3.42 \times 10^{-8}$; $p_{add} = 5.98 \times 10^{-41}$), a gene with several intron variants in the

GWAS Catalog (e.g. rs2073717), all of which are near high-frequency synonymous variants in our cohort (e.g. rs130077).

Note S6. Significant associations that are likely to be driven by known rare recessive disorders

We found recessive associations between COPD and *ODAD1* ($p_{rec} = 4.8 \times 10^{-7}$), as well as a 100kGP-specific signal for *DNAI1* (pLoF|damaging_misense $p_{rec} = 8.98 \times 10^{-10}$) which did not reach significance after meta-analysis (Cauchy $p_{rec} = 5.26 \times 10^{-6}$). These two genes are known to be recessive causes of primary ciliary dyskinesia (MIM: 615038 and 604366;[26–28]) and we suspected that the associations with COPD might be due to misdiagnosis. We reviewed medical records in 100kGP and found that of the 17 individuals of damaging bi-allelic variants in these two genes, 11 had COPD of which 10 had also PCD. When conditioning COPD association tests on PCD status in 100kGP, the signals were attenuated ($p_{rec} > 0.02$), suggesting potential misclassification due to overlapping clinical features. Of note, all PCD cases (n=50) in 100kGP who were included as COPD cases were classified as such due to having the J47 (bronchiectasis) code, versus 956 of all 3,801 COPD (25%) cases; in contrast, 72% (n=2,719) of all COPD cases had J44 (“Other chronic obstructive pulmonary disease”) versus only 6 out of 131 (5%) PCD cases. This supports the notion that these individuals with PCD in 100kGP certainly do not have ‘typical’ COPD and may have been misdiagnosed. Thus, it is likely that recessive variants in *ODAD1* and *DNAI1* do not associate with COPD independently of their effects on PCD.

We also detected an association between levels of aspartate aminotransferase (AST) and *PYGM* ($p_{rec} = 8.17 \times 10^{-10}$), driven by individuals of European ancestry from UKB and AOU. The association was yielded by pLoF variants, whereas the signal attenuated after considering missense variants (pLoF|damaging_misense $p_{rec} = 3.62 \times 10^{-2}$; nonsynonymous $p_{rec} = 8.98 \times 10^{-2}$). Recessive variants in *PYGM* cause Glycogen Storage Disease V (commonly known as McArdle disease), a disorder of glycogen metabolism leading to muscle damage after exercise, which could result in the release of AST into the bloodstream[29]; indeed, by checking medical records we confirmed that 60% of *PYGM* bi-allelic pLoF individuals (exact numbers can not be reported due to privacy constraints in AOU) were diagnosed with glycogen storage disease (ICD:E74). When adjusting for diagnosis of this disease, we observed a notable attenuation, with the association becoming non-significant after meta-analysis ($p_{rec} = 5.52 \times 10^{-6}$; Sup. Table 8). We thus conclude that the elevation of AST likely derives from McArdle-related rhabdomyolysis rather than from primary liver pathology.

Note S7. Alternative ways to decide on recessive over additive associations

In our main analysis we classify recessive over additive effects by comparing P-values from the corresponding models, and by specifically assessing when $p_{rec} < p_{add} / 100$ (the “100x rule”). This was based on previous work by Heyne et al.[30] who performed simulations with 200k individuals and different MAF thresholds and found that, among the recessive tests that were significant ($p_{rec} < 5.0 \times 10^{-8}$), the 100x rule had a true positive rate of 1.00 to identify recessive over additive effects (i. e. almost no false negatives). Likewise, precisely zero simulated-additive effects were identified as recessive (i.e. no false positives). We thus set out to use this rule in our analysis of recessive gene burden.

As an alternative approach, we explored a more comprehensive rule by also considering effect sizes, to handle cases where the additive model might be under-powered, or when the additive effect might have a similar magnitude to the recessive one. To that end, we compared the difference in the ($\log_{10}$) P-values to the ratio of effect size estimates (Sup. Fig 10). An alternative classification of recessive versus additive associations could thus be:

- Recessive if $p_{rec} < p_{add} / 100$ AND ($beta_{rec} / beta_{add} > 1.5$ OR $p_{add} > 0.05$),
- Additive if $p_{add} < p_{rec} / 100$ AND $beta_{rec} / beta_{add} < 1.5$,
- Ambiguous otherwise.

Applying this rule to the results of our meta-analysis would result in 16 putatively recessive associations, fully overlapping with our original 17 (Table 2), except for the ambiguous case of IBD - *NOD2*, a gene known for mixed inheritance (MIM: 605956). Other ambiguous cases include putatively dominant effects, or associations with strong recessive effects but more significant additive P-values, such as *FYCO1* - AST ($\beta_{rec} = 0.28$; $\beta_{add} = 0.10$) or *ABHD15* - Height ($\beta_{rec} = 0.27$; $\beta_{add} = 0.07$); see Sup. Table 5 for detailed estimates. The effect-size ratio threshold of 1.5 was chosen as a conservative margin to try to distinguish true recessive effects where the additive effect estimate is inflated or the recessive effect estimate is deflated due to sampling error, but similar thresholds would yield roughly the same classification, particularly for recessive ones.

Note S8. Refining association signals without P/LP variants

To help answer whether our recessive associations are due to variants with well-established recessive effects, or due to novel effects, we repeated association testing after using information from ClinVar[31]. Specifically, we extracted any variant being pathogenic (P) or

likely pathogenic (LP) in ClinVar (November 2025 release) across the genes involved in our recessive associations (Table 2), and matched these with all variants observed in our data based on the same reference:alternate allele. We then identified any individuals being homozygotes for one, or CH for two of such P/LP variants, and devised new recessive burdens to test for association, focusing on genes showing nominally-significant results ($P < 0.05$) in the first round of analysis at AOU, G&H and 100kGP.

The new set of gene burdens were drastically sparser than the original one, in many cases resulting in no genotypes at all, though *BTNL9, LECT2* and ENSG00000267561 showed no change, as expected given that ClinVar does not contain any P/LP variants for these genes (except for CNVs which we do not consider in our study). The reduction in genotypes for the rest of genes resulted in a marked attenuation of P-values which, in most cases, resulted in a complete loss of signal (Fig. S11). To conclude, our association signals with *FLG, HBB, SERPINA1, MUTYH, PYGM,* and *ODAD1*, several of which are novel, can be attributed to established monogenic alleles whose pleiotropic effects extend to the complex traits analysed here.

Note S9. Supplemental Acknowledgements

Genes & Health is/has recently been core-funded by Wellcome (WT102627, WT210561), the Medical Research Council (UK) (M009017, MR/X009777/1, MR/X009920/1), Higher Education Funding Council for England Catalyst, Barts Charity (845/1796), Health Data Research UK (for London substantive site), and research delivery support from the NHS National Institute for Health Research Clinical Research Network (North Thames). We acknowledge the support of the National Institute for Health and Care Research Barts Biomedical Research Centre (NIHR203330); a delivery partnership of Barts Health NHS Trust, Queen Mary University of London, St George's University Hospitals NHS Foundation Trust and St George's University of London Genes & Health is/has recently been funded by Alnylam Pharmaceuticals, Genomics PLC; and a Life Sciences Industry Consortium of AstraZeneca PLC, Bristol-Myers Squibb Company, GlaxoSmithKline Research and Development Limited, Maze Therapeutics Inc, Merck Sharp & Dohme LLC, Novo Nordisk A/S, Pfizer Inc, Takeda Development Centre Americas Inc. We thank Social Action for Health, Centre of The Cell, members of our Community Advisory Group, and staff who have recruited and collected data from volunteers. We thank the NIHR National Biosample Centre (UK Biocentre), the Social Genetic & Developmental Psychiatry Centre (King's College London), Wellcome Sanger Institute, and Broad Institute for sample processing, genotyping, sequencing and variant annotation. This work uses data provided by patients and collected by the NHS as part of their care and support. This research utilised Queen Mary University of London's Apocrita HPC facility, supported by QMUL Research-IT, http://doi.org/10.5281/zenodo.438045. We thank: Barts Health NHS Trust, NHS Clinical Commissioning Groups (City and Hackney, Waltham Forest, Tower Hamlets, Newham, Redbridge, Havering, Barking and Dagenham), East London NHS Foundation Trust, Bradford Teaching Hospitals NHS Foundation Trust, Public Health England (especially David Wyllie), Discovery Data Service/Endeavour Health Charitable Trust (especially David Stables), Voror Health Technologies Ltd (especially Sophie Don), NHS England (for what was NHS Digital) - for GDPR-compliant data sharing backed by individual written informed consent. Most of all we thank all of the volunteers participating in Genes & Health.

This research was made possible through access to data in the National Genomic Research Library, which is managed by Genomics England Limited (a wholly owned company of the Department of Health and Social Care). The National Genomic Research Library holds data provided by patients and collected by the NHS as part of their care and data collected as part of their participation in research. The National Genomic Research Library is funded by

the National Institute for Health Research and NHS England. The Wellcome Trust, Cancer Research UK and the Medical Research Council have also funded research infrastructure.

We gratefully acknowledge *All of Us* participants for their contributions, without whom this research would not have been possible. We also thank the National Institutes of Health's All of Us Research Program for making available the participant data [and/or samples and/or cohort] examined in this study. The *All of Us* Research Program is supported by the National Institutes of Health, Office of the Director: Regional Medical Centers: 1 OT2 OD026549; 1 OT2 OD026554; 1 OT2 OD026557; 1 OT2 OD026556; 1 OT2 OD026550; 1 OT2 OD 026552; 1 OT2 OD026553; 1 OT2 OD026548; 1 OT2 OD026551; 1 OT2 OD026555; IAA #: AOD 16037; Federally Qualified Health Centers: HHSN 263201600085U; Data and Research Center: 5 U2C OD023196; Biobank: 1 U24 OD023121; The Participant Center: U24 OD023176; Participant Technology Systems Center: 1 U24 OD023163; Communications and Engagement: 3 OT2 OD023205; 3 OT2 OD023206; and Community Partners: 1 OT2 OD025277; 3 OT2 OD025315; 1 OT2 OD025337; 1 OT2 OD025276.

This work was supported in part by Google Cloud Research Credits provided by Google.

This work was supported in part through the computational and data resources and staff expertise provided by Scientific Computing and Data at the Icahn School of Medicine at Mount Sinai and supported by the Clinical and Translational Science Awards (CTSA) grant UL1TR004419 from the National Center for Advancing Translational Sciences. The Mount Sinai BioMe Biobank has been supported by The Andrea and Charles Bronfman Philanthropies and in part by Federal funds from the NHLBI and NHGRI (U01HG00638001; U01HG007417; X01HL134588). We thank all participants in the Mount Sinai BioMe Biobank. We also thank all of our recruiters who have assisted in data collection and management, and we are grateful for the computational resources and staff expertise provided by Scientific Computing at the Icahn School of Medicine at Mount Sinai.

We thank all participants of BioBank Japan. S.N. was supported by AMED (JP25tm0424228, JP24tm0524009) and Japan Foundation for Applied Enzymology. Y.O. was supported by JSPS KAKENHI (25H01057), and AMED (JP24km0405217, JP24ek0109594, JP24ek0410113, JP24kk0305022, JP243fa627002, JP243fa627010, JP243fa627011, JP24zf0127008, JP24tm0524002, JP24wm0625504, JP24gm1810011), JST Moonshot R&D (JPMJMS2021, JPMJMS2024), Takeda Science Foundation, Ono Pharmaceutical Foundation for Oncology, Immunology, and Neurology, Bioinformatics Initiative of Osaka University Graduate School of Medicine, Institute for Open and Transdisciplinary Research

Initiatives, Center for Infectious Disease Education and Research (CiDER), and Center for Advanced Modality and DDS (CAMaD), Osaka University, RIKEN TRIP initiative (AGIS).
During the preparation of this work the authors used *ChatGPT* and *Claude* in order to simplify handling of results files and improve clarity in the manuscript. After using this tool/service, the authors reviewed and edited the content as needed and take full responsibility for the content of the published article.

Supplemental Figures

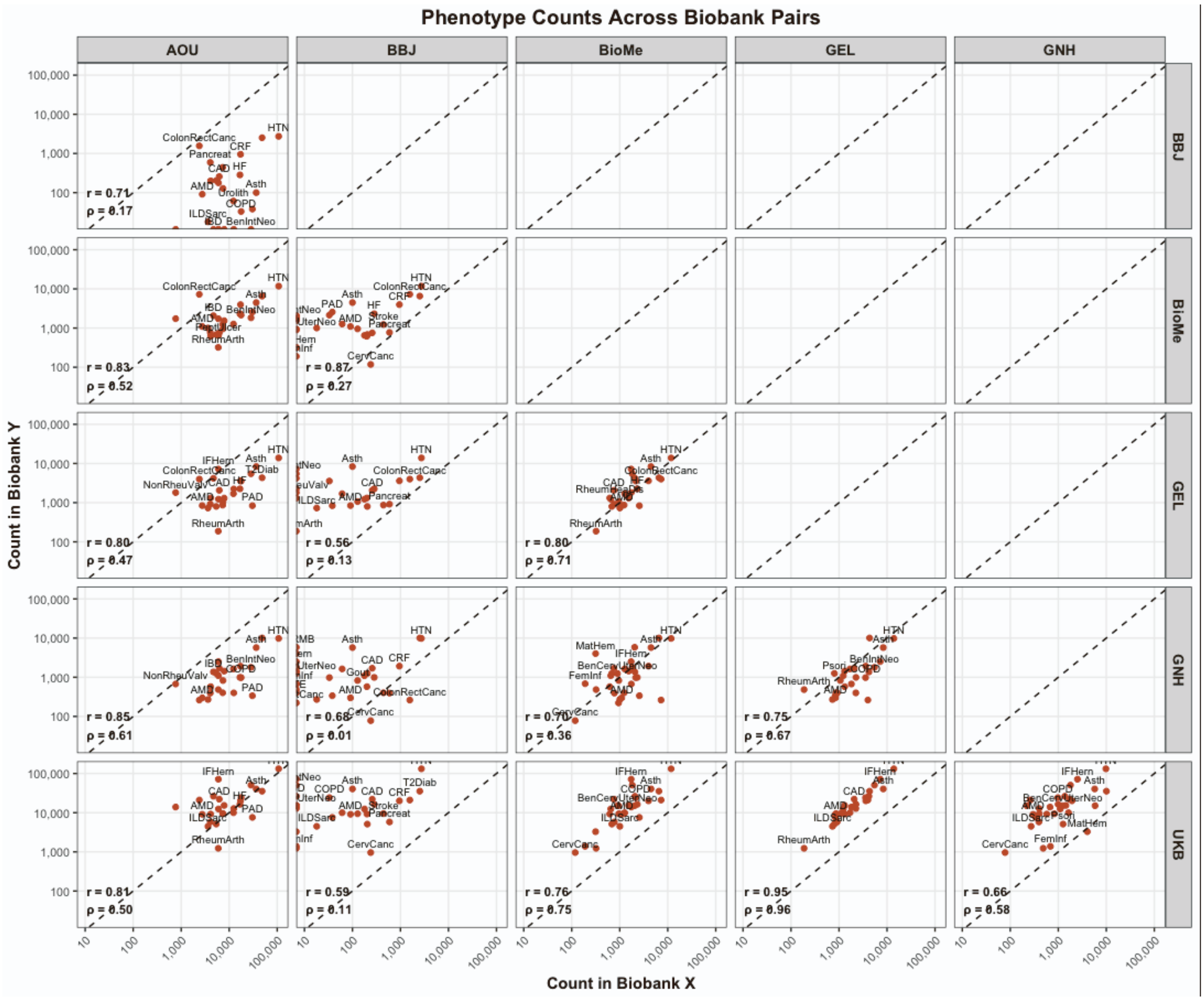

Figure S1: Scatter plot of the pairwise comparison of disease prevalence between biobanks.

The x-axis represents the prevalence of a specific trait in one biobank (labelled along the top), while the y-axis represents the prevalence of the same trait in another biobank (labelled on the right hand side). Each point corresponds to a specific trait aggregated across all ancestry groups. The diagonal dashed line indicates where the prevalence would be equal across biobanks. Pearson (r) and Spearman (ρ) correlation coefficients are shown in each panel, quantifying the strength and direction of the linear and rank-based relationships, respectively. The red dashed line indicates x = y. Please refer to Sup. Table 1 for the complete list of name abbreviations.

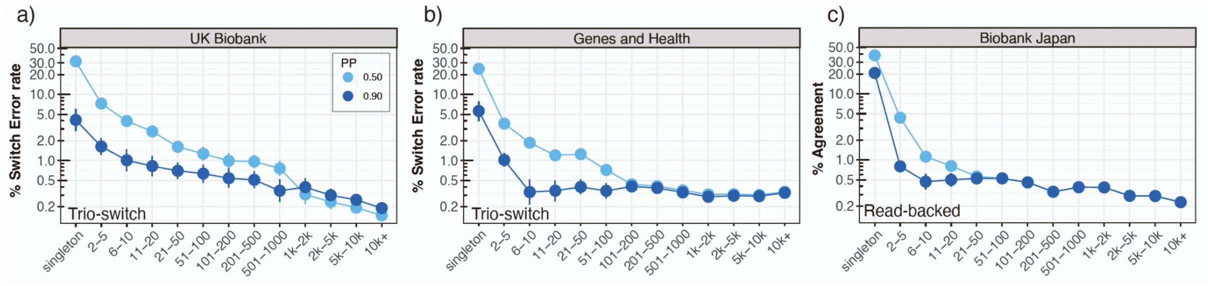

Figure S2: Assessment of phasing accuracy through trio-switch error rates and read-backed phasing.

Phasing accuracy assessment for three biobanks: The *x*-axis represents the MAC bin for the variants, while the *y*-axis shows phasing accuracy, measured by switch errors (a-b) or agreement between read-backed and statistically inferred haplotypes (c). Phasing accuracy is shown before (PP ≥ 0.5) and after (PP ≥ 0.9) filtering to confidently phased variants. Confidence intervals (CIs) indicate 95% binomial confidence intervals. All biobanks used pLoF variants, except for BBJ, which used pLoF plus damaging missense/protein-altering variants due to insufficient CH pLoF variants. Refer to Sup. Table 11 for the underlying data.

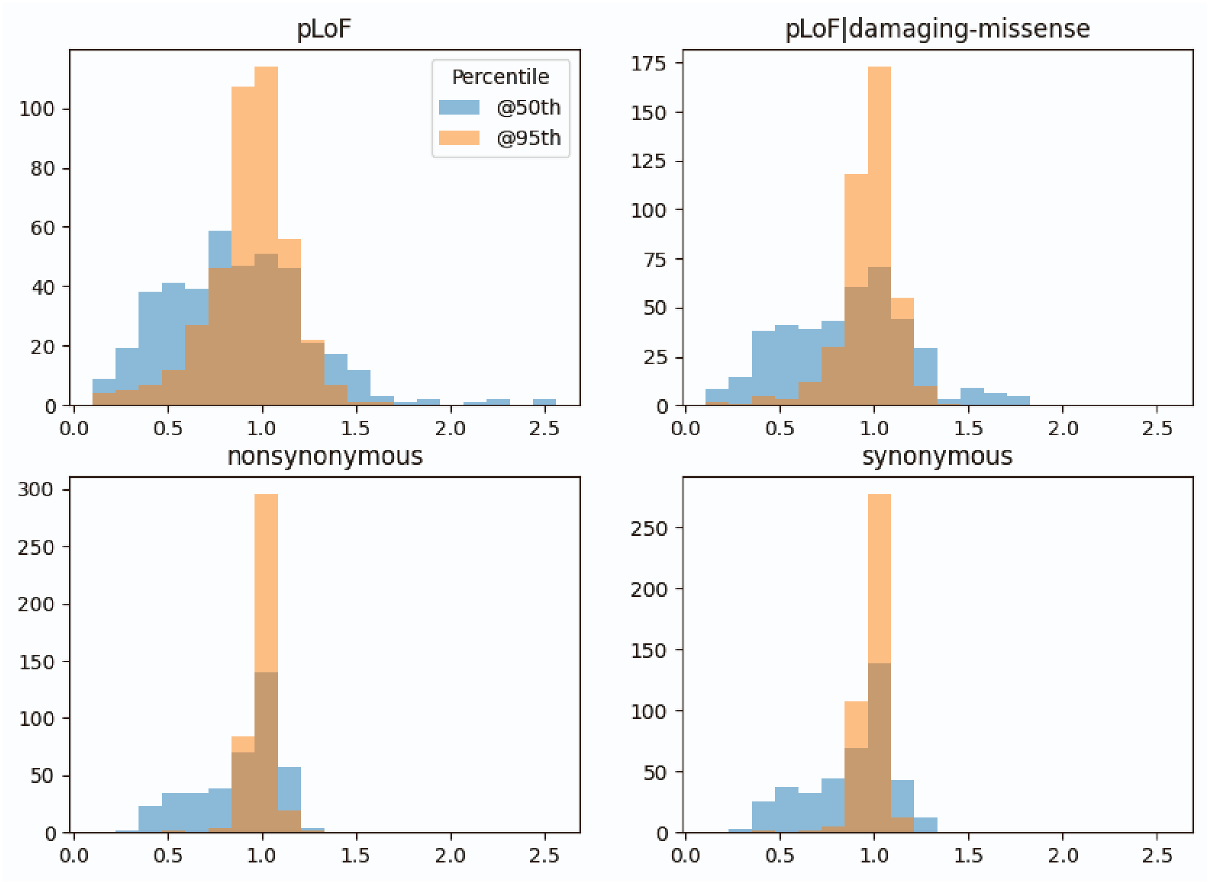

Figure S3: Distribution of inflation factors for recessive tests across all cohorts and phenotypes under consideration.

Histograms showing a comparison of the distribution of the λ_{GC} inflation factors (calculated at the median statistic) with that of λ_{95} (calculated at the 95th percentile) for all recessive tests and each annotation we consider. We note that the maximum values for each annotation are as follows (50th vs 95th): 2.57 vs 1.60 (pLoF), 1.81 vs 1.42 (pLoF|damaging-missense, 1.32 vs 1.27 (nonsynonymous), and 1.30 vs 1.174 (synonymous).

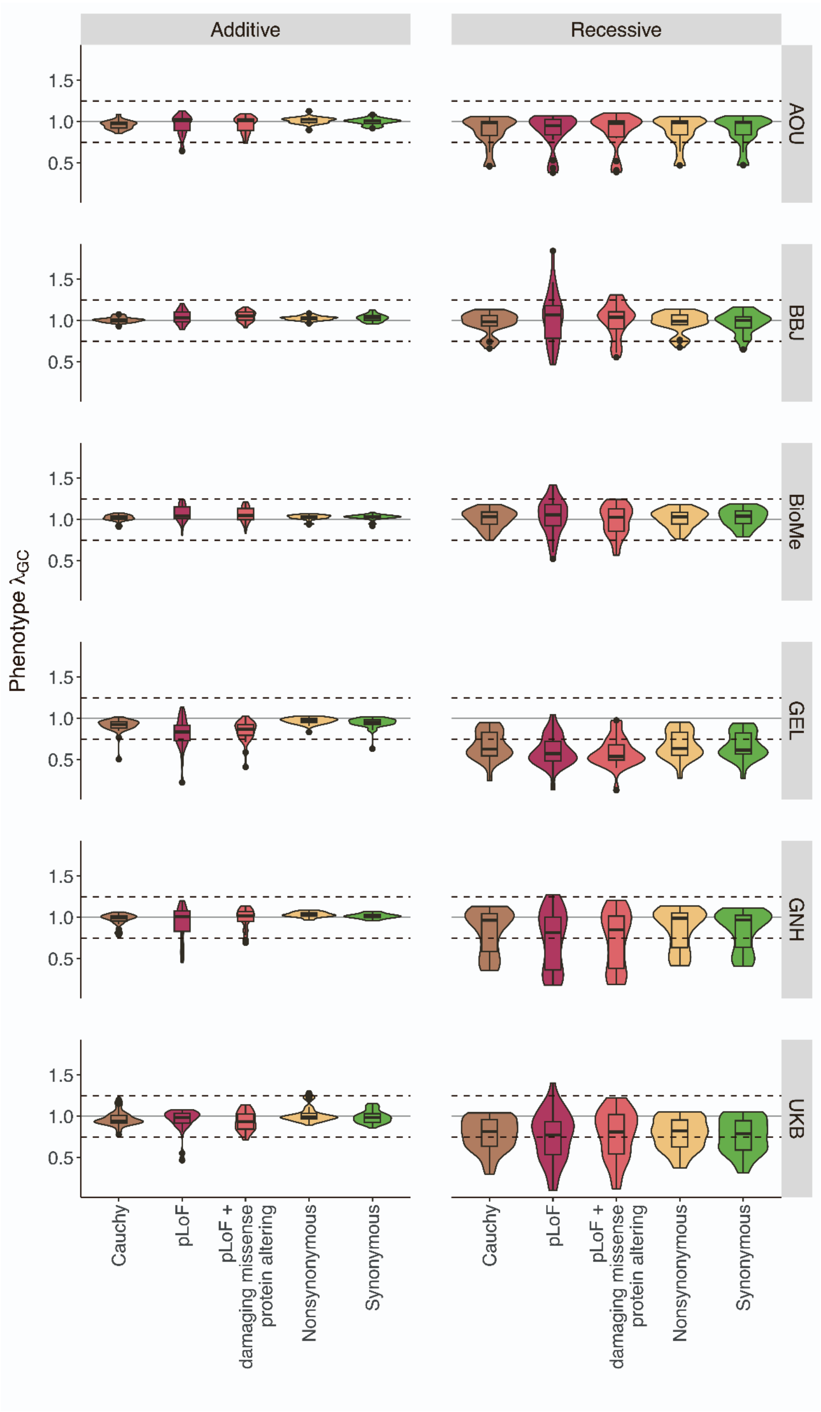

Figure S4: Comparison of test-statistic inflation across biobanks and annotation categories before meta-analysis using λ_{GC}

Violin plots with overlaid boxplots showing the distribution of inflation factors (λ_{GC}) across phenotypes on the y-axis. X-axis represents different variant annotation masks, including Cauchy-combined *P*-values. Dashed lines indicate $\lambda = 1.25$ and $\lambda = 0.75$ cutoffs. Violin shape depicts probability density; boxplot shows median, quartiles (Q1, Q3 as hinges), and whiskers extending to Q1−1.5×interquartile range (IQR) and Q3+1.5×IQR. Outliers beyond whiskers are plotted individually.

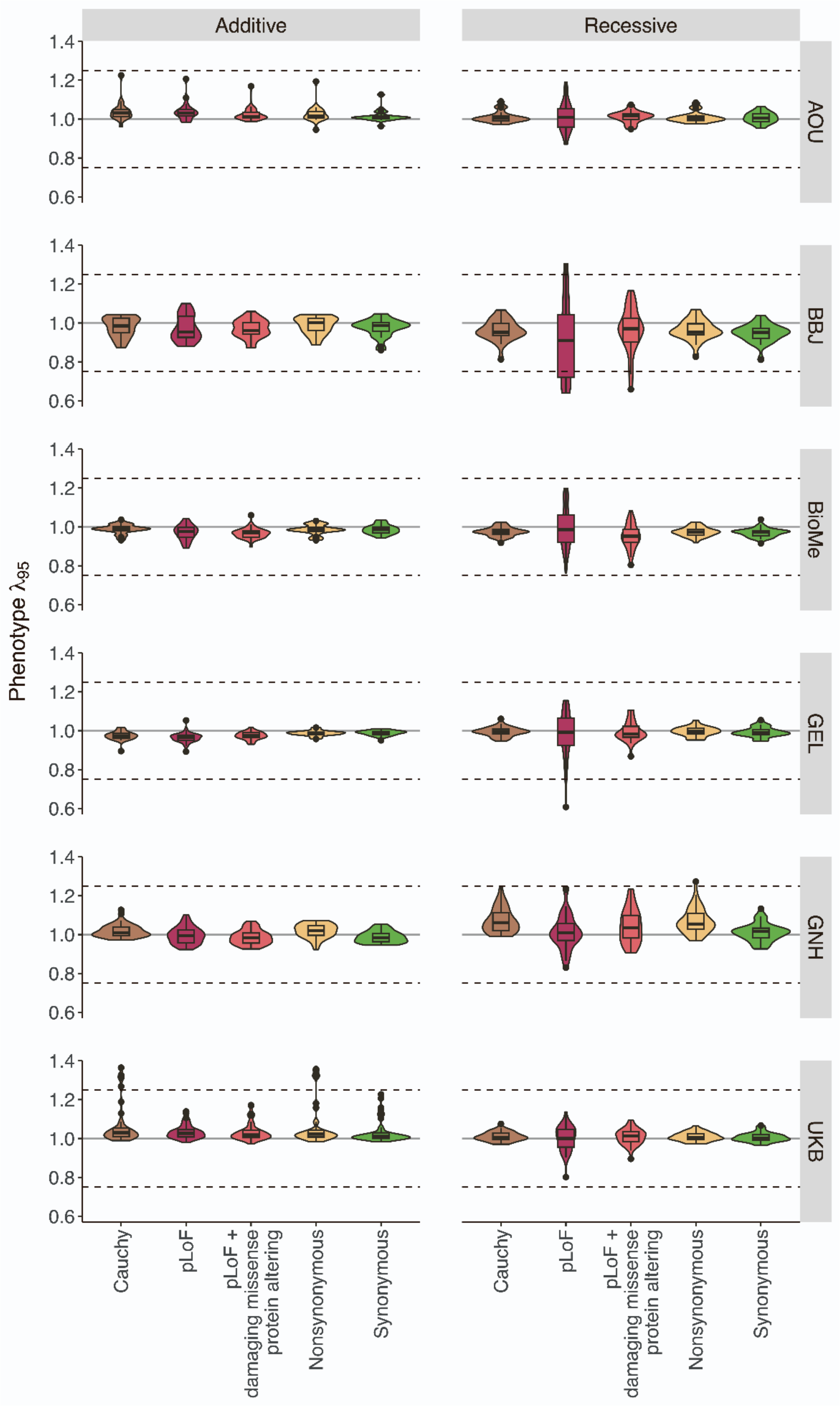

Figure S5: Comparison of test-statistic inflation across biobanks and annotation categories before meta-analysis using λ_{95}.

Violin plots with overlaid boxplots showing the distribution of inflation (λ_{95}, lambda at 95th percentile instead of 50th percentile) on the y-axis. X-axis represents different variant annotation masks, including Cauchy-combined *P*-values. Dashed lines indicate λ=1.25 and λ=0.75 cutoffs. Violin shape depicts probability density; boxplot shows median, quartiles (Q1, Q3 as hinges), and whiskers extending to Q1−1.5×IQR and Q3+1.5×IQR. Outliers beyond whiskers are plotted individually.

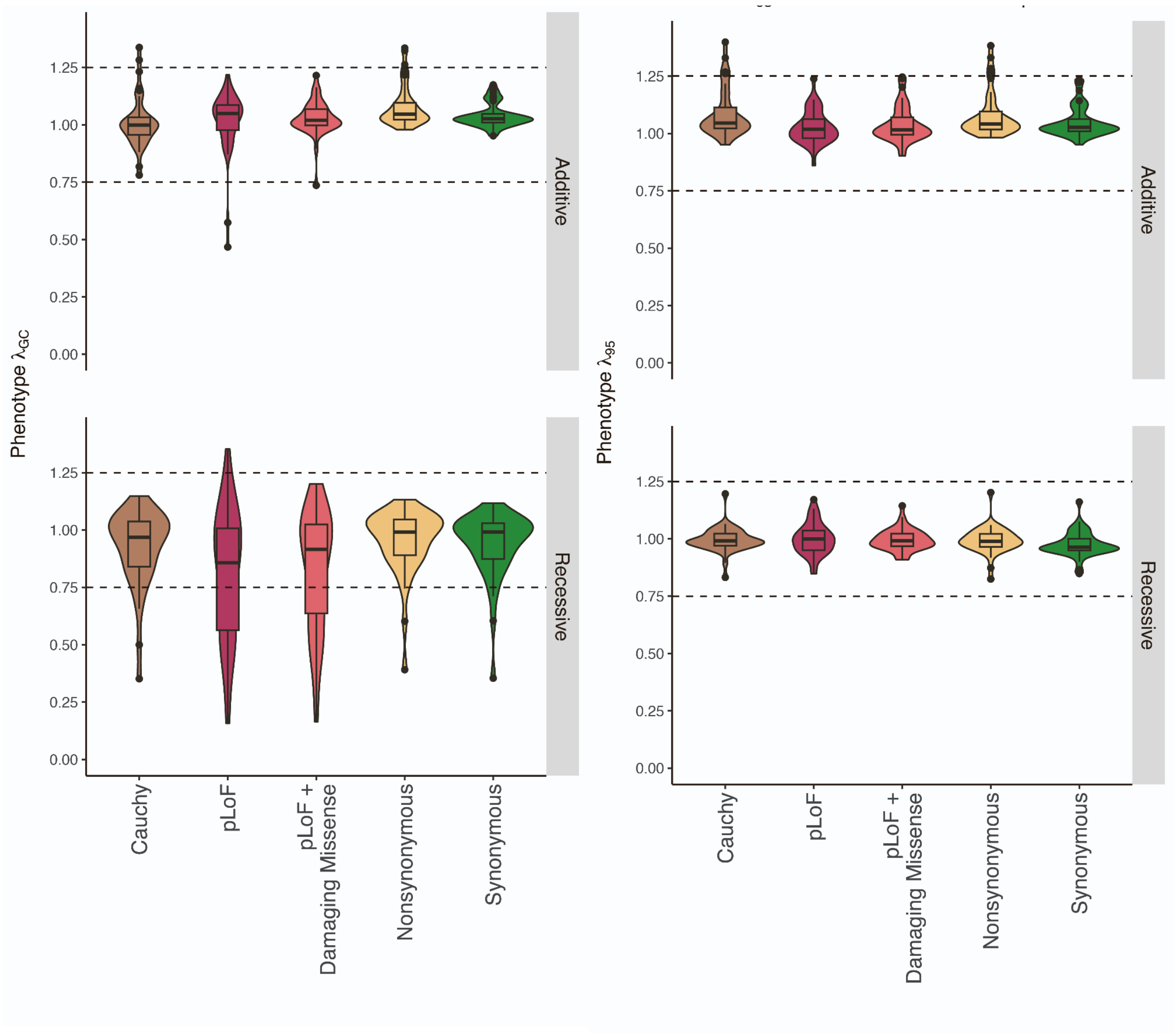

Figure S6: λ_{GC} and λ_{95} for additive and recessive meta-analyses by variant annotation masks.

Violin plots with overlaid boxplots showing the distribution of inflation factors across phenotypes for additive, recessive and nonadditive analyses. Specifically, we consider (**left**) λ_{GC} (genomic control lambda) and (**right**) λ_{95} (lambda estimated at the 95th percentile instead of 50th percentile). The x-axis represents different variant annotation masks, including combinations of *P*-values using the Cauchy distribution. Dashed lines indicate the λ=1.25 and λ=0.75 cutoffs on the y-axis. The violin plot's outer shape depicts the probability density, while the boxplot visualizes the median, quartiles (Q1 and Q3 as hinges), and whiskers extending to Q1−1.5×IQR and Q3+1.5×IQR. Data points beyond the whiskers (outliers) are plotted individually.

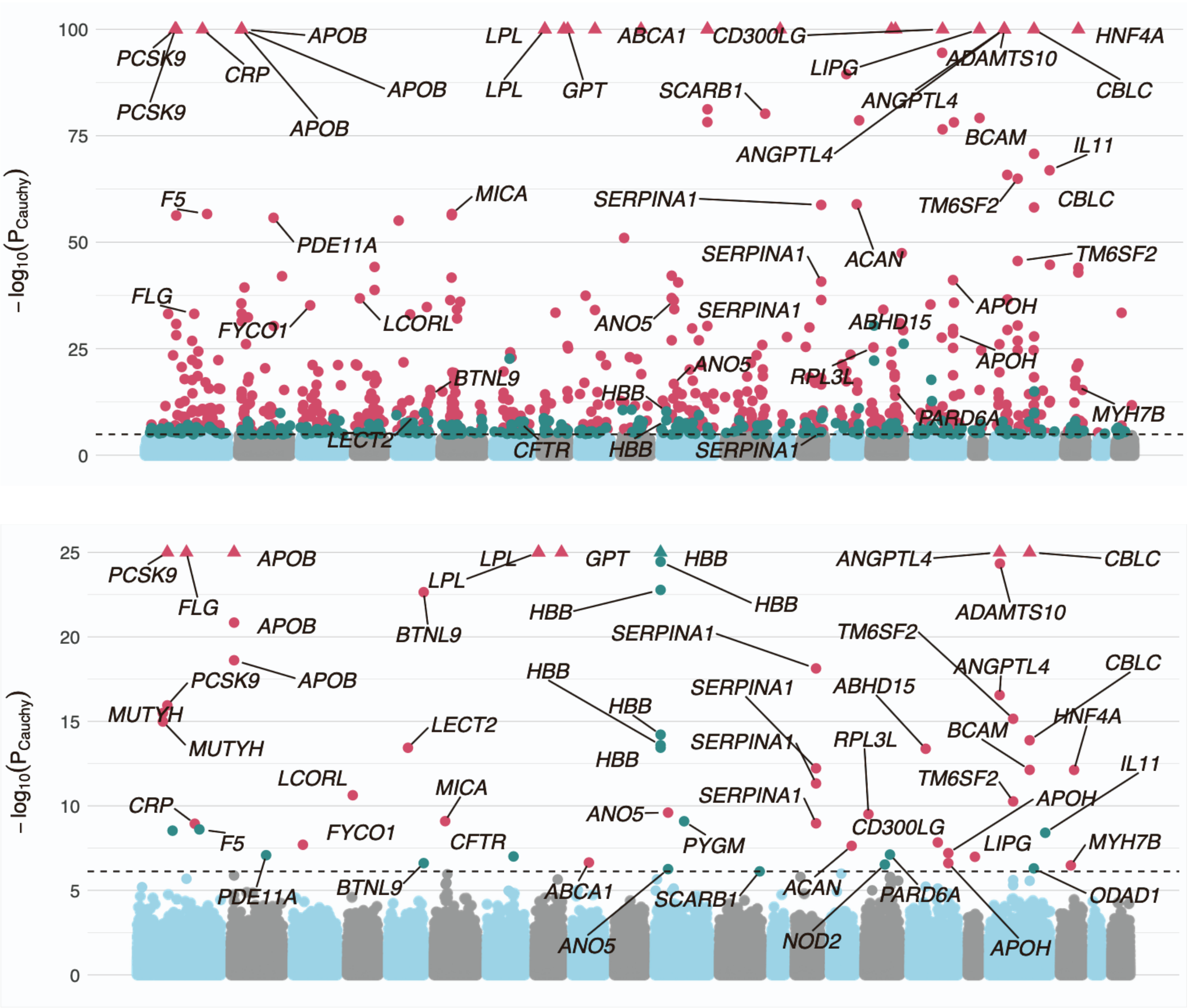

Figure S7: Manhattan plot for additive and recessive meta-analysis.

Manhattan plot for additive and recessive meta-analysis across six biobanks. The meta-analysis was performed first across four different variant annotation masks, followed by a combination of *P*-values using the Cauchy distribution, which are plotted here on the Y-axis. Each point represents a gene-trait association. Points are colored red if the gene-trait associations have been deemed significant (FDR< 0.01) in an analysis of Europeans in UKBB alone, and are otherwise colored green. To aid visualisation, *P*-values are truncated in the additive and recessive facets. Such truncated *P*-values are denoted by a triangle, and non-truncated *P*-values are circles.

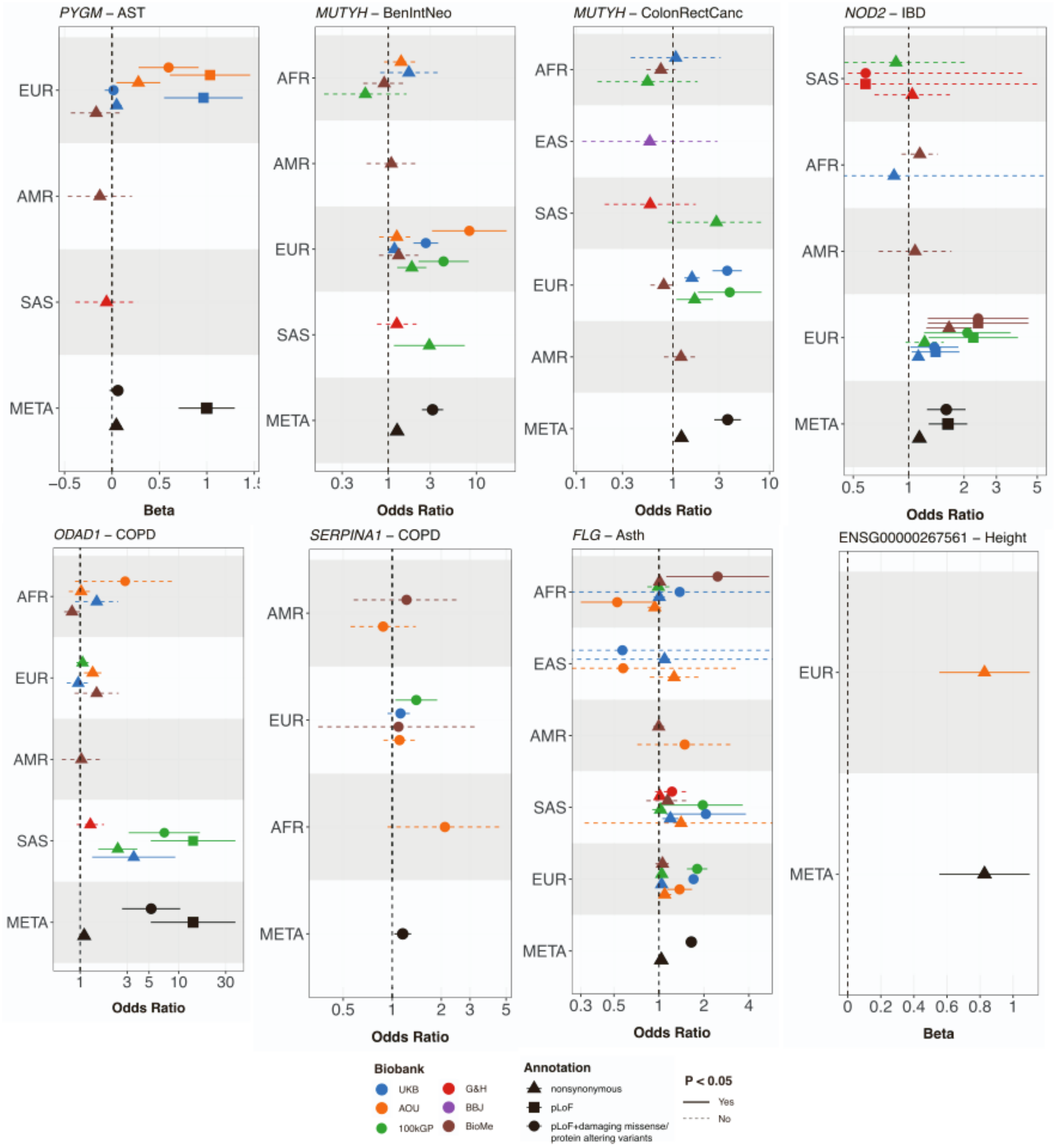

Figure S8: Forest plot of putatively recessive gene-trait associations detected by meta-analysis.

Forest plot of effect sizes or ORs, for quantitative and binary traits respectively, and 95% CIs across significant recessive gene-trait associations (FDR<1% corresponding to $p_{rec} < 7.5 \times 10^{-7}$), in addition to those presented in (main) Figure 5. The plot includes: 1) pLoF variants (squares); 2) pLoF|damaging_missense (circles); and 3) nonsynonymous (triangles), stratified by ancestry, with different biobanks indicated by different colors. We show only associations that we were able to test due to having at least five bi-allelic individuals, and with signals observed in at least two cohorts. To aid visualization, large CIs have been truncated, and dotted lines indicate associations with $p_{rec} > 0.05$. AST: Aspartate aminotransferase; Asth: asthma; please refer to Sup. Table 1 for the complete list of phenotype abbreviations.

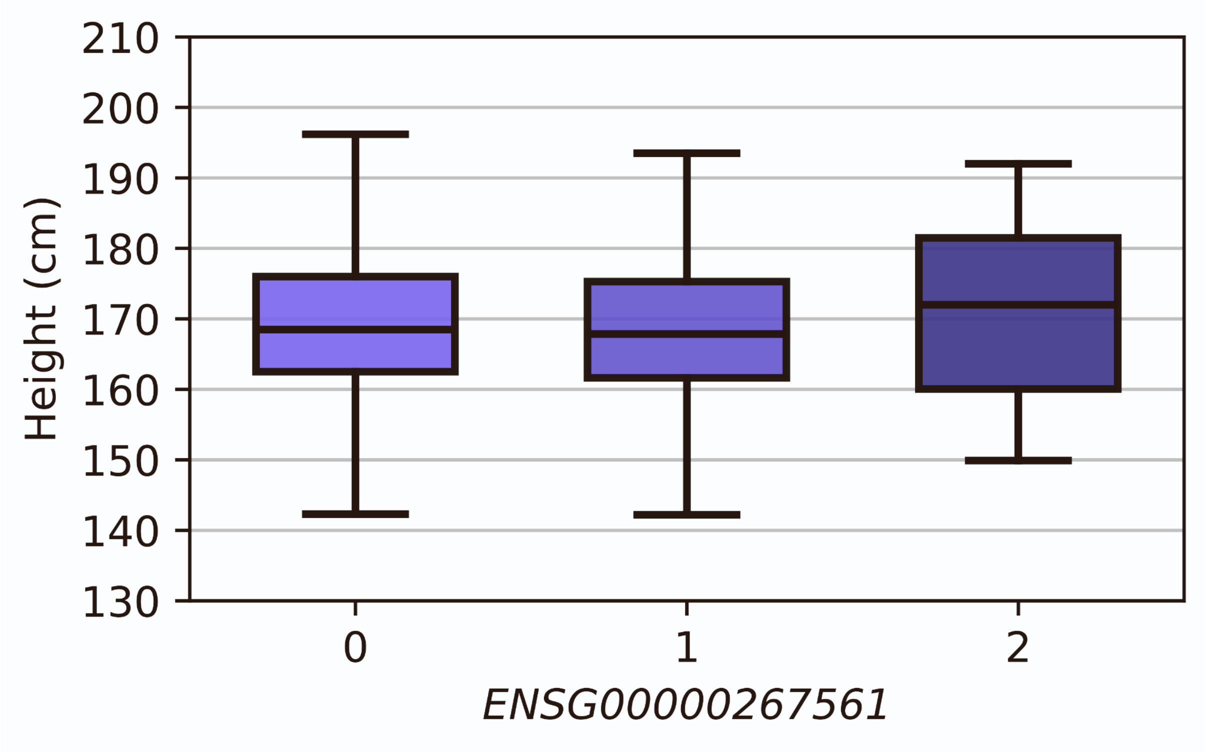

Figure S9: Box plot showing distribution of height (cm) stratified by genotype at ENSG00000267561.

Plot based on nonsynonymous variant burden in AOU:EUR, as that was the only cohort with sufficient number of individuals for association testing (N<20). Individuals are grouped by genotype class: 0 = reference homozygotes, 1 = heterozygotes, and 2 = bi-allelic individuals (homozygous or compound heterozygous). We note that outliers are not shown to comply with privacy rules in AOU.

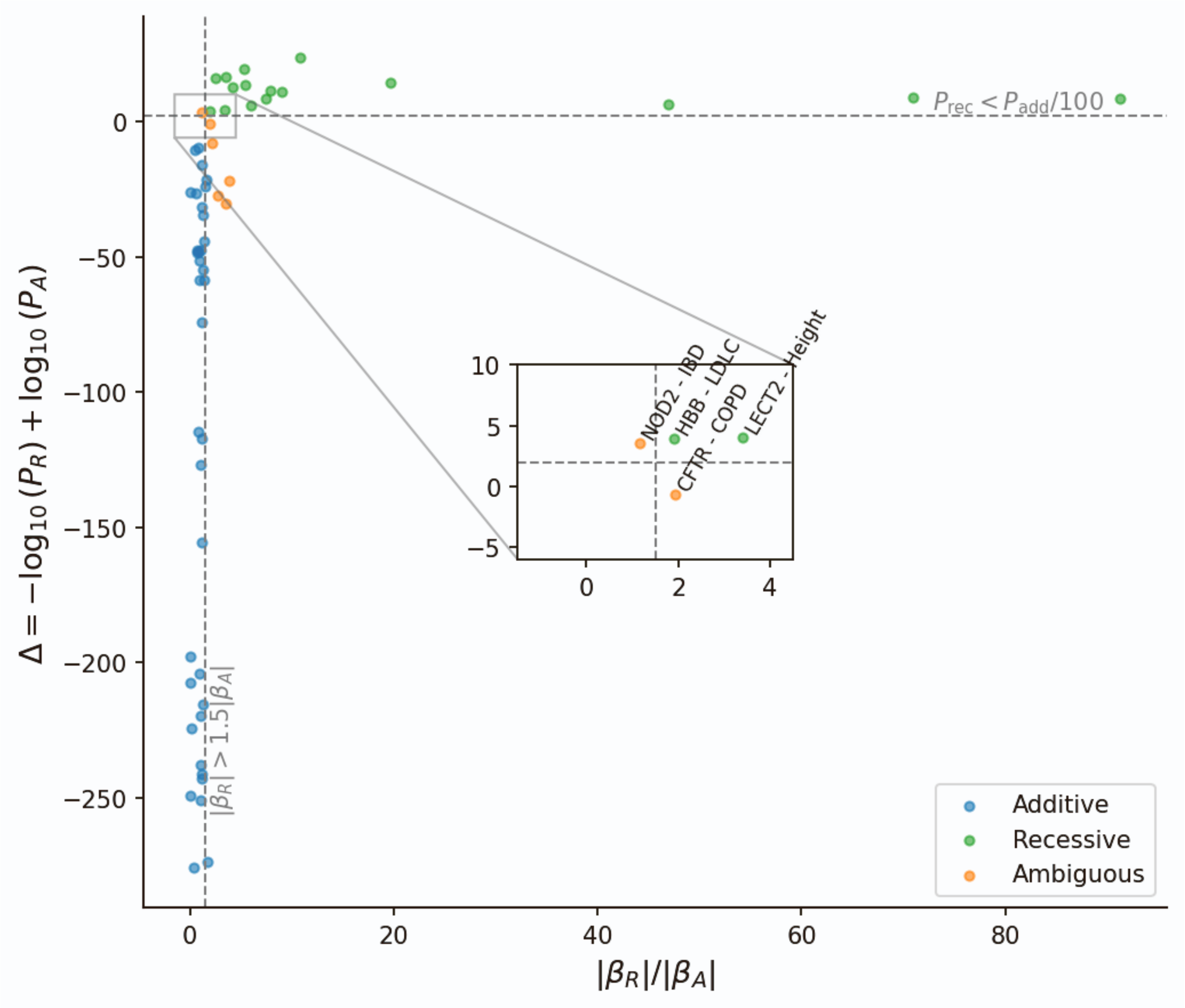

Figure S10: Assessing recessive versus additive architecture

Scatterplot comparing two indicators of inheritance mode, for each significant gene-trait association in our meta-analysis (FDR < 1%), after the Cauchy combination test. On the x-axis we show the absolute recessive effect ($|\beta_R|$) divided by absolute additive effect ($|\beta_A|$) with the vertical dashed line marking an indicative threshold of 1.5 as discussed in Sup. Note 7. On the y-axis we show the difference between $\log_{10}P_A$ and $\log_{10}P_R$, with the horizontal line indicating the threshold of two orders used in our main analysis. Points are coloured by this alternative classification, whereby green = recessive ($\Delta>0$ and $|\beta_R|>1.5|\beta_A|$), blue = additive ($\Delta<0$ and $|\beta_R| < 1.5|\beta_A|$), and orange = ambiguous (do not meet either pair of criteria). The inset enlarges the dense region near the origin and labels the four loci that lie close to the decision boundaries.

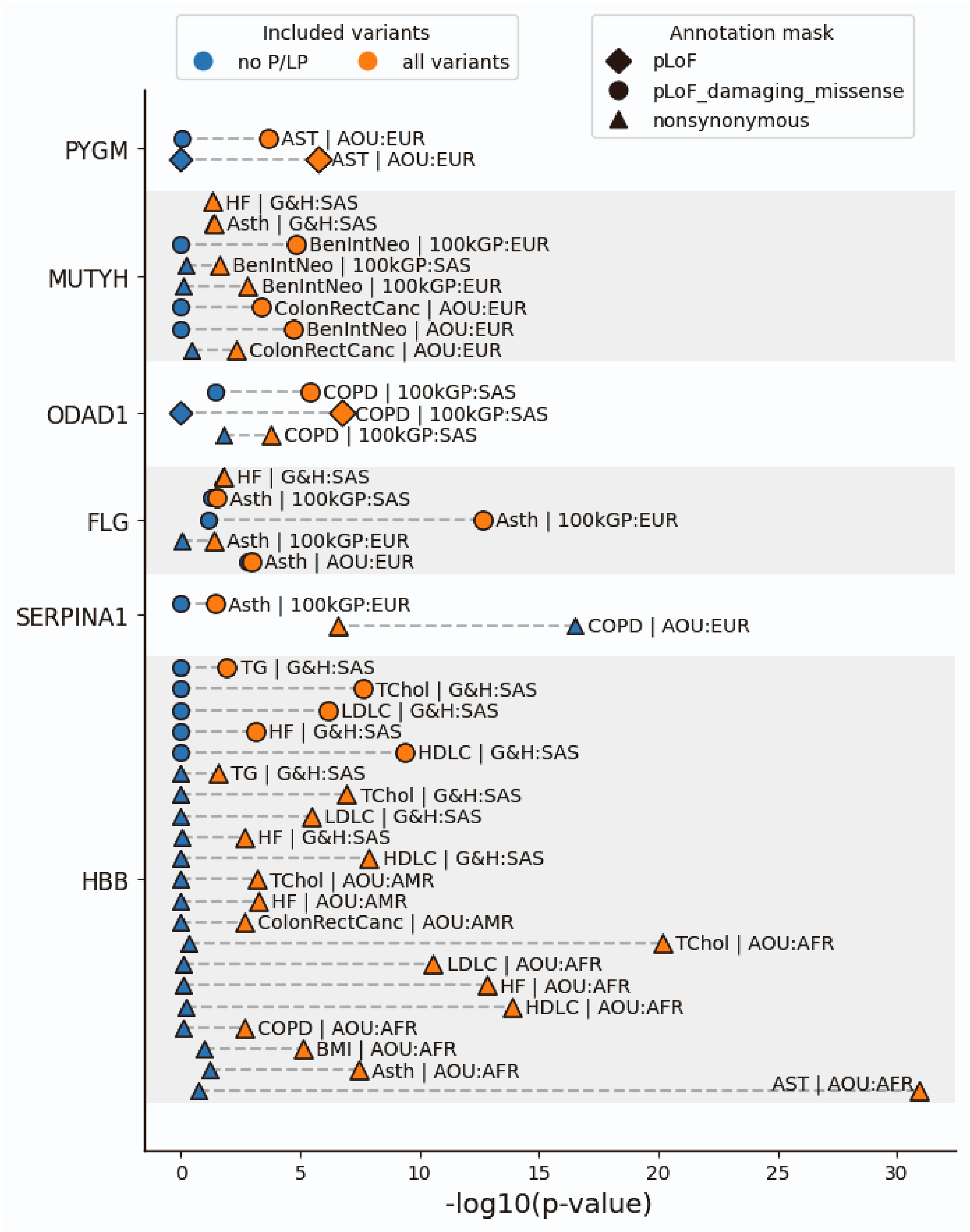

Figure S11: Forest plot summarising the attenuation in association signal after excluding P/LP variants.

We compare the $\log_{10}$ P-values for association testing when using all variants (orange) to those after dropping Pathogenic (P) or Likely-Pathogenic (LP) in ClinVar[31] (blue), stratified by annotation mask and considering genes which are identified with recessive associations in our main analysis. To aid interpretation, a *p*-value of 1.00 is used for cases with < 5 bi-allelic individuals after dropping P/LP variants.

Supplemental References

1. Chen, C.-Y., Chen, T.-T., Anne Feng, Y.-C., Yu, M., Lin, S.-C., Longchamps, R.J., Wang, S.-H., Hsu, Y.-H., Yang, H.-I., Kuo, P.-H., et al. (2024). Analysis across Taiwan Biobank, Biobank Japan, and UK Biobank identifies hundreds of novel loci for 36 quantitative traits. Cell Genom. *4*, 100640. https://doi.org/10.1016/j.xgen.2024.100640.

2. Chen, L., Magliano, D.J., and Zimmet, P.Z. (2011). The worldwide epidemiology of type 2 diabetes mellitus--present and future perspectives. Nat. Rev. Endocrinol. *8*, 228–236. https://doi.org/10.1038/nrendo.2011.183.

3. Gujral, U.P., and Kanaya, A.M. (2021). Epidemiology of diabetes among South Asians in the United States: lessons from the MASALA study. Ann. N. Y. Acad. Sci. *1495*, 24–39. https://doi.org/10.1111/nyas.14530.

4. Carter, P., Gray, L.J., Morris, D.H., Davies, M.J., and Khunti, K. (2013). South Asian individuals at high risk of type 2 diabetes have lower plasma vitamin C levels than white Europeans. J. Nutr. Sci. *2*, e21. https://doi.org/10.1017/jns.2013.15.

5. Bradley, D., and Hsueh, W. (2016). Type 2 diabetes in the elderly: Challenges in a unique patient population. J. Geriatr. Med. Gerontol. *2*. https://doi.org/10.23937/2469-5858/1510014.

6. Dendup, T., Feng, X., Clingan, S., and Astell-Burt, T. (2018). Environmental risk factors for developing type 2 diabetes mellitus: A systematic review. Int. J. Environ. Res. Public Health *15*. https://doi.org/10.3390/ijerph15010078.

7. Gaskin, D.J., Thorpe, R.J., Jr, McGinty, E.E., Bower, K., Rohde, C., Young, J.H., LaVeist, T.A., and Dubay, L. (2014). Disparities in diabetes: the nexus of race, poverty, and place. Am. J. Public Health *104*, 2147–2155. https://doi.org/10.2105/AJPH.2013.301420.

8. GBD Results Institute for Health Metrics and Evaluation. https://vizhub.healthdata.org/gbd-results/.

9. Caulfield, M., Davies, J., Dennys, M., Elbahy, L., Fowler, T., Hill, S., Hubbard, T., Jostins, L., Maltby, N., Mahon-Pearson, J., et al. (2020). National Genomic Research Library. (figshare). https://doi.org/10.6084/M9.FIGSHARE.4530893.V7 https://doi.org/10.6084/M9.FIGSHARE.4530893.V7.

10. Sosinsky, A., Ambrose, J., Cross, W., Turnbull, C., Henderson, S., Jones, L., Hamblin, A., Arumugam, P., Chan, G., Chubb, D., et al. (2024). Insights for precision oncology from the integration of genomic and clinical data of 13,880 tumors from the 100,000 Genomes Cancer Programme. Nat. Med. *30*, 279–289. https://doi.org/10.1038/s41591-023-02682-0.

11. Shi, S., Rubinacci, S., Hu, S., Moutsianas, L., Stuckey, A., Need, A.C., Palamara, P.F., Caulfield, M., Marchini, J., and Myers, S. (2024). A Genomics England haplotype reference panel and imputation of UK Biobank. Nat. Genet. *56*, 1800–1803. https://doi.org/10.1038/s41588-024-01868-7.

12. Karczewski, K.J., Francioli, L.C., Tiao, G., Cummings, B.B., Alföldi, J., Wang, Q., Collins, R.L., Laricchia, K.M., Ganna, A., Birnbaum, D.P., et al. (2020). The mutational constraint spectrum quantified from variation in 141,456 humans. Nature *581*, 434–443. https://doi.org/10.1038/s41586-020-2308-7.

13. Lim, E.T., Raychaudhuri, S., Sanders, S.J., Stevens, C., Sabo, A., MacArthur, D.G., Neale, B.M., Kirby, A., Ruderfer, D.M., Fromer, M., et al. (2013). Rare complete knockouts in humans: population distribution and significant role in autism spectrum disorders. Neuron *77*, 235–242. https://doi.org/10.1016/j.neuron.2012.12.029.

14. Martin, M., Patterson, M., Garg, S., O Fischer, S., Pisanti, N., Klau, G.W., Schöenhuth, A., and Marschall, T. (2016). WhatsHap: fast and accurate read-based phasing. bioRxiv. https://doi.org/10.1101/085050.

15. Sulem, P., Helgason, H., Oddson, A., Stefansson, H., Gudjonsson, S.A., Zink, F., Hjartarson, E., Sigurdsson, G.T., Jonasdottir, A., Jonasdottir, A., et al. (2015). Identification of a large set of rare complete human knockouts. Nat. Genet. *47*, 448–452. https://doi.org/10.1038/ng.3243.

16. Narasimhan, V.M., Hunt, K.A., Mason, D., Baker, C.L., Karczewski, K.J., Barnes, M.R., Barnett, A.H., Bates, C., Bellary, S., Bockett, N.A., et al. (2016). Health and population effects of rare gene knockouts in adult humans with related parents. Science *352*, 474–477. https://doi.org/10.1126/science.aac8624.

17. Saleheen, D., Natarajan, P., Armean, I.M., Zhao, W., Rasheed, A., Khetarpal, S.A., Won, H.-H., Karczewski, K.J., O'Donnell-Luria, A.H., Samocha, K.E., et al. (2017). Human knockouts and phenotypic analysis in a cohort with a high rate of consanguinity. Nature *544*, 235–239. https://doi.org/10.1038/nature22034.

18. Oddsson, A., Sulem, P., Sveinbjornsson, G., Arnadottir, G.A., Steinthorsdottir, V., Halldorsson, G.H., Atlason, B.A., Oskarsson, G.R., Helgason, H., Nielsen, H.S., et al. (2023). Deficit of homozygosity among 1.52 million individuals and genetic causes of recessive lethality. Nat. Commun. *14*, 3453. https://doi.org/10.1038/s41467-023-38951-2.

19. Sun, K.Y., Bai, X., Chen, S., Bao, S., Zhang, C., Kapoor, M., Backman, J., Joseph, T., Maxwell, E., Mitra, G., et al. (2024). A deep catalogue of protein-coding variation in 983,578 individuals. Nature. https://doi.org/10.1038/s41586-024-07556-0.

20. Malawsky, D.S., van Walree, E., Jacobs, B.M., Heng, T.H., Huang, Q.Q., Sabir, A.H., Rahman, S., Sharif, S.M., Khan, A., Mirkov, M.U., et al. (2023). Influence of autozygosity on common disease risk across the phenotypic spectrum. medRxiv. https://doi.org/10.1101/2023.02.01.23285346.

21. Jurgens, S.J., Wang, X., Choi, S.H., Weng, L.-C., Koyama, S., Pirruccello, J.P., Nguyen, T., Smadbeck, P., Jang, D., Chaffin, M., et al. (2024). Rare coding variant analysis for human diseases across biobanks and ancestries. Nat. Genet. *56*, 1811–1820. https://doi.org/10.1038/s41588-024-01894-5.

22. Yang, J., Weedon, M.N., Purcell, S., Lettre, G., Estrada, K., Willer, C.J., Smith, A.V., Ingelsson, E., O'Connell, J.R., Mangino, M., et al. (2011). Genomic inflation factors under polygenic inheritance. Eur. J. Hum. Genet. *19*, 807–812. https://doi.org/10.1038/ejhg.2011.39.

23. Sohail, M., Maier, R.M., Ganna, A., Bloemendal, A., Martin, A.R., Turchin, M.C., Chiang, C.W., Hirschhorn, J., Daly, M.J., Patterson, N., et al. (2019). Polygenic adaptation on height is overestimated due to uncorrected stratification in genome-wide association studies. Elife *8*. https://doi.org/10.7554/eLife.39702.

24. Clark, D.W., Okada, Y., Moore, K.H.S., Mason, D., Pirastu, N., Gandin, I., Mattsson, H., Barnes, C.L.K., Lin, K., Zhao, J.H., et al. (2019). Associations of autozygosity with a broad range of human phenotypes. Nat. Commun. *10*, 4957. https://doi.org/10.1038/s41467-019-12283-6.

25. Karczewski, K.J., Solomonson, M., Chao, K.R., Goodrich, J.K., Tiao, G., Lu, W., Riley-Gillis, B.M., Tsai, E.A., Kim, H.I., Zheng, X., et al. (2022). Systematic single-variant and gene-based association testing of thousands of phenotypes in 394,841 UK Biobank exomes. Cell Genom. *2*, 100168. https://doi.org/10.1016/j.xgen.2022.100168.

26. Onoufriadis, A., Paff, T., Antony, D., Shoemark, A., Micha, D., Kuyt, B., Schmidts, M., Petridi, S., Dankert-Roelse, J.E., Haarman, E.G., et al. (2013). Splice-site mutations in the axonemal outer dynein arm docking complex gene CCDC114 cause primary ciliary dyskinesia. Am. J. Hum. Genet. *92*, 88–98. https://doi.org/10.1016/j.ajhg.2012.11.002.

27. Knowles, M.R., Leigh, M.W., Ostrowski, L.E., Huang, L., Carson, J.L., Hazucha, M.J., Yin, W., Berg, J.S., Davis, S.D., Dell, S.D., et al. (2013). Genetic Disorders of Mucociliary Clearance Consortium, Exome sequencing identifies mutations in CCDC114 as a cause of primary ciliary dyskinesia. Am. J. Hum. Genet *92*, 99–106.

28. Guichard, C., Harricane, M.C., Lafitte, J.J., Godard, P., Zaegel, M., Tack, V., Lalau, G., and

Bouvagnet, P. (2001). Axonemal dynein intermediate-chain gene (DNAI1) mutations result in situs inversus and primary ciliary dyskinesia (Kartagener syndrome). Am. J. Hum. Genet. *68*, 1030–1035. https://doi.org/10.1086/319511.

29. Hannah, W.B., Derks, T.G.J., Drumm, M.L., Grünert, S.C., Kishnani, P.S., and Vissing, J. (2023). Glycogen storage diseases. Nat. Rev. Dis. Primers *9*.

30. Heyne, H.O., Karjalainen, J., Karczewski, K.J., Lemmelä, S.M., Zhou, W., FinnGen, Havulinna, A.S., Kurki, M., Rehm, H.L., Palotie, A., et al. (2023). Mono- and biallelic variant effects on disease at biobank scale. Nature *613*, 519–525. https://doi.org/10.1038/s41586-022-05420-7.

31. Landrum, M.J., Lee, J.M., Benson, M., Brown, G., Chao, C., Chitipiralla, S., Gu, B., Hart, J., Hoffman, D., Hoover, J., et al. (2016). ClinVar: public archive of interpretations of clinically relevant variants. Nucleic Acids Res. *44*, D862–D868. https://doi.org/10.1093/nar/gkv1222.
